# A lightweight tri-modal few-shot detection framework for fruit diversity recognition toward digital orchard archiving

**DOI:** 10.3389/fpls.2025.1696622

**Published:** 2025-12-01

**Authors:** Huaqiang Xu, Honghan Li, Ji Zhao

**Affiliations:** School of Computer Science and Software Engineering, University of Science and Technology, Liaoning, Anshan, China

**Keywords:** fruit detection, digital orchard, FSOD, CLIP prompt, SAM mask, multimodal fusion, attention weighting, lightweight agriculture AI

## Abstract

Few-shot object detection (FSOD) addresses the challenge of object recognition under limited annotation conditions, offering practical advantages for smart agriculture, where large-scale labeling of diverse fruit cultivars is often infeasible. To handle the visual complexity of orchard environments—such as occlusion, subtle morphological differences, and dense foliage—this study presents a lightweight tri-modal fusion framework. The model initially employs a CLIP-based semantic prompt encoder to extract category-aware cues, which guide the Segment Anything Model (SAM) in producing structure-preserving masks. These masks are then incorporated via a Semantic Fusion Module (SFM): a Mask-Saliency Adapter (MSA) and a Feature Enhancement Recomposer (FER), enabling spatially aligned and semantically enriched feature modulation. An Attention-Aware Weight Estimator (AWE) further optimizes the fusion by adaptively balancing semantic and visual streams using global saliency cues. The final predictions are subsequently generated by a YOLOv12 detection head. Experiments conducted on four fruit detection benchmarks—Cantaloupe.v2, Peach.v3, Watermelon.v2, and Orange.v8—demonstrate that the proposed method consistently surpasses five representative FSOD baselines. Performance improvements include +7.9% AP@0.5 on Cantaloupe.v2, +5.4% Precision on Peach.v3, +7.4% Precision on Watermelon.v2, and +5.9% AP@0.75 on Orange.v8. These results underscore the model’s effectiveness in orchard-specific scenarios and its potential to facilitate cultivar identification, digital recordkeeping, and cost-efficient agricultural monitoring.

## 1 Introduction

Monitoring crop diversity is essential to modern agricultural management, supporting tasks such as variety identification, digital archiving, germplasm registration, and supply chain traceability. In particular, the rapid detection and classification of fruit cultivars—based on visual data collected from orchards, packing lines, or field surveys—constitutes a key step toward intelligent orchard systems and digital agronomy platforms (Chen et al., 2025; Ndikumana et al., 2024; Li et al., 2024; Luo et al., 2024). These visual pipelines enable automated variety inventory, reduce manual annotation costs, and provide scalable tools for monitoring fruit appearance, harvest readiness, and genetic diversity over time. With the increasing emphasis on smart agriculture and climate-resilient planning, there is growing demand for accurate and lightweight fruit recognition systems, particularly under field conditions where data are sparsely labeled and image acquisition is unconstrained (Liu et al., 2023; Maheswari et al., 2021; Liu et al., 2020; Jia et al., 2022).

Early efforts in fruit diversity recognition primarily focused on deep learning-based visual models, which employed convolutional neural networks (CNNs) to extract traits such as shape, color, and texture for cultivar classification (Sun et al., 2024; Ma et al., 2023). These approaches have been successfully applied to fruit sorting, variety labeling, and cultivar cataloging, often serving as the basis for digital orchard archiving systems. However, their dependence on large-scale labeled datasets and fixed category sets limits adaptability to novel cultivars and rare landraces. Moreover, RGB-based appearance features alone struggle to differentiate visually similar fruits, especially under challenging orchard conditions with occlusion, variable lighting, and morphological changes (Tao et al., 2024; Huang et al., 2024). These limitations highlight the need for recognition frameworks that are lightweight, scalable, and capable of generalizing to unseen varieties.

General-purpose object detectors, such as Faster R-CNN, SSD, and YOLO, have also been widely applied in agricultural scenarios for fruit localization and classification tasks (Hu et al., 2023; Wang et al., 2025). Although transfer learning, data augmentation, and lightweight backbones have been explored to adapt these models to orchard imagery, their performance often deteriorates in the presence of subtle inter-class variations, partial occlusion, and cluttered natural backgrounds. Furthermore, they typically require densely annotated bounding boxes and lack the ability to incorporate contextual or semantic priors, making them poorly suited for fine-grained cultivar recognition under limited supervision (Luo et al., 2025; Lipiński et al., 2025). These drawbacks restrict their scalability in real-world digital orchard systems.

Few-shot object detection (FSOD) has recently emerged as a practical solution for agricultural recognition tasks with limited annotations (Lim et al., 2025; Zhu and Zhang, 2024). Existing FSOD research primarily follows two directions: meta-learning approaches that generalize across categories via episodic training, and augmentation-based strategies that enhance the support set through geometric transforms, domain mixing, or texture replacement (Xin et al., 2024; Han and Lim, 2024). While effective in generic benchmarks such as COCO and PASCAL VOC, many of these methods struggle when applied to orchard environments. Agricultural imagery often exhibits subtle inter-class similarity, high intra-class variation across growth stages, and frequent occlusion by leaves or branches—factors not fully captured in conventional FSOD pipelines (He et al., 2025; Guo et al., 2025). Additionally, the reliance on large backbones and multi-stage designs increases computational overhead, limiting applicability in resource-constrained farming environments.

Despite progress in agricultural computer vision and few-shot object detection, significant gaps remain. Existing frameworks are often adapted from general-purpose datasets, lack mechanisms to incorporate structural priors, and exhibit computational inefficiency for edge deployment in orchards. Moreover, few studies have explored integrating cross-modal priors—such as text prompts from CLIP (Radford et al., 2021) or structural masks from SAM (Kirillov et al., 2023)—into agricultural FSOD tasks. This gap motivates the development of lightweight, semantically guided detection frameworks that can robustly recognize fruit diversity under weak supervision and challenging field conditions.

**Figure 1:**
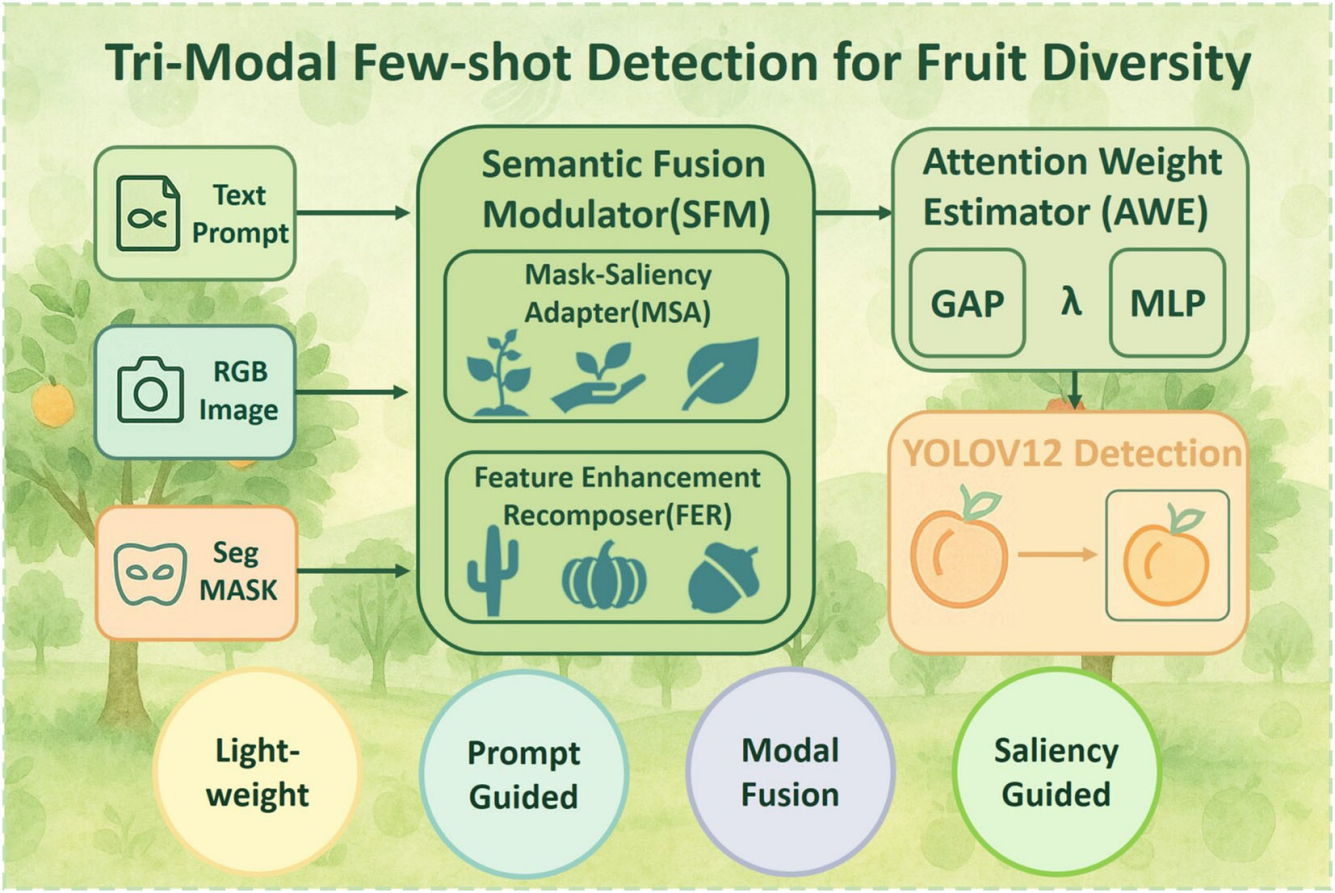
Tri-modal few-shot detection for fruit diversity.

To address the challenges of visual ambiguity and data sparsity in fruit diversity recognition, we propose a lightweight tri-modal few-shot detection framework tailored for agricultural species classification and digital orchard archiving. Unlike previous works that depend solely on RGB-based features or class-prototype matching, our method introduces a multi-stage, semantically grounded pipeline that integrates textual priors, segmentation masks, and visual representations into a unified detection process, as illustrated in **Figure 1**. This design is well-suited for distinguishing subtle fruit categories (e.g. peaches, cantaloupes, watermelons), which frequently involve inter-class similarity, occlusion, and scale variation in orchard settings.

At the core of the framework is a prompt-guided semantic generation mechanism. Given user-defined fruit names (e.g.”peach”, “orange”), a CLIP-based cross-modal similarity engine encodes the text and guides the extraction of spatial prompts (Radford et al., 2021; Zhang et al., 2025; Yu et al., 2025). These prompts are fed into the Segment Anything Model (SAM) (Kirillov et al., 2023; Mazurowski et al., 2023; Osco et al., 2023; Archit et al., 2025) to produce class-agnostic masks that highlight fruit-related regions. The resulting masks serve as category-aware spatial priors and are fused with RGB features via a two-stage Semantic Fusion Modulator (SFM). Specifically, the Semantic Fusion Modulator (SFM) consists of two submodules: the Mask-Saliency Adapter (MSA), which generates sparse attention maps from the segmentation masks to direct the model’s focus, and the Feature Enhancement Recomposer (FER), which modulates the RGB features to reinforce structural and contextual cues.

To mitigate the influence of potential noise from external priors and regulate the contribution of heterogeneous inputs, we incorporate an Attention-Aware Weight Estimator (AWE). This component employs a lightweight multi-layer perceptron (MLP) to dynamically compute fusion weights based on global saliency statistics, allowing the model to balance semantic-enhanced and appearance-based features in a data-driven manner. AWE improves robustness under ambiguous visual conditions and supports better generalization across diverse fruit types and imaging scenarios. The final detection is performed using a standard YOLOv12 head, ensuring high inference efficiency without sacrificing recognition accuracy.

Our main contributions are summarized as follows:

- We present a tri-modal few-shot detection framework that integrates visual, textual, and structural modalities, designed for low-data fruit classification in agricultural environments. The framework is efficient, modular, and compatible with lightweight detection backbones.
- We propose a two-stage Semantic Fusion Modulator (SFM), consisting of the Mask-Saliency Adapter (MSA) and Feature Enhancement Recomposer (FER), which improves attention localization and structural discrimination in challenging scenarios with subtle inter-class differences.
- We introduce an independent Attention-Aware Weight Estimator (AWE) that adaptively balances semantic and appearance features via learned fusion weights, improving robustness under weak supervision and visual complexity.

The proposed framework offers a modular and interpretable solution to few-shot fruit detection, narrowing the gap between open-vocabulary priors and anchor-based detectors. It supports intelligent orchard applications by enabling scalable and fine-grained classification for digital archiving, species monitoring, and agricultural resource tracking.

## 2 Materials and methods

**Figure 2:**
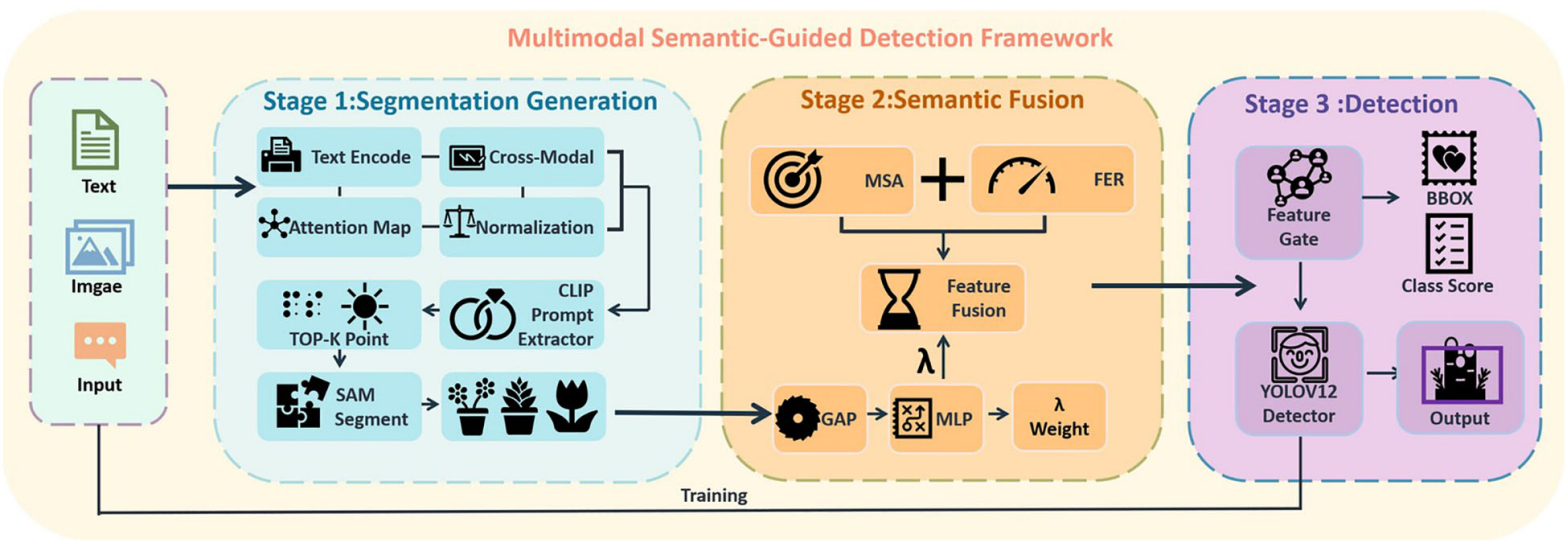
Multimodal semantic-guided detection framework.

To address the challenges arising from sparse annotations and modality imbalance in few-shot object detection (FSOD), we propose a prompt-guided multimodal detection framework, as illustrated in **Figure 2**. The architecture consists of four principal components: a CLIP-assisted prompt extractor that identifies category-relevant regions through cross-modal similarity; a segmentation-based semantic generator (SAM) that produces class-agnostic masks based on the extracted prompts; a Semantic Fusion Modulator (SFM), which integrates visual and semantic information via two lightweight modules—the Mask-Saliency Adapter (MSA) and the Feature Enhancement Recomposer (FER); and an Attention-Aware Weight Estimator (AWE), which adaptively balances the contributions of semantic-enhanced and original features during inference. The final detection is performed using the standard YOLOv12 detection head, preserving the original architecture to ensure high inference efficiency.

The core idea is to use text-conditioned semantic localization to guide the generation of segmentation masks, which are then utilized to refine and modulate the RGB feature representations. This two-stage modulation enhances object-specific features and improves detection robustness under limited supervision.

### 2.1 Overview of the prompt-guided multimodal detection framework

As illustrated in **Figure 2**, the proposed framework consists of three cooperative components, forming a prompt-guided multimodal detection pipeline.

First, the Text-Guided Prompt Generator employs a CLIP-based visual-language encoder to convert user-defined object descriptions—such as *“peach”* or *“watermelon”*—into semantic embeddings. These embeddings are matched with image regions to identify spatially relevant points, which serve as input prompts for the segmentation module. This process enables selective emphasis on target objects while suppressing irrelevant background, improving the precision of subsequent mask generation and fusion.

Second, the Semantic Prior Generation Module (SPGM) utilizes the CLIP-derived point prompts to guide zero-shot segmentation. The output is a class-agnostic mask *M* that captures structural priors aligned with the semantic intent, offering spatially coherent object candidates for feature fusion.

Third, the Semantic Fusion Modulator (SFM) integrates and enhances multimodal features through two submodules: the Mask-Saliency Adapter (MSA), which generates soft saliency maps, and the Feature Enhancement Recomposer (FER), which adaptively modulates the RGB features. These modules jointly direct attention toward semantically meaningful and structurally consistent regions.

Finally, the object detection head adopts the standard YOLOv12 architecture, ensuring inference efficiency while leveraging the enriched feature representations guided by the Attention-Aware Weight Estimator (AWE).

The complete pipeline is formally defined as:


$$\begin{array}{c} \hat{y}=\text{AWE}(\text{YOLO}(F(I,M)),\text{ YOLO}(I)),\text{ s}.\text{t}.\;M\\ =\text{SPGM}(I,\text{CLIP}(I,C)),\;F(I,M)=\text{SFM}(I,M). \end{array}$$

where *I* denotes the input image, *C* represents the textual label or class name, *M* is the segmentation mask generated by the Semantic Prior Generation Module (SPGM) under CLIP guidance, *F*(·) denotes the Semantic Fusion Modulator (SFM, i.e., MSA + FER), and $\hat{y}$ represents the final detection result adaptively fused by the Attention-Aware Weight Estimator (AWE).

### 2.2 Multimodal prompt-guided segmentation with CLIP and SAM

To facilitate controllable and class-specific semantic localization, we introduce a CLIP-based prompting strategy that links textual priors with visual segmentation. This component establishes a direct association between category semantics and spatial attention, allowing the model to generate focused and semantically aligned masks.

**Figure 3:**
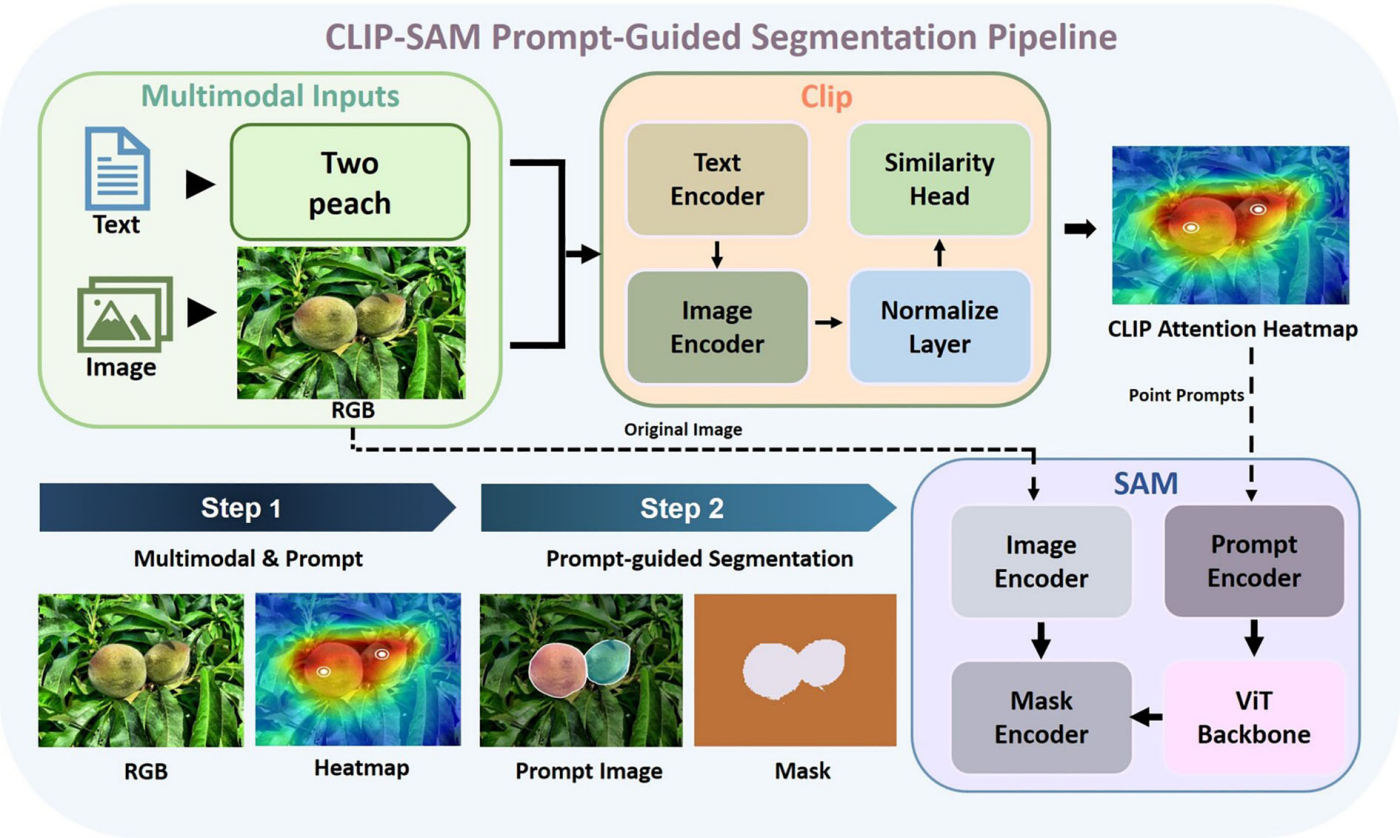
CLIP-SAM prompt-guided segmentation pipeline.

As illustrated in **Figure 3**, the prompting procedure proceeds as follows:

Given an input image *I* ∈ ℝ^3×^*^H^*^×^*^W^* and a textual query *t* (e.g. “a ripe peach”), the framework performs a multi-stage process:

Specifically, the textual query *t* is first encoded into an embedding *z_t_* via the CLIP text encoder. The input image *I* is divided into spatial tiles {*x_i,j_*}, each of which is processed through the CLIP image encoder to obtain visual embeddings *z_i,j_*. Cosine similarities *s_i,j_* are computed between each visual embedding and the text embedding, yielding a dense attention map. This map is then normalized to produce A, a spatially aligned semantic heatmap. A heuristic selection function P identifies high-activation points *p*, which serve as semantic prompts. These prompts, combined with the original image *I*, are fed into the SAM module to generate segmentation masks *M* that reflect both structural boundaries and semantic intent. The detailed method is explained in **Algorithm 1**.

Algorithm 1 **Prompt guided mask generation via CLIP and SAM.**

**Input:** Text query *t*, image *I =* {*x_i,j_*}
**Output:** Segmentation mask *M*
1 *z_t_*← CLIP_text_(*t*); //Encode the text query
2 **foreach***patch x_i,j_ in I***do**
3       *z_i,j_* ← CLIP_image_(*x_i,j_*); //Encode image patch
4       *s_i,j_* ← >*z_t_,z_i,j_*< //Compute similarity
5 *A* ← Normalize({*s_i,j_*}); //Normalize similarity map
6 *p* ← (*A*); //Generate spatial prompts from attention
7 *M* ← SAM(*I,p*); //Generate mask using SAM
8 **return***M*

This text-to-mask prompting mechanism enhances the controllability of the segmentation process and provides reliable structural priors for subsequent multimodal fusion. In contrast to traditional region proposal methods, it can dynamically associates category semantics with spatial locations.

### 2.3 Mask-Saliency Adapter

**Figure 4:**
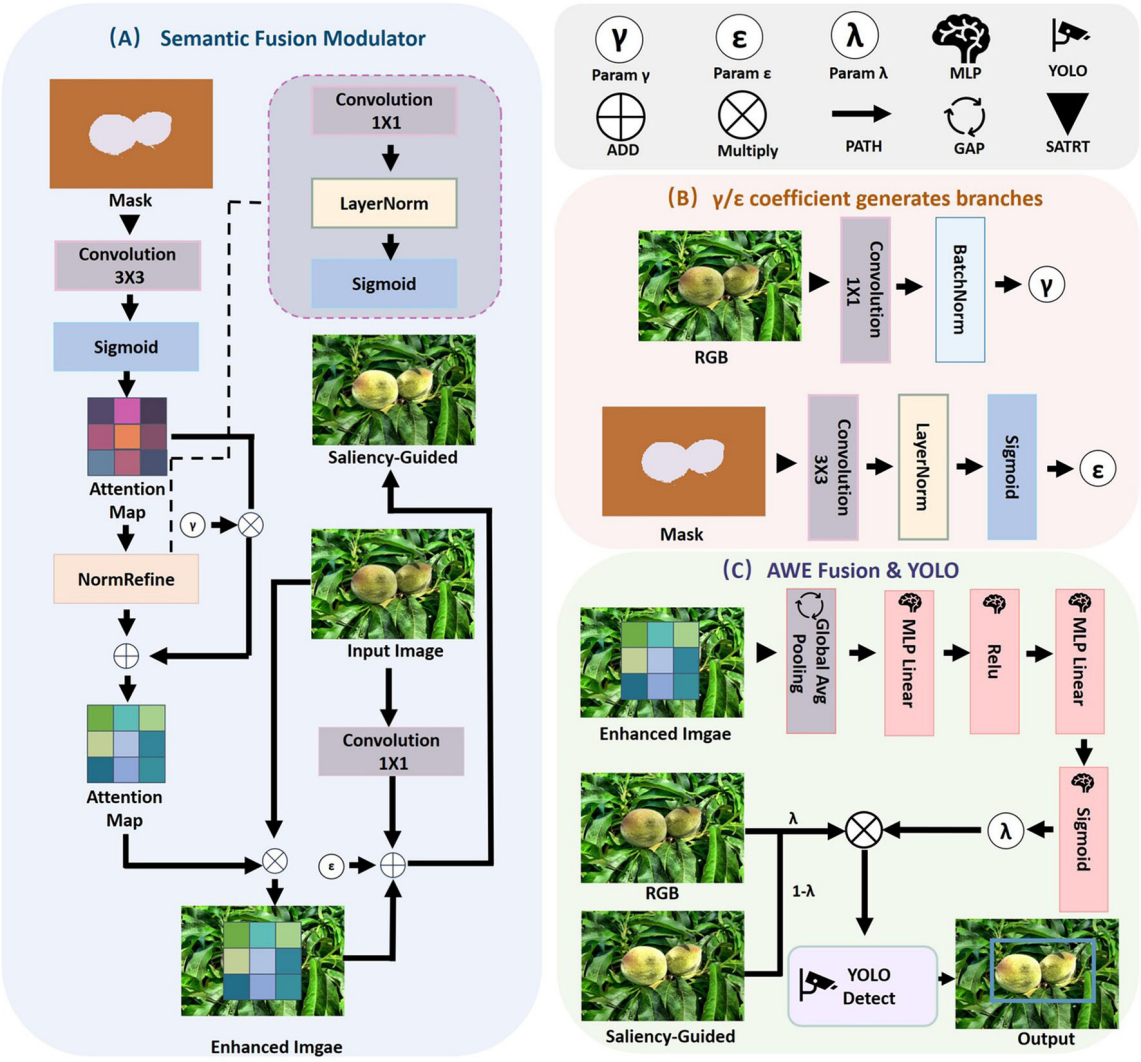
Overview of the multimodal fusion and detection framework. **(A)** Semantic Fusion Modulator (SFM), which enhances RGB features using soft saliency maps and mask-guided modulation via dual attention paths; **(B)** coefficient generation branches that compute semantic-aware modulation parameters γ and ϵ from RGB and mask inputs through lightweight convolutional blocks; and **(C)** Attention-Aware Weight Estimator (AWE), which aggregates enhanced and original features via global saliency-aware pooling and MLP-driven fusion, producing final detection results through the YOLOv12 head.

The proposed Mask-Saliency Adapter (MSA) is designed to extract and refine saliency cues from class-agnostic masks, as illustrated in part A of **Figure 4**, which are subsequently utilized to guide spatial attention within the fusion process. Instead of directly modifying the RGB feature space, MSA constructs a sparse and stable attention map from the input mask ℳ ∈ ℝ*^H^*^×^*^W^*, emphasizing semantically relevant regions with improved consistency.

The raw mask ℳ is first processed by a compact convolutional block C_3_ followed by a non-linear activation function *ϕ*, yielding the initial saliency response map S:

(1) $$S=\phi (C_{3}(M)),\text{ with }\phi =\text{Sigmoid}$$

To promote spatial smoothness and reduce numerical variance, S is refined using a lightweight normalization module *N*_ref_, consisting of a point-wise convolution C_1_, followed by layer normalization and a ReLU activation:

(2) $$S^{\prime }=\text{ReLU}(\text{LayerNorm}(C_{1}(S)))$$

The layer normalization applied to $S^{\prime }\in {\mathbb{R}}^{H\times W}$ is defined as:

(3) $${\hat{S}}_{i,j}=\frac{S_{i,j}^{'}-\mu }{\sqrt{\sigma^{2}+\epsilon }},\;\mu =\frac{1}{HW}\sum_{i,j}S_{i,j}^{'},\;\sigma^{2}=\frac{1}{HW}\sum_{i,j}{\left(S_{i,j}^{'}-\mu \right)}^{2}$$

The resulting normalized attention map $\hat{S}$ captures object-centric spatial structures and acts as a saliency driven prior in the fusion process, supporting semantically aware modulation without directly altering the feature space at this stage.

### 2.4 Feature Enhancement Recomposer

To enhance semantic expressiveness while preserving contextual integrity, we propose a dual-branch module termed Feature Enhancement Recomposer (FER). As shown in Part A of **Figure 4**, FER incorporates a saliency-guided enhancement pathway and a residual compensation pathway to enable fine-grained feature modulation with stabilized dynamics.

Saliency-Guided Pathway (Part B): The refined attention map $\hat{S}\in {\mathbb{R}}^{H\times W}$ is passed through a point-wise convolution $C_{1}$, followed by layer normalization and a sigmoid activation to produce the spatial modulation coefficient *δ*:

(4) $$\delta =\text{Sigmoid}(\text{LayerNorm}(C_{1}(\hat{S})))$$

This coefficient adjusts the spatial emphasis adaptively. The input feature map $I\in {\mathbb{R}}^{3\times H\times W}\;$ is modulated as:

(5) $$F_{\text{mod}}=I⚬(1+\delta \;*\;\hat{S})$$

where ⚬ denotes element-wise multiplication and ∗ represents broadcasted spatial fusion.

Residual Compensation Pathway (Part B): To preserve structural consistency and suppress possible amplification artifacts, the input features are also processed through a parallel residual branch:

(6) $$R=\text{BatchNorm}(C_{1}(I))$$

Final Fusion: The outputs from both branches are combined to produce the FER-enhanced feature map:

(7) $$F_{\text{fer}}=F_{\text{mod}}+R$$

This design enables targeted spatial enhancement while preventing over-amplification or feature degradation, making it suitable for few-shot scenarios with limited annotations. Additionally, FER remains lightweight and model-agnostic, ensuring compatibility with mainstream detection architectures and supporting efficient deployment.

### 2.5 Detection Integration with Attention-Aware Weight Estimator

To validate the fused visual-semantic representations, the final feature map $F_{\text{fer}}$ is forwarded to the original YOLOv12 architecture, retaining its backbone, neck, and head modules to preserve inference efficiency and deployment compatibility, as shown in Part C of **Figure 4**.

To further enhance decision reliability under few-shot conditions, we introduce an adaptive fusion mechanism termed Attention-Aware Weight Estimator (AWE), which dynamically assigns fusion weights based on global attention cues rather than fixed heuristics or confidence thresholds.

Specifically, the refined attention map $\tilde{A}\in {\mathbb{R}}^{H\times W}$, obtained from the preceding Mask-Saliency Adapter (MSA), is subjected to global average pooling to extract a scalar descriptor:

(8) $$z=\text{GAP}(\tilde{A})\in {\mathbb{R}}^{1}$$

This descriptor is passed through a lightweight MLP regressor to generate a soft fusion weight *λ*:

(9) $$\lambda =\sigma (W_{2}\cdot \text{ReLU}(W_{1}\cdot z+b_{1})+b_{2})$$

where **W**_1_, **W**_2_ are trainable weights, **b**_1_, **b**_2_ are biases, and *σ*(·) denotes the sigmoid activation function, ensuring *λ* ∈ (0, 1).

The final prediction is obtained by a weighted integration of semantic-enhanced and original RGB-based detections:

(10) $$\hat{y}=\lambda \cdot \text{YOLO}(F_{\text{fer}})+(1-\lambda )\cdot \text{YOLO}(I)$$

This adaptive blending enables the model to adjust modality dependence in a data-driven manner, improving robustness against occlusion, background clutter, and scale variation. The AWE module imposes minimal computational overhead while significantly contributing to decision stability and generalization performance.

To summarize, the proposed framework establishes a lightweight yet expressive detection pipeline by combining CLIP-guided prompt generation, class-agnostic segmentation priors (SAM), semantic-aware fusion modules (MSA and FER), and attention-weighted feature blending (AWE). Specifically, CLIP serves as a visual-language encoder to generate text-driven point prompts, allowing SAM to extract semantically aligned object masks and suppress irrelevant background noise. These refined masks are subsequently fused with RGB features through the MSA and FER modules, which enhance saliency focus and preserve contextual integrity. Finally, the AWE module dynamically balances the contributions of original and enhanced features based on global attention cues. The overall architecture maintains plug-and-play compatibility with YOLOv12 and demonstrates strong generalization ability under few-shot learning constraints. Its modularity and low complexity make it suitable for real-world agricultural monitoring applications, especially in scenarios involving rare crop varieties or region-specific fruit species.

## 3 Experiments and results

### 3.1 Dataset preparation

To validate the effectiveness of our tri-modal few-shot detection framework in agricultural biodiversity recognition, we curated four representative fruit datasets—each capturing a distinct cultivar with high visual similarity, occlusion, and phenotype variance under field conditions:

- *Cantaloupe.v2* contains 220 images of greenhouse-grown cantaloupes exhibiting complex netted surface textures and frequent occlusion by leaves or support structures. It is divided into 152 training, 45 validation, and 23 testing samples. The dataset emphasizes morphological complexity and background interference.
- *Peach.v3* consists of 209 images across different ripening stages and varieties, including yellow-flesh and red-blush cultivars. With 165 samples for training, 30 for validation, and 14 for testing, this dataset highlights challenges in shape similarity, gloss variation, and clustered instances.
- *Watermelon.v2* includes 172 images of both early-stage and mature watermelons, captured under natural illumination and soil-rich conditions. With 140 training, 21 validation, and 11 testing samples, it offers a realistic setting for evaluating detection under occlusion, soil-background confusion, and growth-stage variability.
- *Orange.v8* features 165 orchard images of orange cultivars, captured under varied lighting and foliage occlusion. It is split into 120 training, 29 validation, and 16 testing samples. The dataset is ideal for assessing fine-grained classification under dense canopy and color similarity conditions.

Together, these four datasets constitute a well-structured benchmark for evaluating few-shot object detection in real-world orchard scenarios. They encompass critical agricultural challenges, including cultivar ambiguity, scale inconsistencies, occlusion, and annotation sparsity. As such, they are well aligned with the objectives of biodiversity surveillance and the construction of digital orchard archives. Furthermore, to verify the framework’s cross-domain generalization capability beyond our self-built datasets, additional experiments were conducted on public fruit subsets (Apple and Durian) derived from the Kaggle Fruits & Vegetable Detection dataset, as presented in **Appendix**. A schematic overview of the dataset construction, splitting, and augmentation process is illustrated in **Appendix Figure 1** for improved clarity.

### 3.2 Training settings

All training procedures are implemented using PyTorch 1.12.0 and executed on a single NVIDIA RTX 3090 GPU. The detector adopts YOLOv12 as the backbone, with the lightweight configuration modelsize n to ensure computational efficiency. Optimization is performed using the Adam algorithm with an initial learning rate of 0.001, scheduled by cosine annealing. To mitigate overfitting, a weight decay of 5 × 10^−4^ is applied alongside early stopping with a patience of 100 epochs. Each training session runs for up to 100 epochs with a batch size of 16, and model checkpoints are saved every 5 epochs. A fixed random seed of 42 is used for reproducibility.

For multimodal components, the Segment Anything Model (SAM) is initialized with the pre-trained checkpoint saml, providing class-agnostic structural priors. The language-guided module employs DFN5B-CLIP-ViT-H-14-378, a high-resolution Vision Transformer variant tailored for domain-specific prompt encoding. To fully utilize the introduced tri-modal features, we regenerate the training, validation, and test splits with updated augmented samples, ensuring better adaptation of YOLOv12 to semantic and structural cues.

To address the limited generalization capacity in few-shot learning, we construct a hybrid augmentation pipeline that introduces both distributional diversity and structural variation. Color perturbations include HSV jittering with parameters hsv*_h_* = 0.015, hsv*_s_* = 0.8, and hsv*_v_* = 0.4. Geometric transformations involve translation (0.2), scaling (up to 0.5), rotation (± 10^°^), and perspective distortion (0.001). Structure-aware augmentations are applied during training, including Mosaic (probability 1.0), MixUp (0.3), and Copy-Paste (0.4). Random horizontal and vertical flips are applied with probabilities of 0.5 and 0.2, respectively.

This configuration is designed to preserve fine-grained structural cues while promoting cross-modal feature alignment, enabling stable convergence under sparsely annotated conditions.

### 3.3 Evaluation settings and comparative baselines

To comprehensively evaluate the effectiveness of the proposed semantic-guided fusion framework, we conduct comparative experiments against several representative few-shot object detection (FSOD) baselines, as well as ablation studies within our own architecture. The comparative evaluation includes five established approaches: YOLOv12 (vanilla), which serves as a strong single-stage baseline without multimodal integration; DETR, a transformer-based detector that performs end-to-end detection using object queries; TFA-WO-FPN, a meta-learning method based on fine-tuning without the feature pyramid network; Meta R-CNN, which incorporates attention-driven feature reweighting for class-specific adaptation; and DeFRCN, which employs decoupled feature refinement and prototype learning to improve generalization.

All models are trained and evaluated on four benchmark datasets: *Cantaloupe.v2*, *Peach.v3*, *Watermelon.v2*, and *Orange.v8*. A 5-fold cross-validation strategy is adopted for each dataset, and the final performance is reported as the average across all folds to mitigate bias introduced by sample partitioning. Evaluation is conducted using standard detection metrics, including AP@0.5, AP@0.75, AP@[.50:.95], Precision, and Recall@all, providing a comprehensive assessment of localization accuracy and recall behavior.

In addition to the baseline comparisons, an ablation study is performed to isolate the contributions of key components in the proposed framework. The configurations include: the baseline YOLOv12 model without semantic priors; YOLOv12 enhanced with the Semantic Fusion Modulator (SFM), which combines the Mask-Saliency Adapter (MSA) and Feature Enhancement Recomposer (FER) for multimodal representation refinement; and the complete model with SFM and the Attention-Aware Weight Estimator (AWE), which adaptively balances semantic-enhanced and original RGB features.

To further assess the effectiveness of semantic guidance, we additionally compare SAM-based segmentation results under two conditions: with and without CLIP-derived point prompts. Overall, incorporating CLIP enables SAM to focus on category-relevant regions, while the absence of semantic prompting often leads to the segmentation of irrelevant or excessive background regions, which may interfere with downstream detection.

Building on this insight, we further examine the influence of semantic modules on the detection backbone by employing Grad-CAM to visualize class-specific saliency maps under different architectural settings. The resulting visualizations reveal that the inclusion of prompt-based modules (CLIP), structure-aware priors (SAM), fusion refinement (SFM), and adaptive weighting (AWE) leads to more focused and consistent attention distributions, particularly under challenging orchard conditions such as leaf occlusion, cultivar overlap, and complex backgrounds.

### 3.4 Comparative evaluation

**Figure 5:**
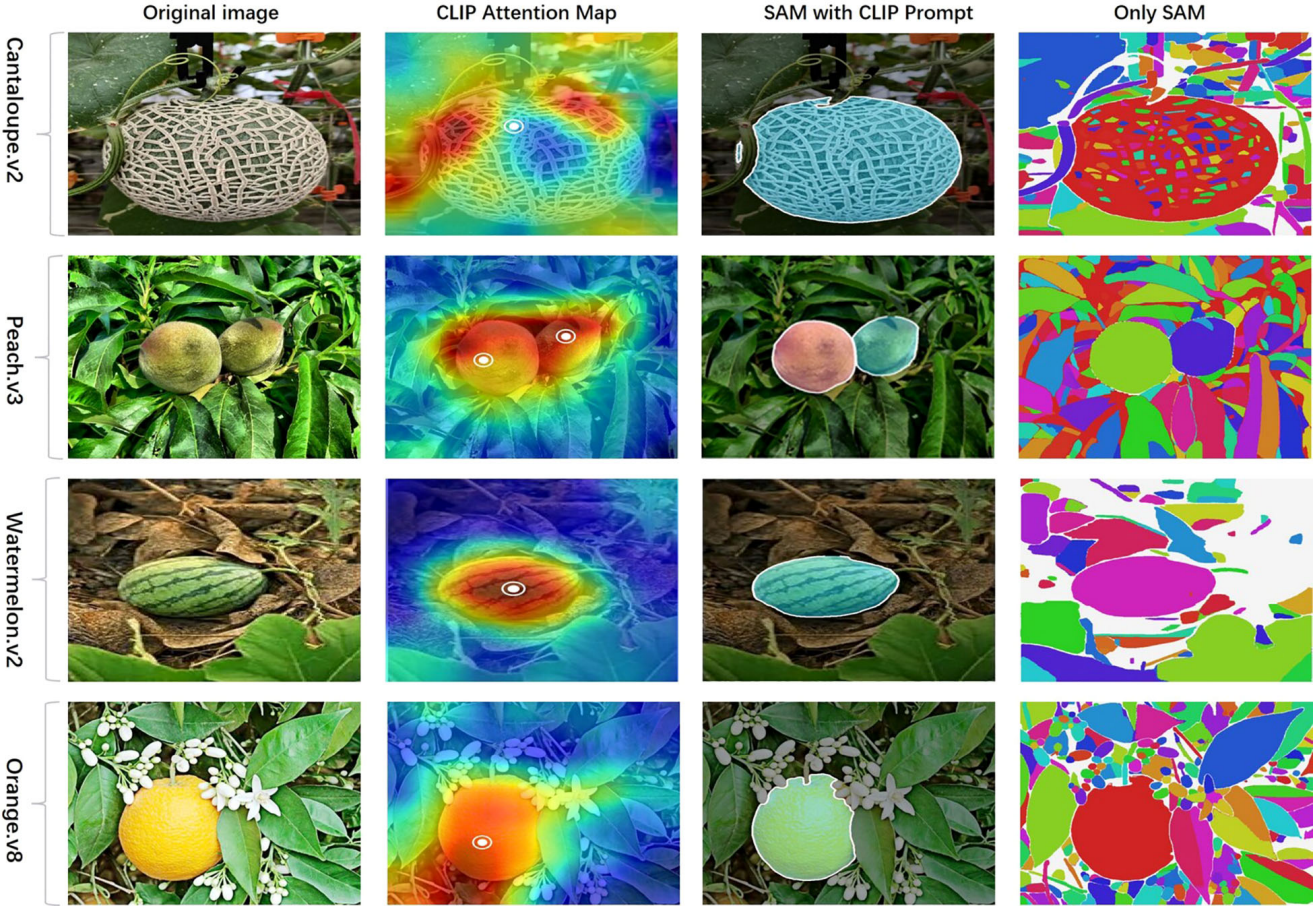
Segmentation comparison between CLIP-guided and standalone SAM. Comparison between CLIP-guided SAM and standalone SAM across four fruit categories: Cantaloupe. v2, Peach. v3, Watermelon. v2, and Orange.v8. Each row shows an original orchard image (left), followed by the CLIP attention heatmap with high-activation points (2nd column), the segmentation results generated by SAM with CLIP prompts (3rd column), and the standalone SAM output without guidance (right). The CLIP-guided SAM consistently produces cleaner and more semantically aligned masks, while standalone SAM often results in over-segmentation or object fragmentation due to lack of category-specific guidance.

During the segmentation stage, we provide a visual comparison of detection-relevant mask quality across four representative fruit categories. As illustrated in the **Figure 5**, each row corresponds to a specific species (cantaloupe, peach, watermelon, orange), while the columns show the RGB input, SAM guided by CLIP point prompts, and unguided SAM outputs. It is evident that CLIP-conditioned prompting enables SAM to concentrate on target fruit regions, generating compact and semantically coherent masks. In contrast, the unguided SAM often produces over-segmented masks, frequently capturing irrelevant background textures or non-target regions. These observations support the hypothesis that CLIP-derived prompts function as semantic filters, guiding the segmentation process toward task-specific content and thereby improving the quality of downstream detection. To further substantiate the effectiveness of the CLIP-guided SAM, we conducted additional comparative experiments with several state-of-the-art segmentation models. The detailed results and visual analyses are presented in **Appendix**.

To evaluate the performance of the proposed YOLOv12 (CLIP+SAM + SFM + AWE) framework in few-shot object detection (FSOD), we conduct a systematic comparison on four benchmark datasets: *Cantaloupe.v2*, *Peach.v3*, *Watermelon.v2*, and *Orange.v8*. Competing methods include YOLOv12 (Tian et al., 2025) as a single-stage baseline, DETR (Carion et al., 2020) as a transformer-based end-to-end detector, TFA-WO-FPN (Wang et al., 2020), Meta R-CNN (Yan et al., 2019), and DeFRCN (Qiao et al., 2021), which collectively represent dominant approaches in the FSOD literature. Evaluation is performed using standard detection metrics: AP@0.5, AP@0.75, AP@[.50:.95], Precision, and Recall@all. The detailed comparison results are reported in **Table 1**.

**Table 1:** Detection performance of different methods on four fruit datasets.

Dataset CANTALOUPE	AP@0.5	AP@0.75	AP@[.50:.95]	Precision	Recall@all
Ours	**0.9403**	**0.7694**	**0.6342**	**0.8712**	**0.7916**
YOLOv12	0.8611	0.6916	0.5652	0.7953	0.7094
DETR	0.7271	0.5760	0.4521	0.6701	0.5902
TFA-WO-FPN	0.7661	0.6213	0.4980	0.7129	0.6266
Meta R-CNN	0.7997	0.6465	0.5279	0.7277	0.6671
DeFRCN	0.8127	0.6570	0.5468	0.7446	0.6858

Dataset PEACH	AP@0.5	AP@0.75	AP@[.50:.95]	Precision	Recall@all
Ours	**0.9298**	**0.6862**	**0.5715**	**0.8660**	**0.7342**
YOLOv12	0.8723	0.6279	0.5192	0.8117	0.6725
DETR	0.7058	0.4742	0.3779	0.6348	0.5227
TFA-WO-FPN	0.7685	0.5427	0.4443	0.6851	0.5849
Meta R-CNN	0.7931	0.5698	0.4685	0.7094	0.6112
DeFRCN	0.8184	0.5935	0.4927	0.7320	0.6371

Dataset WATERMELON	AP@0.5	AP@0.75	AP@[.50:.95]	Precision	Recall@all
Ours	**0.9405**	**0.6742**	**0.5594**	**0.8756**	**0.7561**
YOLOv12	0.8589	0.5992	0.4907	0.8012	0.6784
DETR	0.7299	0.5014	0.4044	0.7055	0.5642
TFA-WO-FPN	0.7561	0.5343	0.4177	0.6925	0.6160
Meta R-CNN	0.7919	0.5648	0.4674	0.7296	0.6195
DeFRCN	0.8251	0.5804	0.4685	0.7831	0.6643

Dataset ORANGE	AP@0.5	AP@0.75	AP@[.50:.95]	Precision	Recall@all
Ours	**0.8739**	**0.7114**	**0.5625**	**0.8627**	**0.7762**
YOLOv12	0.8214	0.6527	0.5096	0.8074	0.7128
DETR	0.6782	0.4894	0.3657	0.6255	0.5186
TFA-WO-FPN	0.7451	0.5386	0.4213	0.6823	0.5827
Meta R-CNN	0.7623	0.5661	0.4498	0.7056	0.6093
DeFRCN	0.7887	0.5920	0.4726	0.7298	0.6375

Bold values denote the highest score in each metric for each dataset.

On the Cantaloupe.v2 dataset (see **Table 1**, CANTALOUPE section), the proposed model consistently surpasses all baselines across evaluation metrics. Specifically, it achieves an AP@0.5 of 0.9403, outperforming YOLOv12 and Meta R-CNN by 9.21% and 17.56%, respectively. The AP@[.50:.95] rises to 0.6342, reflecting a 12.19% gain over YOLOv12 and 10.61% over DeFRCN. At the stricter AP@0.75 threshold, our model reaches 0.7694, exceeding YOLOv12 and Meta R-CNN by 11.25% and 12.30%, respectively. Further improvements are observed in Precision (+7.59%) and Recall@all (+8.22%), demonstrating the model’s effectiveness in producing precise predictions under cluttered orchard conditions.

On the Peach.v3 dataset, the model obtains an AP@0.5 of 0.9298, outperforming YOLOv12 by 6.59% and DETR by 31.66%. The AP@[.50:.95] increases to 0.5715, which is 10.07% higher than YOLOv12 and 19.36% higher than Meta R-CNN. At AP@0.75, the model achieves 0.6862, exceeding YOLOv12 and Meta R-CNN by 9.26% and 11.63%, respectively. Precision and Recall@all also improve by 5.34% and 6.17%, respectively, indicating enhanced segmentation precision and better handling of cultivar shape variations.

On the Watermelon.v2 dataset, which includes large shape variation and irregular lighting, our method attains an AP@0.5 of 0.9405, outperforming YOLOv12 by 9.51% and DETR by 28.82%. The AP@[.50:.95] climbs to 0.5594, showing a 13.97% gain over YOLOv12 and 15.5% over Meta R-CNN. The model also shows consistent improvement in AP@0.75 (+12.52% over YOLOv12), Precision (+7.42%), and Recall@all (+7.77%), confirming its robustness under diverse environmental factors.

On the Orange.v8 dataset, our model yields an AP@0.5 of 0.8739, surpassing YOLOv12 and DETR by 6.40% and 28.86%, respectively. The AP@[.50:.95] improves to 0.5625, which is 10.39% higher than YOLOv12 and 11.27% higher than TFA-WO-FPN. Gains are also observed in AP@0.75 (+8.98% over YOLOv12), Precision (+5.53%), and Recall@all (+6.34%), reinforcing the model’s capability to adapt to occlusion, lighting variability, and similar inter-class appearance in field imagery.

**Figure 6:**
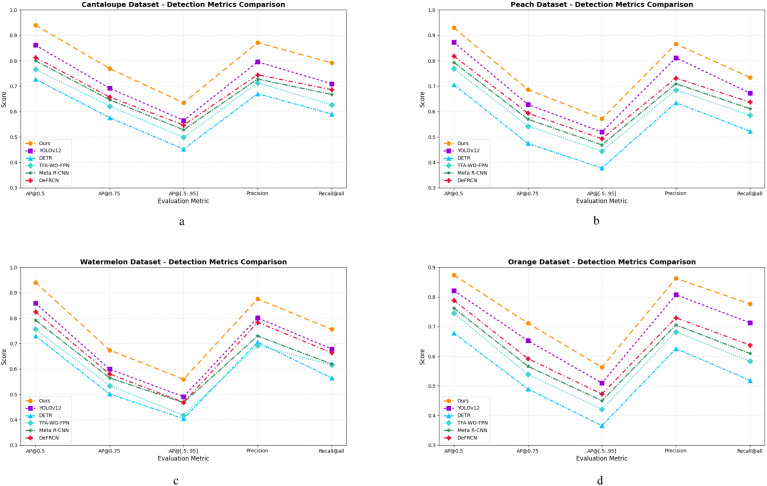
Comparison of detection metrics on four fruit datasets: **(a)** Cantaloupe.v2, **(b)** Peach.v3, **(c)** Watermelon.v2, and **(d)** Orange.v8. Results are reported for six methods over five standard metrics (AP@0.5, AP@0.75, AP@[.5:.95], Precision, Recall@all). Our method consistently outperforms existing FSOD baselines, particularly under stricter thresholds (e.g. AP@0.75) and in terms of precision and recall, indicating stronger localization accuracy and robustness in few-shot agricultural scenarios.

To further substantiate the superiority of the proposed method across multiple evaluation metrics, five representative detection metrics—including AP@0.5, AP@0.75, AP@[.50:.95], Precision, and Recall@all—are visualized through line plots in **Figure 6**. These plots illustrate performance trends across all four fruit benchmark datasets, with **Figures 6a–d** corresponding to *Cantaloupe.v2*, *Peach.v3*, *Watermelon.v2*, and *Orange.v8*, respectively.

These plots provide an intuitive and comparative overview of the detection performance achieved by the proposed YOLOv12 (CLIP + SAM + SFM + AWE) framework across four representative fruit datasets: *Cantaloupe.v2*, *Peach.v3*, *Watermelon.v2*, and *Orange.v8*. Compared to five state-of-the-art FSOD baselines, the proposed method consistently achieves superior results, particularly in AP@[.50:.95] and Recall@all—two critical indicators for evaluating generalization ability and localization robustness under limited supervision. Notably, the performance margin becomes more evident in visually complex scenarios, such as inter-class similarity (*Peach*, *Orange*) and large intra-class variation due to maturity stages and lighting conditions (*Watermelon*, *Cantaloupe*). These visual comparisons further validate the quantitative results reported in **Table 1**, underscoring the effectiveness of our framework for fine-grained fruit cultivar recognition and digital orchard archiving in resource-constrained agricultural settings.

**Figure 7:**
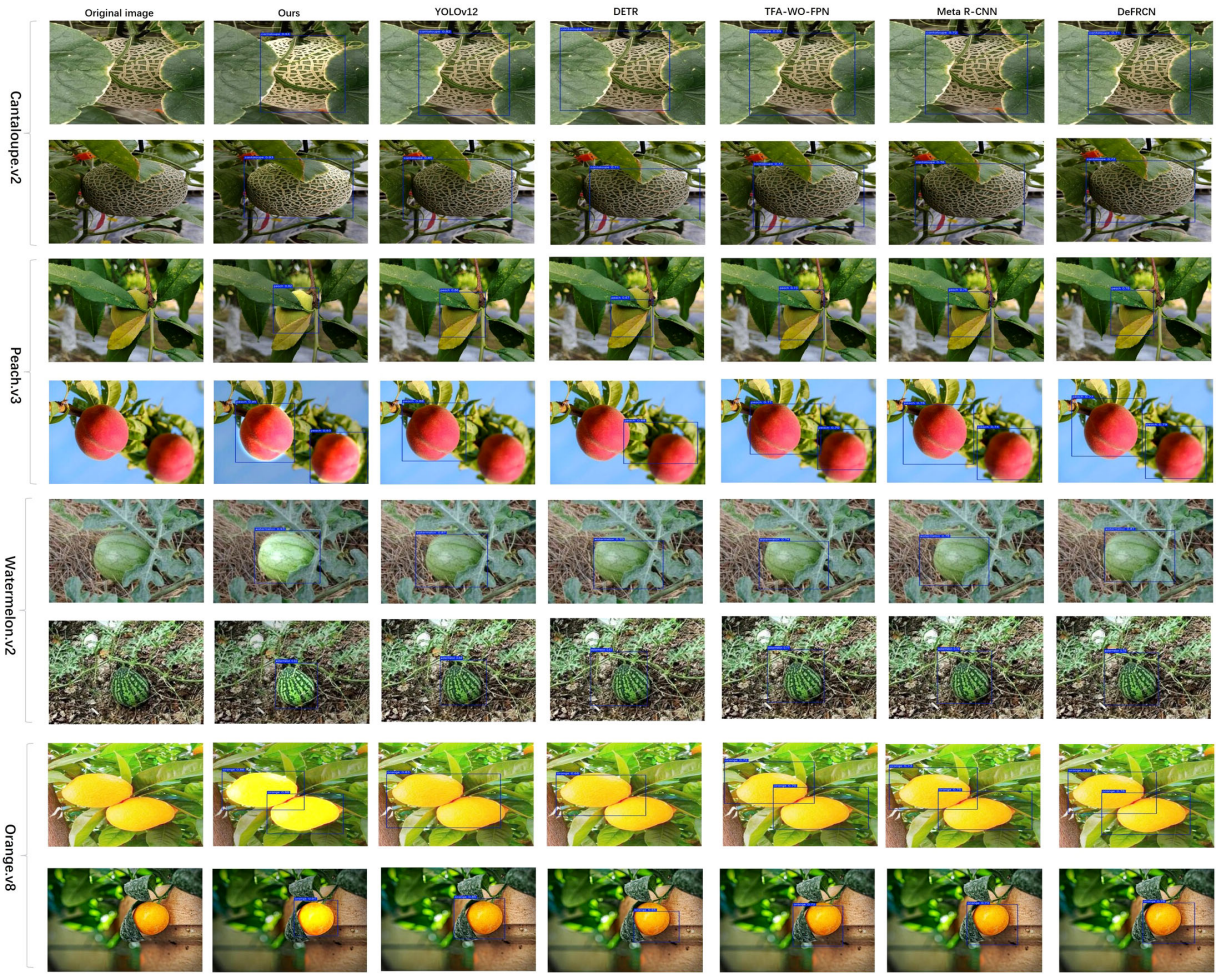
Qualitative detection comparisons on Cantaloupe, Peach, Watermelon, Orange datasets. Each group shows two test images with predictions from six methods (Ours, YOLOv12, DETR, TFA-WO-FPN, Meta R-CNN, DeFRCN), alongside the original image. Our model produces more accurate and complete bounding boxes, especially under occlusion, clutter, or close-proximity instances.

In addition to quantitative evaluations, qualitative comparisons are conducted on representative test samples from all four fruit datasets, as illustrated in **Figure 7**. The visual results indicate that the proposed YOLOv12 (CLIP + SAM + SFM + AWE) model consistently delivers more accurate and complete detections compared to baseline FSOD approaches, especially under real-world agricultural challenges such as partial occlusion, background interference, inter-varietal similarity, and diverse maturity stages.

On the *Cantaloupe.v2* dataset, our model generates compact and precise bounding boxes even when fruits are partially covered by leaves, netting, or overlapping samples, whereas methods like DETR and Meta R-CNN tend to produce redundant or incomplete detections. For the *Peach.v3* dataset, which involves cultivars with highly similar visual traits, our approach effectively distinguishes subtle shape and color differences, resulting in fewer false positives and improved consistency across instances.

On the *Watermelon.v2* dataset, which features significant variation in rind patterns and lighting conditions, the proposed framework maintains robust detection by adaptively attending to structure-guided cues while suppressing irrelevant background signals. Similarly, on the *Orange.v8* dataset, the model exhibits enhanced localization in cluttered orchard scenes, accurately identifying densely arranged or partially shaded oranges with superior boundary alignment and minimal confusion among overlapping samples.

These visual comparisons reinforce the quantitative findings summarized in **Table 1**, highlighting the effectiveness of our semantic-guided multimodal fusion strategy in enhancing fruit cultivar identification, supporting fine-grained phenotyping, and facilitating species-level digital archiving in orchard environments under few-shot constraints.

### 3.5 Ablation study

To evaluate the contribution of each module in the proposed framework, we conduct a series of ablation studies by incrementally integrating the designed components into the YOLOv12 baseline. As shown in **Table 2**, incorporating the Mask-Saliency Adapter (MSA) results in a noticeable performance improvement by introducing soft attention cues that guide spatial focus during detection. Adding the Feature Enhancement Recomposer (FER) further enhances accuracy by adaptively modulating the original RGB features with saliency-aware residuals.

**Table 2:** Ablation study on four fruit datasets.

Dataset CANTALOUPE	AP@0.5	AP@0.75	AP@[.5:.95]	Precision	Recall@all
Baseline	0.8611	0.6916	0.5652	0.7953	0.7094
Baseline + MSA	0.8686	0.7004	0.5738	0.8054	0.7144
Baseline + MSA + FER	0.9042	0.7358	0.5997	0.8351	0.7492
Baseline + MSA + FER + CLIP	0.9321	0.7601	0.6254	0.8609	0.7821
Baseline + MSA + FER + CLIP + AWE	**0.9403**	**0.7694**	**0.6342**	**0.8712**	**0.7916**

Dataset PEACH	AP@0.5	AP@0.75	AP@[.5:.95]	Precision	Recall@all
Baseline	0.8723	0.6279	0.5192	0.8117	0.6725
Baseline + MSA	0.8802	0.6349	0.5264	0.8192	0.6789
Baseline + MSA + FER	0.9128	0.6611	0.5460	0.8395	0.7091
Baseline + MSA + FER + CLIP	0.9269	0.6780	0.5660	0.8581	0.7262
Baseline + MSA + FER + CLIP + AWE	**0.9298**	**0.6862**	**0.5715**	**0.8660**	**0.7342**

Dataset WATERMELON	AP@0.5	AP@0.75	AP@[.5:.95]	Precision	Recall@all
Baseline	0.8589	0.5992	0.4907	0.8012	0.6784
Baseline + MSA	0.8670	0.6072	0.4998	0.8092	0.6837
Baseline + MSA + FER	0.9054	0.6390	0.5273	0.8445	0.7160
Baseline + MSA + FER + CLIP	0.9341	0.6660	0.5522	0.8682	0.7477
Baseline + MSA + FER + CLIP + AWE	**0.9405**	**0.6742**	**0.5594**	**0.8756**	**0.7561**

Dataset ORANGE	AP@0.5	AP@0.75	AP@[.5:.95]	Precision	Recall@all
Baseline	0.8214	0.6527	0.5096	0.8074	0.7128
Baseline + MSA	0.8269	0.6588	0.5168	0.8141	0.7186
Baseline + MSA + FER	0.8514	0.6831	0.5388	0.8383	0.7450
Baseline + MSA + FER + CLIP	0.8672	0.7039	0.5540	0.8561	0.7663
Baseline + MSA + FER + CLIP + AWE	**0.8739**	**0.7114**	**0.5625**	**0.8627**	**0.7762**

Bold values denote the highest score in each metric for each dataset.

The integration of CLIP-based prompt guidance introduces semantic priors that improve region localization, particularly in cases involving occlusion or visual ambiguity. Finally, the addition of the Attention-Aware Weight Estimator (AWE) enables dynamic feature reweighting between the semantic-enhanced and original pathways, achieving the highest detection performance across all evaluated datasets. These cumulative improvements confirm the complementary nature of the proposed modules and underscore the effectiveness of the tri-modal fusion strategy.

On the *Cantaloupe* dataset, introducing MSA improves AP@0.5 from 0.8611 to 0.8686 and AP@[.50:.95] from 0.5652 to 0.5738, indicating enhanced focus on salient object regions. With FER added, AP@0.5 and AP@0.75 rise to 0.9042 and 0.7358, respectively. Further gains are observed when CLIP guidance is introduced, lifting AP@[.50:.95] to 0.6254 and Precision to 0.8609. The full configuration with AWE achieves the highest overall performance, reaching an AP@0.5 of 0.9403 and Recall@all of 0.7916.

On the *Peach* dataset, which presents moderate intra-class variation, MSA and FER contribute to a stable increase in AP@[.50:.95] from 0.5192 to 0.546. Incorporating CLIP improves both precision (from 0.8395 to 0.8581) and recall (from 0.7091 to 0.7262). With AWE integrated, the model achieves the best results across all metrics, including an AP@0.5 of 0.9298 and an AP@0.75 of 0.6862.

In the more challenging *Watermelon* dataset—featuring significant shape variability and illumination noise—MSA and FER together increase AP@[.50:.95] from 0.4907 to 0.5273. With CLIP-enhanced semantic prompts, this metric further improves to 0.5522, while AP@0.5 climbs to 0.9341. The full model achieves an AP@0.75 of 0.6742 and Precision of 0.8756, validating its robustness in diverse conditions.

Finally, on the *Orange* dataset, the inclusion of MSA and FER yields a consistent lift in detection performance, with AP@[.50:.95] rising from 0.5096 to 0.5388. CLIP integration brings this to 0.554, and the final addition of AWE pushes it to 0.5625. Gains in Recall@all (from 0.7128 to 0.7762) further demonstrate the framework’s ability to detect subtle object variations and reduce missed detections in real-world orchard imagery.

**Figure 8:**
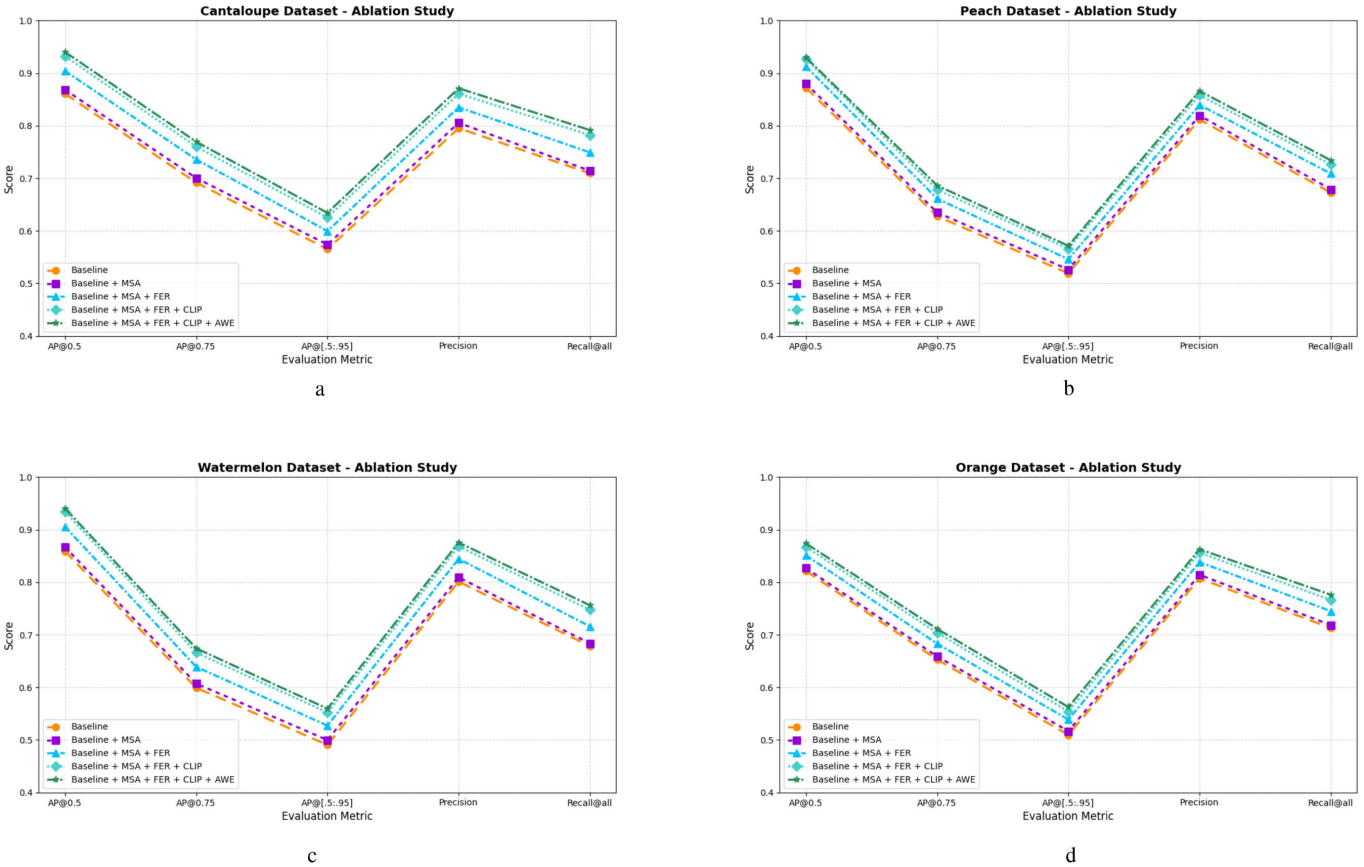
Comparison of ablation study results on **(a)** Ablation results on Cantaloupe.v2 **(b)** Ablation results on Peach.v3 **(c)** Ablation results on Watermelon.v2 **(d)** Ablation results on Orange.v8 datasets. Each subfigure shows detection performance under different module configurations, including MSA, FER, CLIP-guided semantic priors, and the AWE weighting mechanism.

To provide a clearer view of the individual contributions of each module, six key detection metrics are visualized in **Figure 8** across four benchmark datasets. **Figures 8a, d** present the ablation comparisons between the YOLOv12 baseline and its progressive variants (*+MSA*, *+MSA+FER*, *+MSA+FER+CLIP*, and *+MSA+FER+CLIP+AWE*) on *Cantaloupe.v2*, *Peach.v3*, *Watermelon.v2*, and *Orange.v8*, respectively.

Across all datasets, the integration of each module yields consistent improvements, particularly in AP@[.50:.95], Precision, and Recall@all, confirming the modular effectiveness of the semantic-guided fusion strategy. The MSA module enhances early-stage saliency by directing attention toward spatially relevant regions. FER further refines these representations through residual-based feature modulation, while the CLIP-driven semantic prior strengthens cross-modal alignment. Finally, AWE adaptively reweights features from the semantic and original pathways, resulting in the most stable and accurate detection outputs.

The upward trajectories observed across all five metrics reflect the robustness and transferability of the proposed framework, particularly under limited supervision and visually complex ecological settings. These visual results are consistent with the quantitative findings in **Table 2**, reinforcing the framework’s applicability to real-world biodiversity recognition and deployment in resource-constrained scenarios.

In addition to detection accuracy, we further evaluate the computational complexity and inference efficiency of each model configuration, as summarized in **Table 3**. Notably, both the CLIP-based prompt generator and the SAM segmentation module are used as offline preprocessing tools and do not introduce runtime overhead during inference.

**Table 3:** Model complexity, module configuration, and inference efficiency comparison.

Model	MSA	FER	AWE	Params (M)	FLOPs (G)	Inf. (ms)	FPS
Baseline	✗	✗	✗	2.57	6.51	7.6	131.6
+ MSA	✓	✗	✗	2.68	6.76	8.1	123.5
+ MSA + FER	✓	✓	✗	2.84	7.11	8.7	114.9
+ MSA + FER + AWE	✓	✓	✓	2.90	7.25	9.2	108.7

CLIP and SAM are used as offline preprocessing tools and introduce no runtime overhead.

Starting from the YOLOv12 baseline with 2.57M parameters and 6.51G FLOPs, integrating the MSA module increases the model size to 2.68M and FLOPs to 6.76G, with a modest rise in inference time to 8.1 ms. Further adding the FER module increases the parameter count to 2.84M and inference time to 8.7 ms, while maintaining reasonable FPS (114.9). Finally, incorporating AWE yields a full configuration with 2.90M parameters and 7.25G FLOPs, with inference time of 9.2 ms and FPS of 108.7.

These results demonstrate that the proposed fusion mechanism introduces only a minor computational overhead while delivering substantial performance gains. The final model remains lightweight and suitable for real-time or near-real-time deployment in resource-constrained agricultural scenarios.

### 3.6 Visual analysis of attention via Grad-CAM

**Figure 9:**
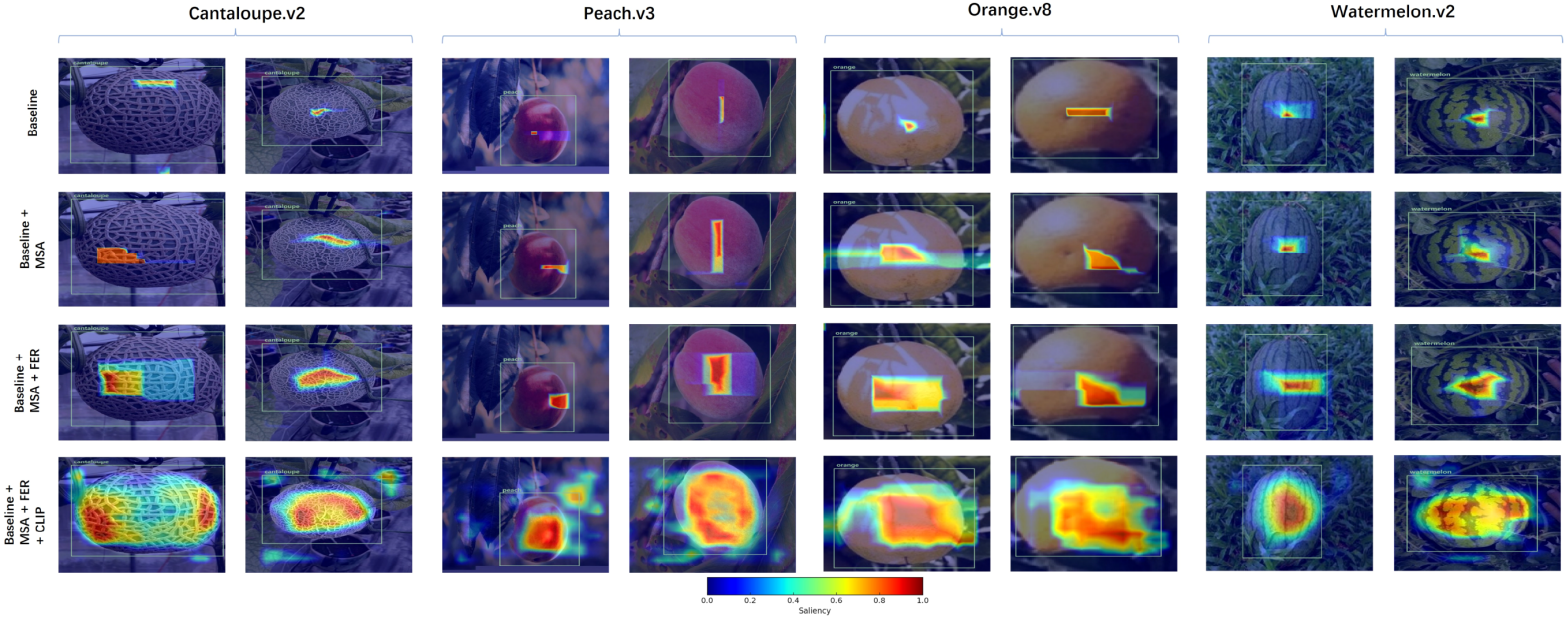
Attention heatmaps across fusion stages on four benchmark datasets. Each row represents a fusion stage, from the baseline to variants with MSA, FER, and CLIP-guided priors.

To further investigate how each fusion component influences attention distribution and spatial awareness, we employ Grad-CAM to visualize saliency maps under four ablation configurations: Baseline, Baseline + MSA, Baseline + MSA + FER, and Baseline + MSA + FER + CLIP. As shown in **Figure 9**, examples from four representative fruit datasets—*Cantaloupe.v2*, *Peach.v3*, *Watermelon.v2*, and *Orange.v8*—are selected to illustrate the evolution of attention patterns across fusion stages.

In the *Cantaloupe.v2* dataset, the baseline model tends to focus narrowly on texture regions, often ignoring the full object extent. Adding the Mask-Saliency Adapter (MSA) expands the attention area to include more of the fruit body, though the boundary remains imprecise. Incorporating the Feature Enhancement Recomposer (FER) leads to more uniform attention across the entire fruit surface, with sharper edges and better localization. Crucially, integrating the CLIP-based semantic prompts further aligns the saliency maps with the true object semantics—refining the attention distribution toward regions with distinctive category-specific traits (e.g. netted skin texture), thus improving semantic consistency and reducing irrelevant background response.

On the *Peach.v3* dataset, similar trends are observed: while the baseline struggles with shadow interference and adjacent foliage, the progressive fusion of MSA and FER improves spatial focus. The final CLIP enhanced model demonstrates strong category awareness, concentrating attention on features like peach fuzz and pit curvature—traits difficult to isolate without semantic guidance.

For the *Watermelon.v2* dataset, which contains frequent occlusions and subtle striping patterns, the baseline misplaces focus toward background grass. With MSA and FER, attention gradually shifts toward the central melon area. The addition of CLIP prompts boosts saliency on biologically meaningful attributes (e.g.rind color and radial texture), helping to suppress false positives and enabling robust detection under natural variation.

In the *Orange.v8* dataset, the baseline shows weak boundary awareness and fails to capture full object contours. MSA and FER help recover spatial completeness, while the inclusion of CLIP prompts further sharpens the attention map along object edges and surface gloss, resulting in superior interpretability and detection reliability.

The heatmap evolution provides clear evidence of how each lightweight module contributes to the observed performance gains. Specifically, the CLIP-guided semantic prior offers high-level category context that suppresses irrelevant activations and improves early semantic localization. Guided by these text-derived prompts, the SAM module produces class-agnostic masks that encode structural priors, enabling the network to capture object boundaries and spatial coherence under limited supervision. The subsequent MSA converts the masks into sparse and stable saliency maps, filtering background noise and directing attention toward structurally consistent regions. The FER module then performs residual-based feature recomposition, strengthening mid-level representations and improving boundary delineation and shape integrity. Finally, the AWE mechanism derives a soft fusion weight from globally pooled saliency cues to adaptively balance semantic-enhanced and original visual pathways, yielding more stable attention distributions and fewer misdetections in complex scenes. Together, these complementary effects explain the consistent metric improvements observed in the ablation study and the progressively more structured attention responses in **Figure 9**.

Overall, these visualizations reveal that the CLIP-driven semantic prior significantly improves the interpretability and precision of attention localization, especially in fine-grained fruit classification tasks under few-shot conditions. When combined with our lightweight MSA and FER modules, the proposed framework offers both semantic alignment and spatial fidelity, making it highly suitable for real-world agricultural species monitoring and digital archiving.

### 3.7 Summary of results

This study introduces a lightweight tri-modal fusion framework for few-shot fruit diversity recognition, specifically designed for agricultural applications such as digital orchard archiving and varietal identification. Built upon the YOLOv12 detection backbone, the framework incorporates cross-modal knowledge and spatial priors through three core components: (1) CLIP-derived semantic prompts that direct category-relevant attention; (2) SAM-generated structure-aware masks providing auxiliary spatial supervision; and (3) a Semantic Fusion Modulator (SFM), composed of the Mask-Saliency Adapter (MSA) and Feature Enhancement Recomposer (FER). Additionally, an Attention-Aware Weight Estimator (AWE) dynamically reweights semantic-enhanced and original RGB features during inference.

Experiments on four few-shot fruit datasets—*Cantaloupe.v2*, *Peach.v3*, *Watermelon.v2*, and 497 *Orange.v8*—demonstrate consistent performance improvements over strong baselines across multiple evaluation metrics, including AP@0.5, AP@0.75, AP@[.50:.95], Precision, and Recall@all. The proposed method remains robust under challenging conditions such as texture similarity, intra-class variation, and partial occlusion, supporting deployment in real-world orchard scenarios.

Ablation analyses further verify the contributions of individual modules. MSA enhances object saliency through mask-guided attention, while FER improves feature representation via residual-based modulation. CLIP-guided semantic prompts improve category localization, particularly in data-limited and visually ambiguous settings. The integration of AWE facilitates adaptive fusion of semantic and visual features, improving detection reliability with negligible computational cost. Grad-CAM (Selvaraju et al., 2017; Sattarzadeh et al., 2021) visualizations confirm that each stage progressively sharpens the model’s spatial focus toward biologically meaningful regions.

Regarding computational efficiency, all components—including SFM and AWE—are lightweight by design. CLIP and SAM are utilized as offline preprocessing modules, minimizing inference-time overhead. The complete model achieves real-time operation with marginal increases in parameter count and FLOPs, making it suitable for deployment on edge devices and mobile agricultural platforms.

In summary, the proposed framework offers a scalable and efficient solution for few-shot agricultural species detection. It achieves strong performance under low-data conditions while maintaining low computational requirements, thereby contributing to long-term objectives in crop biodiversity monitoring, digital germplasm preservation, and intelligent orchard management.

## 4 Discussion

This section presents a detailed analysis of the proposed fruit-oriented few-shot detection framework from four key perspectives: performance attribution, robustness and adaptive fusion, computational efficiency, and limitations with future directions. The discussion is grounded in the experimental findings in Section 3, emphasizing how the lightweight tri-modal fusion design supports effective recognition in agricultural environments.

Building upon the quantitative improvements presented earlier, we first examine the effectiveness and source of gains in detection performance. Consistent performance gains observed across four benchmark fruit datasets—*Cantaloupe.v2*, *Peach.v3*, *Watermelon.v2*, and *Orange.v8*—demonstrate the effectiveness of the proposed tri-modal detection framework. As shown in **Table 1**, the model consistently outperforms baselines across key metrics, including AP@0.5, AP@[.50:.95], Precision, and Recall@all. For instance, on *Cantaloupe.v2*, AP@0.5 increases from 0.8611 (baseline) to 0.9403 (full model), while AP@[.50:.95] improves from 0.5652 to 0.6342. These improvements highlight the contributions of each integrated component.

The majority of the performance gains can be attributed to the Semantic Fusion Modulator (SFM), which incorporates the Mask-Saliency Adapter (MSA) and Feature Enhancement Recomposer (FER). MSA introduces structure-aware saliency using SAM-derived, class-agnostic masks to guide attention toward biologically relevant regions. FER further refines the features via residual modulation, enhancing boundary awareness and object completeness.

The effectiveness of CLIP-guided semantic prompting is particularly evident in ambiguous visual scenarios, where category cues from language help stabilize attention. As illustrated in **Figure 9**, attention maps evolve from coarse activations in the baseline to structured object-level focus with the addition of MSA, FER, and CLIP. These observations are consistent with the quantitative trends and confirm the synergy among the three modalities.

Beyond performance improvements, the robustness of the framework under real-world agricultural conditions is another key advantage. To maintain robustness under conditions such as occlusion, lighting variation, and background interference, the Attention-Aware Weight Estimator (AWE) is introduced. In contrast to static or manually tuned fusion schemes, AWE dynamically reweights semantic-enhanced and original RGB features through a lightweight MLP guided by global saliency cues. This enables real-time adaptive fusion, even in partially degraded or visually ambiguous images, which frequently occur in orchard and open-field settings. The negligible computational cost of AWE supports its practical use in robust multimodal integration.

In addition to architectural robustness, we further validated the stability of the proposed framework under different dataset partitioning schemes (see **Appendix Table 2**). Specifically, we compared the detection performance of 7:2:1 and 8:1:1 train–validation–test splits across the four benchmark datasets. The results showed minimal variation, with AP@0.5, Precision, and Recall differing by less than ±0.02. For example, *Cantaloupe.v2* achieved a ΔAP@0.5 of +0.012, *Orange.v8* varied by −0.008, while *Peach.v3* and *Watermelon.v2* changed by +0.007 and −0.010, respectively. The corresponding *t*-values (0.66–0.86) and *p*-values (*>* 0.40) indicate that these differences are not statistically significant. These findings confirm that the proposed framework maintains consistent accuracy and stability across varying data split ratios, underscoring its robustness to differences in sample distribution.

The proposed method also emphasizes computational efficiency and deployment feasibility. The framework is designed with deployment efficiency in mind. Integrating MSA, FER, and AWE results in a model size increase of only 0.33M parameters and an additional inference latency of 1.6ms (**Table 3**). Notably, CLIP and SAM are applied exclusively during offline preprocessing and thus incur no extra runtime overhead. The final model sustains over 108 FPS, enabling deployment on edge platforms such as mobile inspection robots, UAVs for orchard monitoring, and embedded phenotyping systems. This efficient design ensures a favorable balance between accuracy and practical deployment feasibility in agricultural scenarios.

Nevertheless, it is important to recognize the limitations and potential areas for future work. Despite its effectiveness, the framework has certain limitations. The current single-scale feature design may limit sensitivity to small, occluded, or densely clustered fruit targets. In particular, we observed that in cases with very small fruit instances or scenes containing a large number of overlapping targets, the framework may occasionally produce incomplete or merged detections. These failure cases highlight the challenge of balancing fine-grained spatial precision with global semantic consistency.

Furthermore, we acknowledge that while the framework exhibits strong robustness under moderate occlusion and illumination variation, its performance may degrade under more adverse visual conditions such as heavy noise, motion blur, or severe defocus. In such cases, the quality of the SAM-derived structural priors can deteriorate, leading to suboptimal localization and weakened semantic guidance. Future extensions may address these challenges through noise-robust training, synthetic perturbation augmentation, or self-distillation schemes that enhance the stability of multimodal features under degraded visual quality.

Future work may explore multi-scale feature alignment or pyramid-based fusion to enhance spatial adaptability. Additionally, integrating the tri-modal architecture with Transformer-based detectors [e.g. DINO (Zhang et al., 2022), Deformable-DETR (Zhu et al., 2020)] may improve long-range context modeling. Another promising direction involves uncertainty-aware or entropy-guided fusion strategies to better address rare fruit varieties and visually ambiguous categories.

In summary, the proposed method achieves a practical trade-off between semantic consistency, structural awareness, and computational efficiency. It offers a robust and interpretable solution for few-shot fruit detection, with promising applicability to biodiversity monitoring, varietal classification, and digital germplasm documentation.

## 5 Conclusions

This paper proposes a lightweight and interpretable tri-modal fusion framework for few-shot object detection in agricultural contexts, with emphasis on fruit diversity recognition and digital orchard archiving. To address key challenges such as limited annotations, visual ambiguity, and complex backgrounds, the framework integrates a Semantic Fusion Modulator (SFM) and an Attention-Aware Weight Estimator (AWE) into the YOLOv12 architecture, while preserving the original detection backbone to ensure deployment efficiency.

The SFM consists of two complementary modules: the Mask-Saliency Adapter (MSA) and the Feature Enhancement Recomposer (FER). MSA utilizes class-agnostic masks generated by the Segment Anything Model (SAM) to produce structure-aware saliency maps that direct attention to biologically relevant regions, such as fruit contours and surfaces. FER further refines visual features via residual modulation, enhancing boundary sensitivity and context adaptation. Together, these modules improve localization and feature discrimination in cluttered and occluded scenes.

Additionally, a lightweight AWE dynamically reweights RGB and semantic-enhanced features based on global saliency cues derived from a shallow MLP. Importantly, CLIP and SAM are used solely as offline preprocessing components, introducing no additional inference-time latency. This design ensures effective semantic guidance without compromising real-time performance.

Experiments on four few-shot fruit datasets—*Cantaloupe.v2*, *Peach.v3*, *Watermelon.v2*, and *Orange.v8*— demonstrate consistent outperformance over baselines including Meta R-CNN, DeFRCN, and TFA-WO-FPN across metrics such as AP@0.5, AP@[.50:.95] and Recall@all. Grad-CAM visualizations confirm that MSA and FER yield more coherent and semantically meaningful attention distributions. Ablation studies validate their individual and joint contributions. The final model maintains a compact architecture with an inference latency of 9.2 ms and a frame rate exceeding 108 FPS, supporting deployment on edge agricultural systems.

In summary, the proposed framework achieves a practical trade-off among detection accuracy, computational efficiency, and model interpretability, offering a scalable solution for tasks such as cultivar classification, germplasm documentation, and automated fruit diversity monitoring.

Future directions include extending the framework to Transformer-based detection architectures (e.g. DINO, Deformable DETR) to enhance long-range context modeling. Replacing the current score-based fusion with uncertainty-aware confidence calibration is another potential improvement. Furthermore, developing an end-to-end pipeline that jointly learns prompt generation, mask extraction, and adaptive fusion without external precomputed inputs could enhance model autonomy and robustness in real-world agricultural deployments.

## Data Availability

The raw data supporting the conclusions of this article will be made available by the authors, without undue reservation.
