# Supplementary figures and images for: A lightweight tri-modal few-shot detection framework for fruit diversity recognition toward digital orchard archiving
Source: Front Plant Sci. 2025 Dec 1;16:1696622. doi: 10.3389/fpls.2025.1696622 (PMC12703783; doi:10.3389/fpls.2025.1696622)

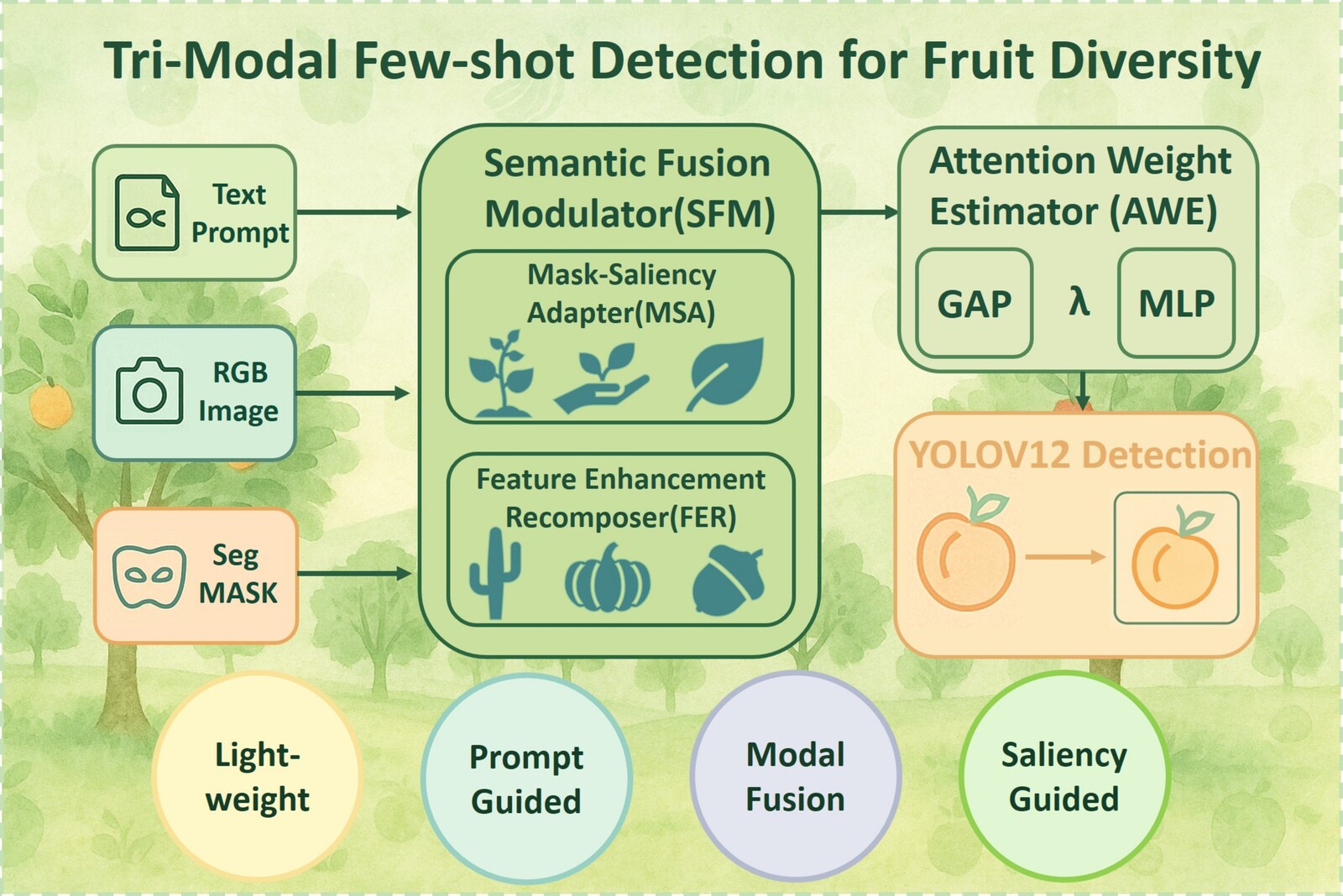

Supplement: Supplementary file 1 [file DataSheet1.zip › Fig1.jpg]

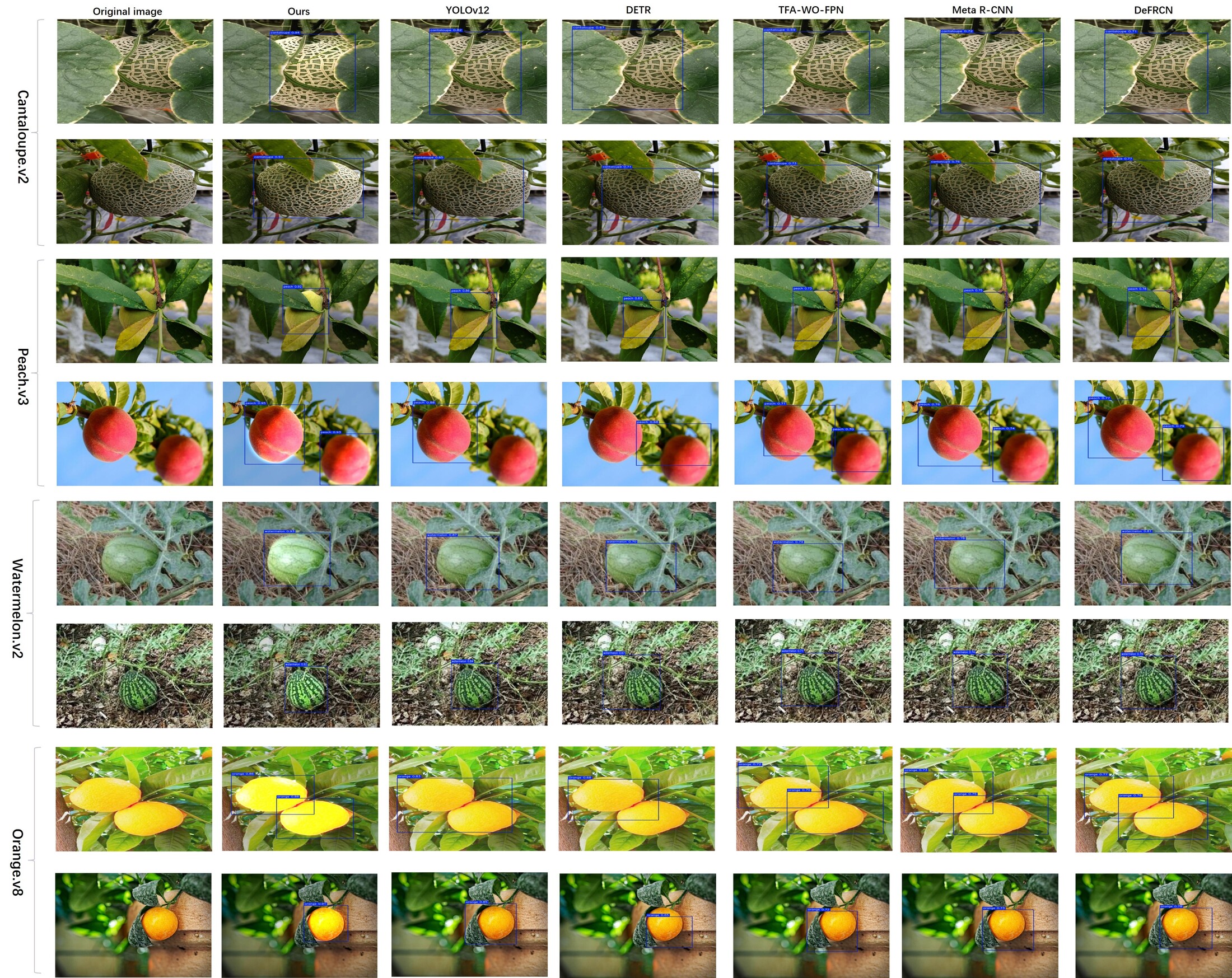

Supplement: Supplementary file 1 [file DataSheet1.zip › Fig10.jpg]

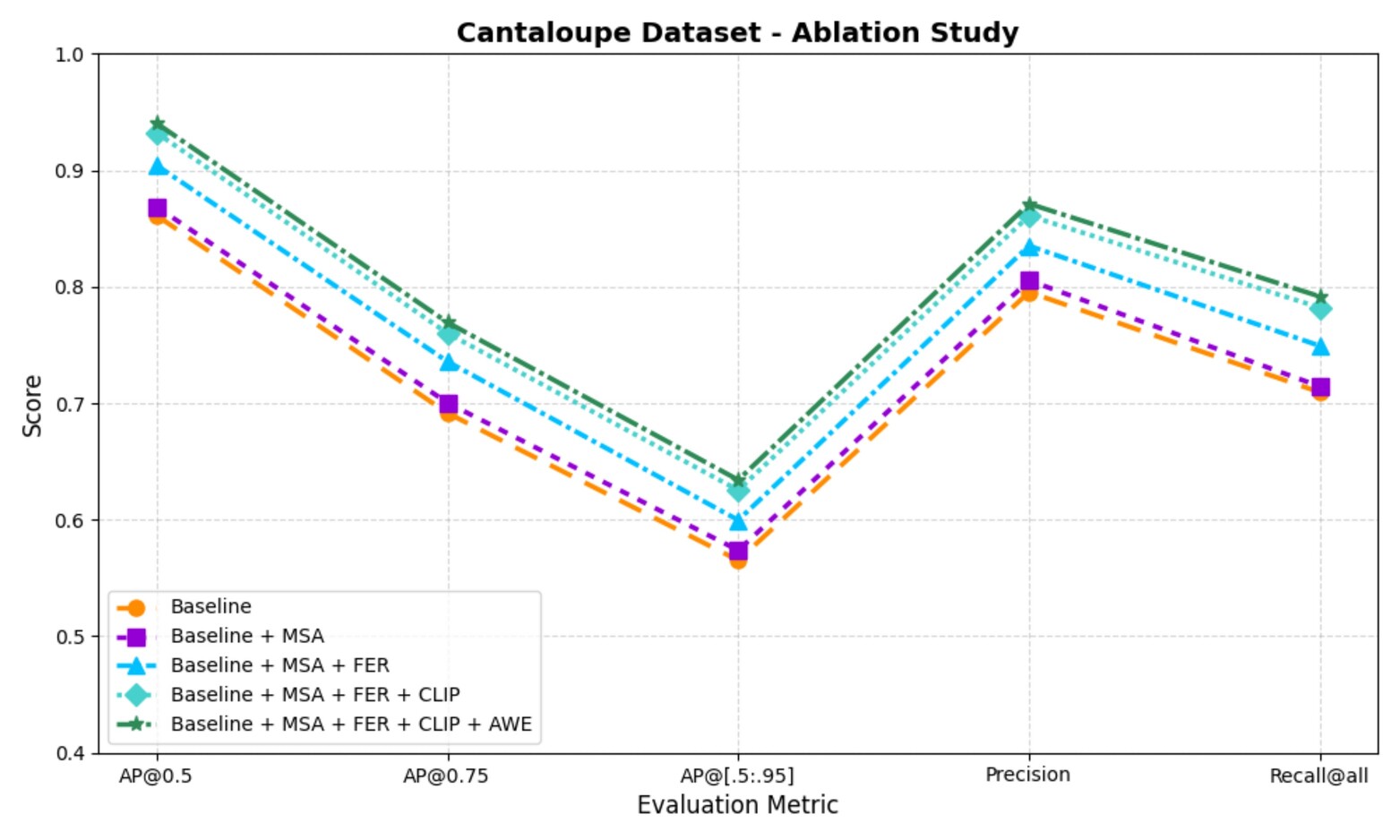

Supplement: Supplementary file 1 [file DataSheet1.zip › Fig11.jpg]

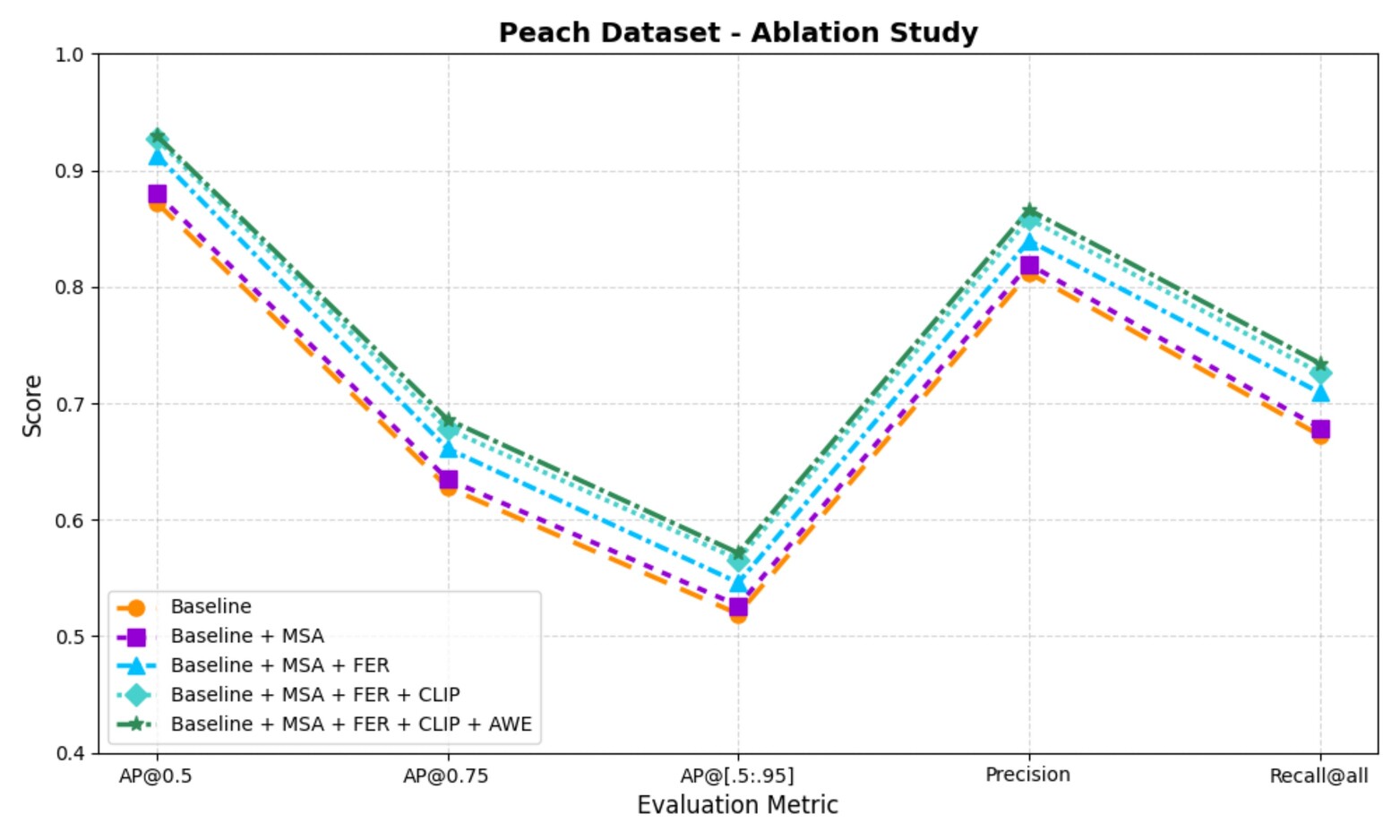

Supplement: Supplementary file 1 [file DataSheet1.zip › Fig12.jpg]

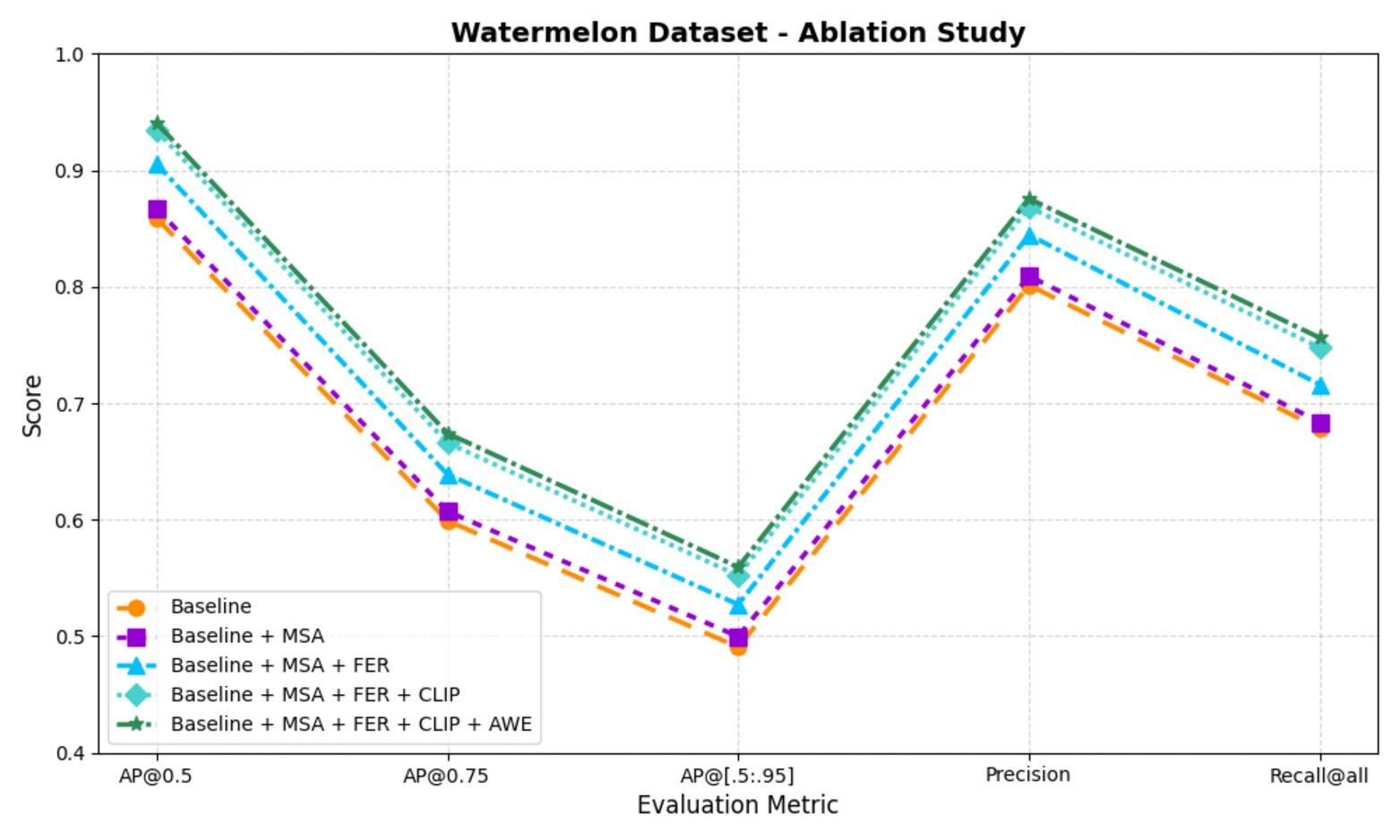

Supplement: Supplementary file 1 [file DataSheet1.zip › Fig13.jpg]

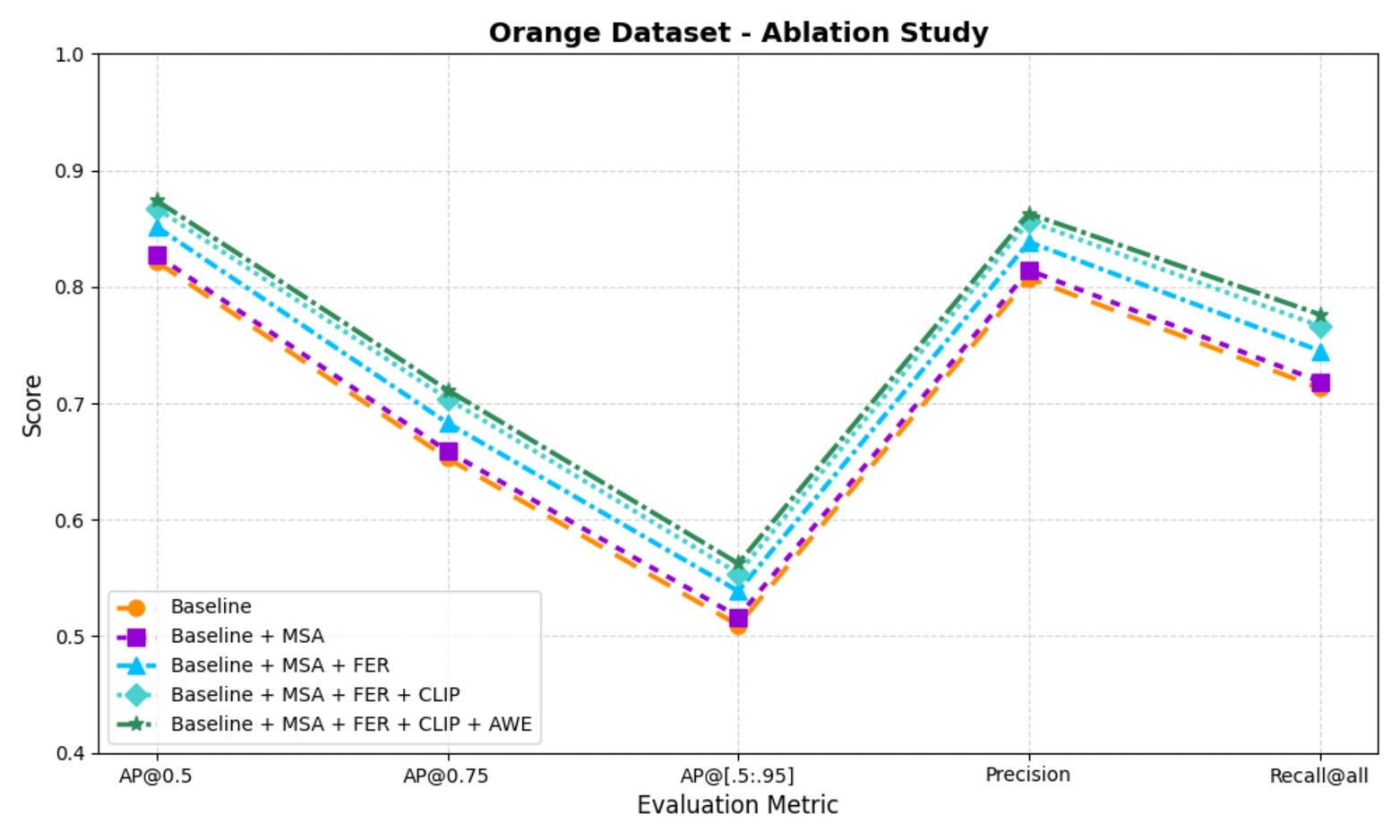

Supplement: Supplementary file 1 [file DataSheet1.zip › Fig14.jpg]

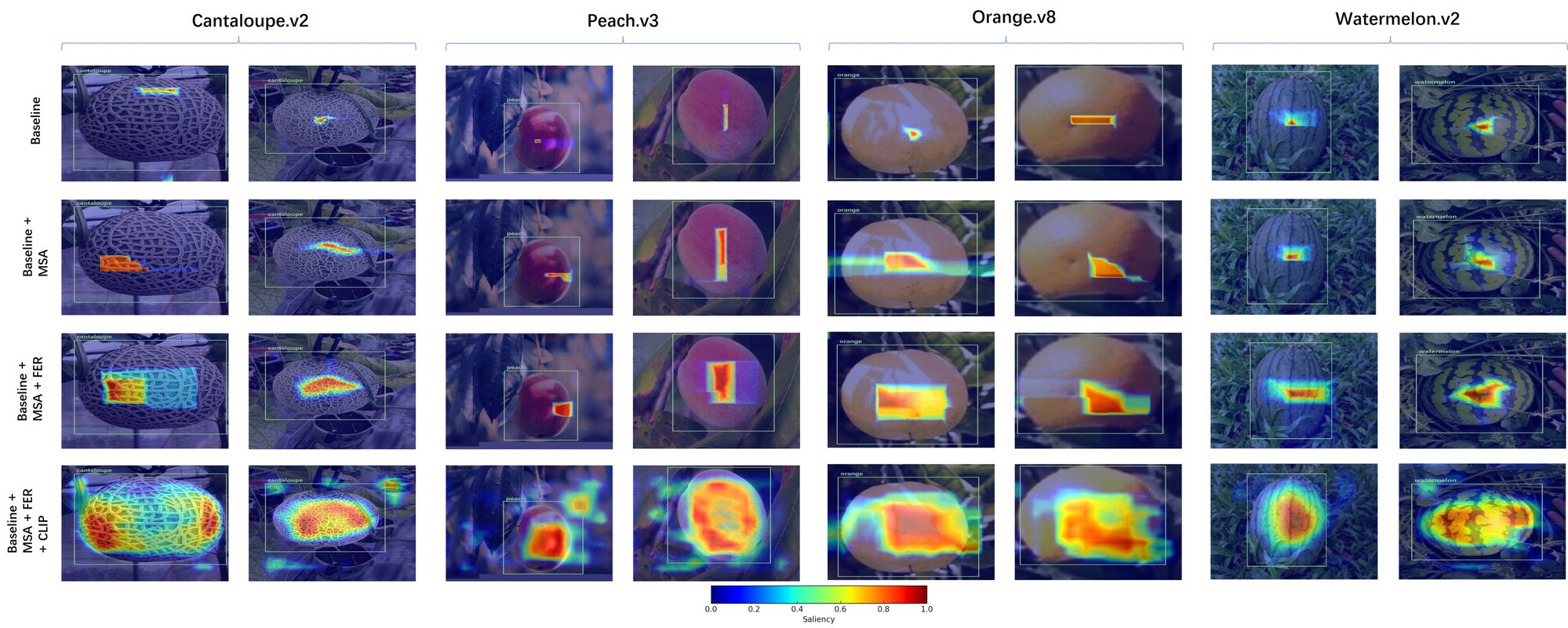

Supplement: Supplementary file 1 [file DataSheet1.zip › Fig15.jpg]

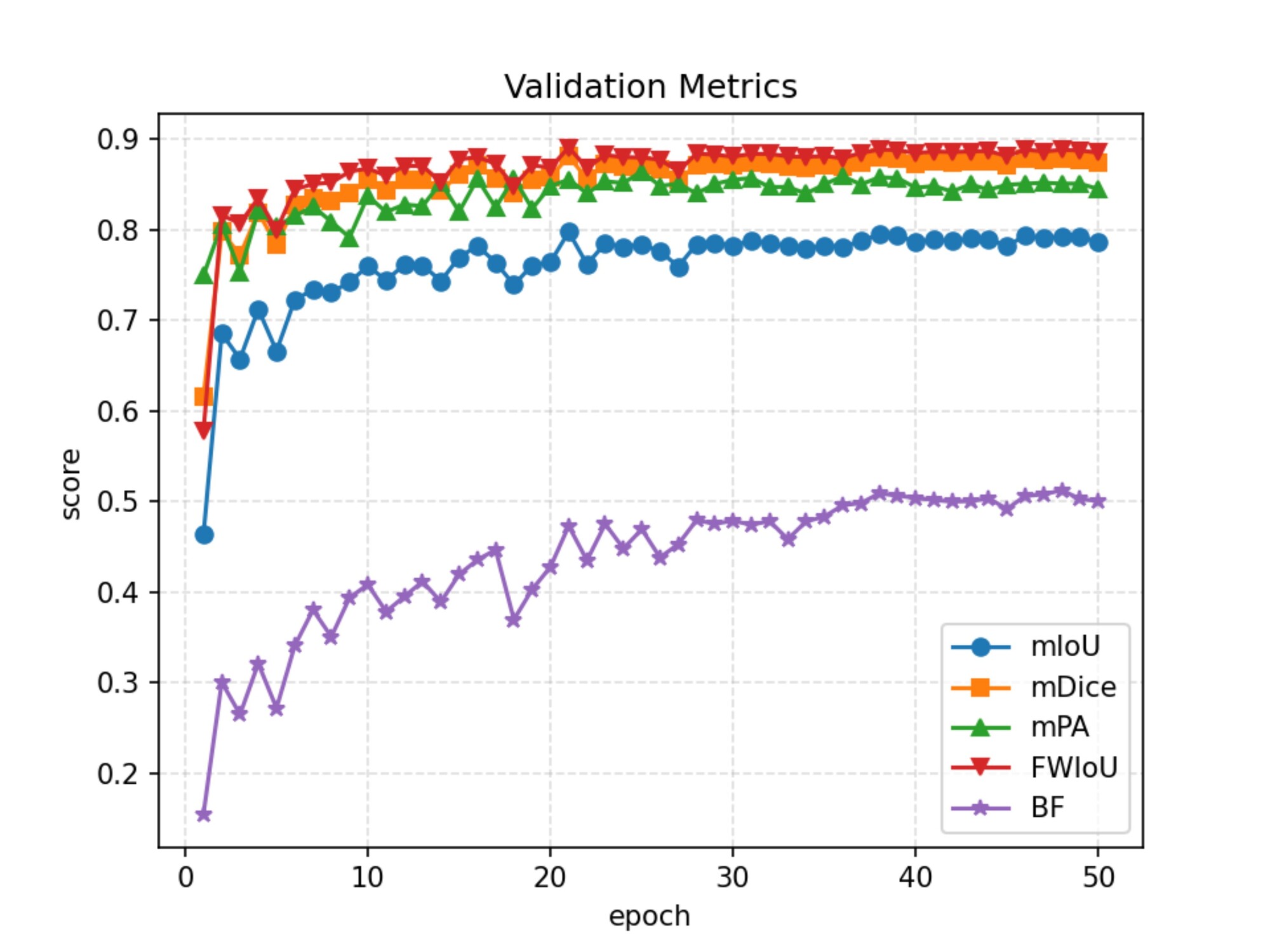

Supplement: Supplementary file 1 [file DataSheet1.zip › Fig16.jpg]

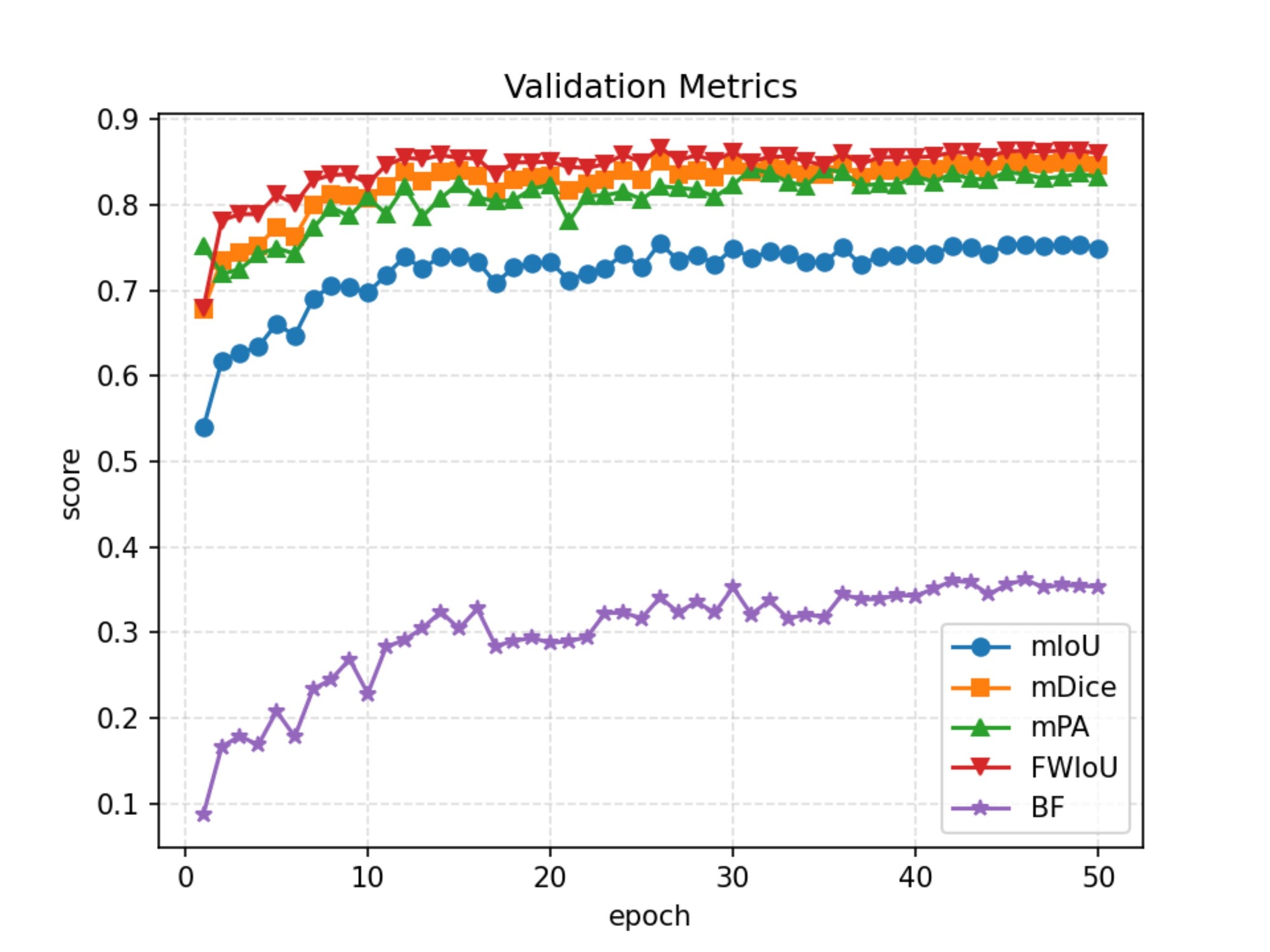

Supplement: Supplementary file 1 [file DataSheet1.zip › Fig17.jpg]

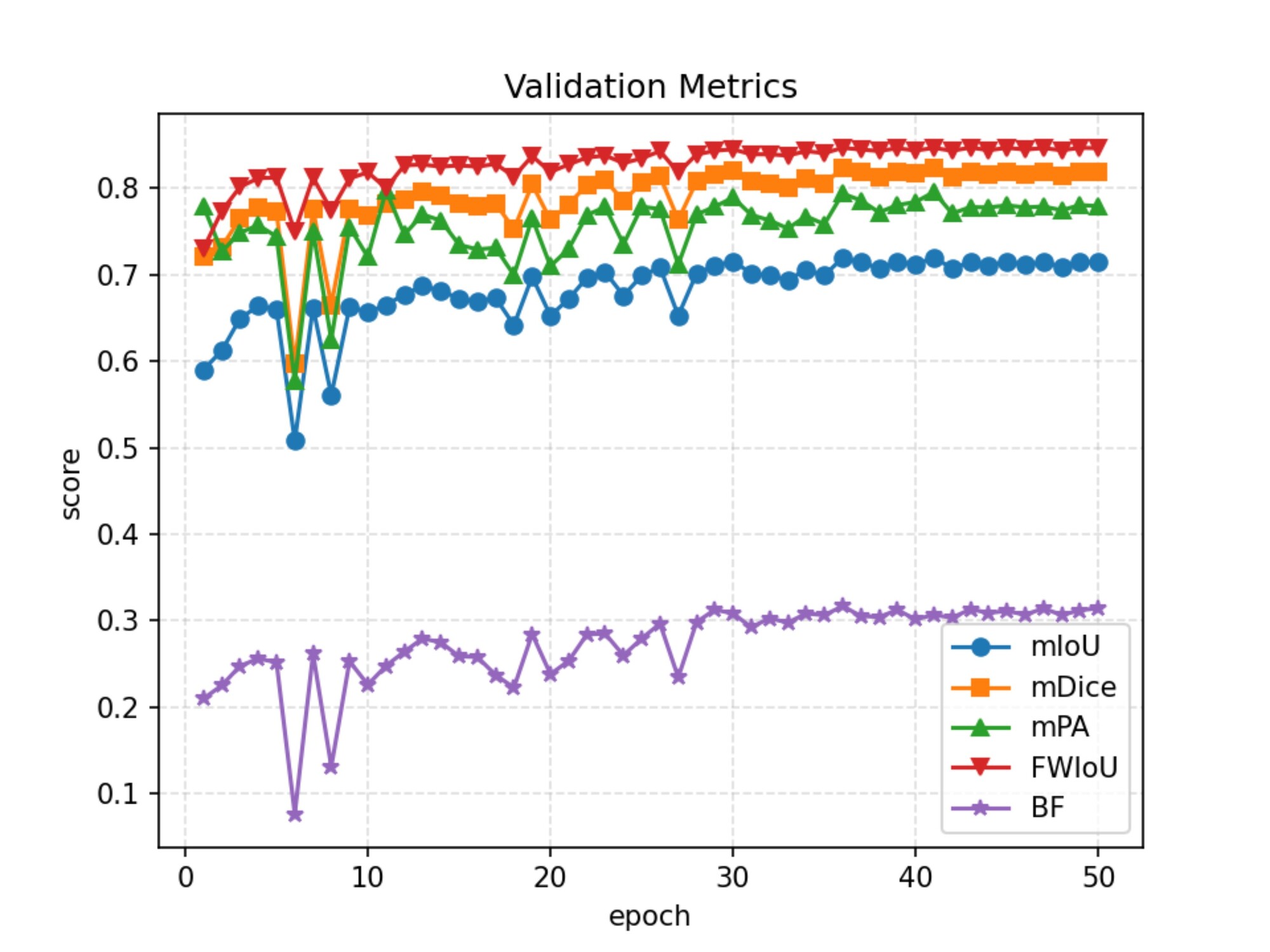

Supplement: Supplementary file 1 [file DataSheet1.zip › Fig18.jpg]

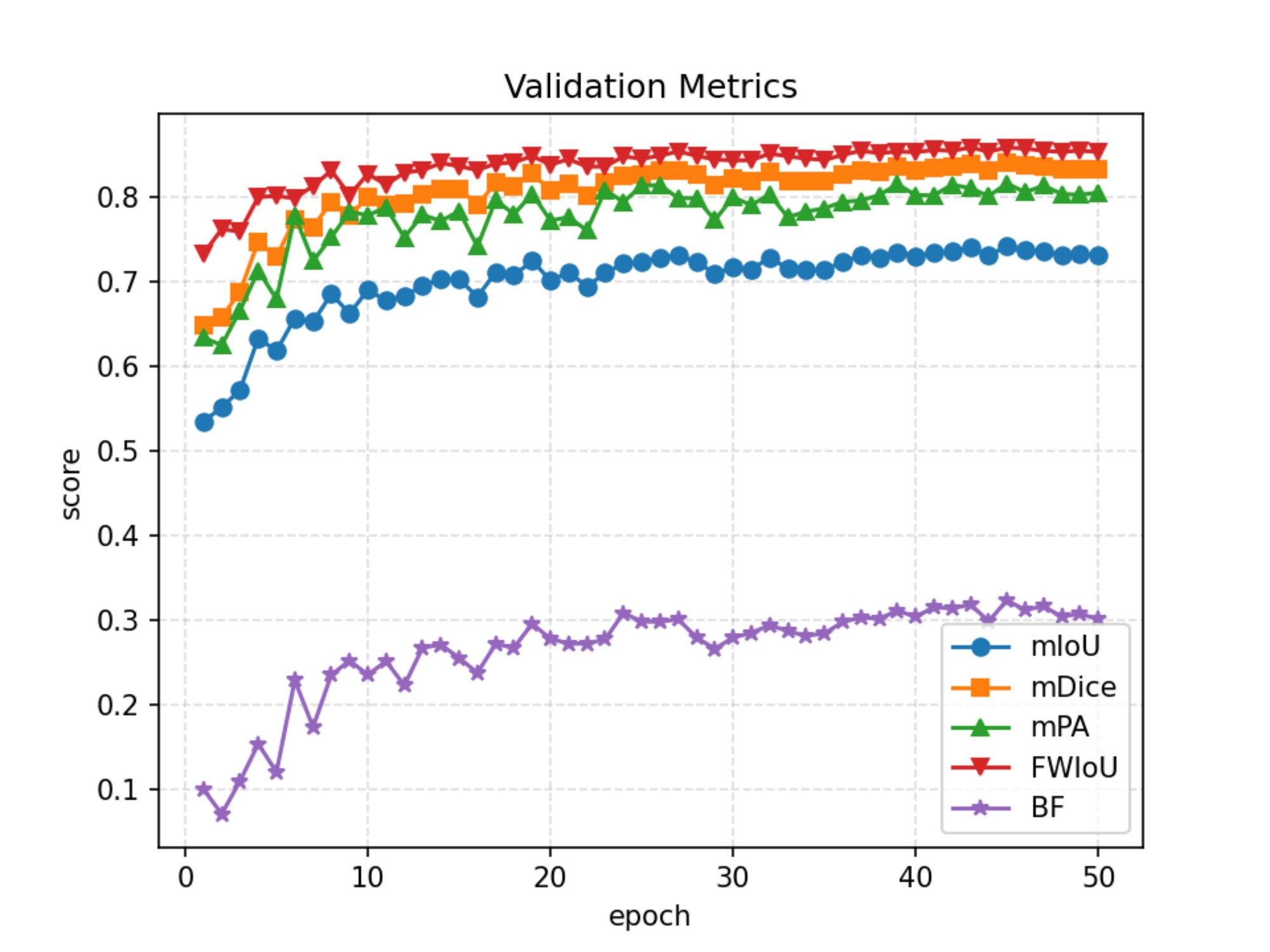

Supplement: Supplementary file 1 [file DataSheet1.zip › Fig19.jpg]

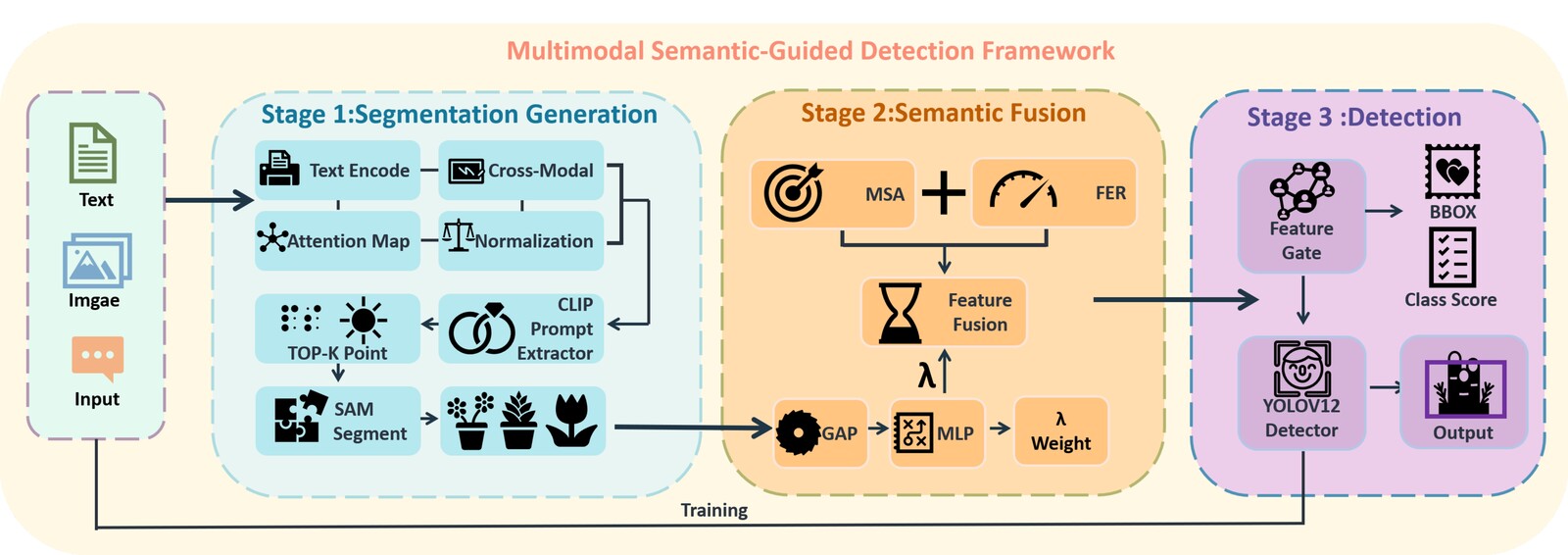

Supplement: Supplementary file 1 [file DataSheet1.zip › Fig2.jpg]

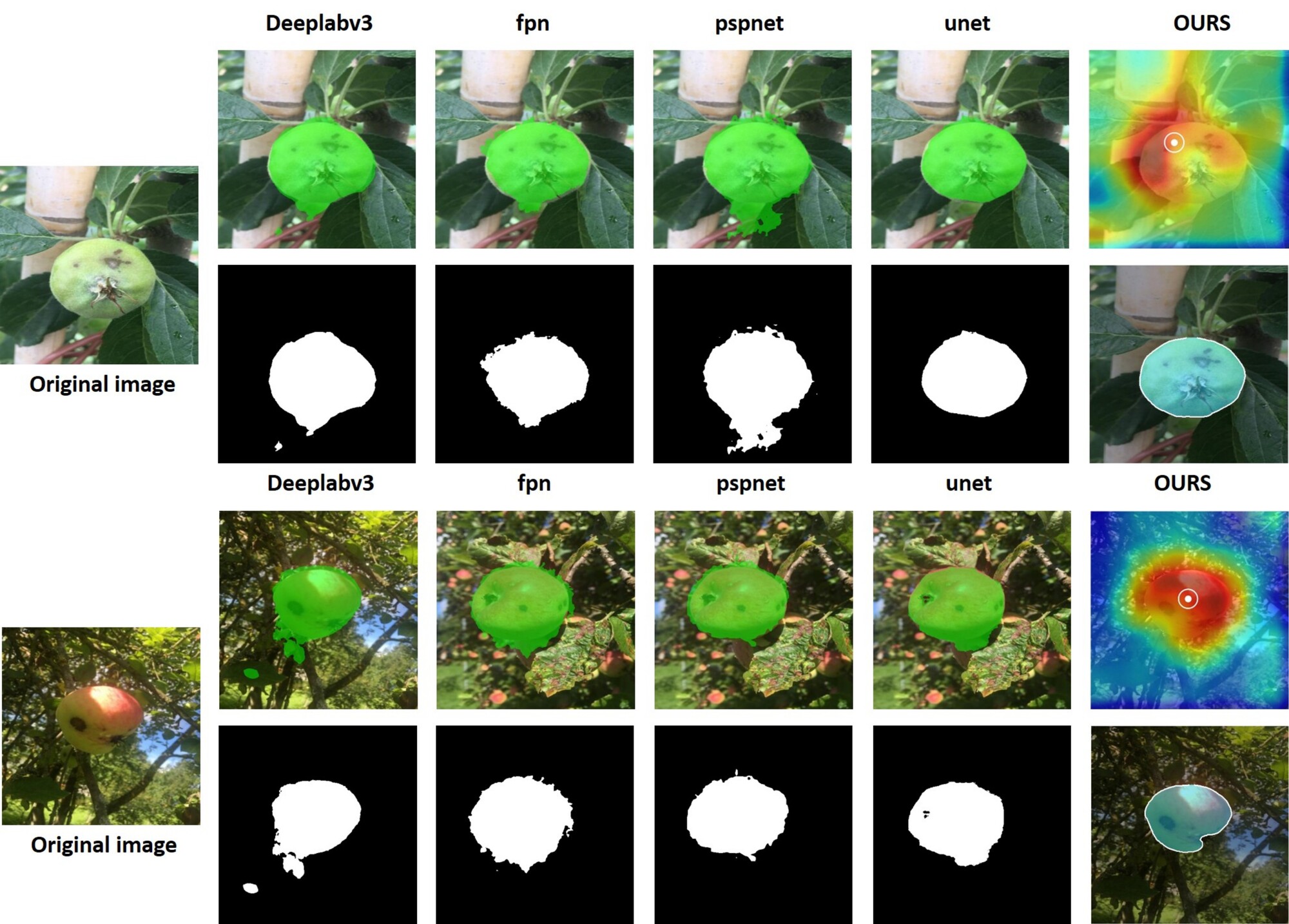

Supplement: Supplementary file 1 [file DataSheet1.zip › Fig20.jpg]

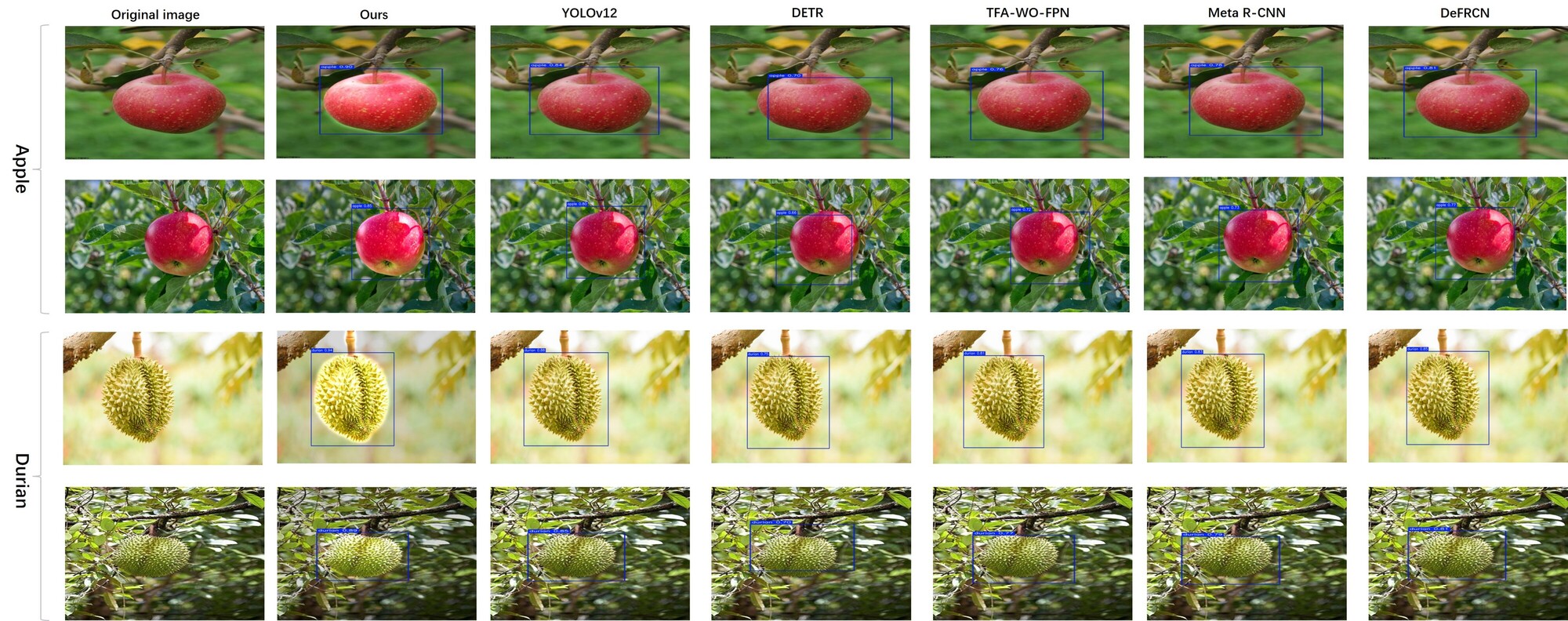

Supplement: Supplementary file 1 [file DataSheet1.zip › Fig21.jpg]

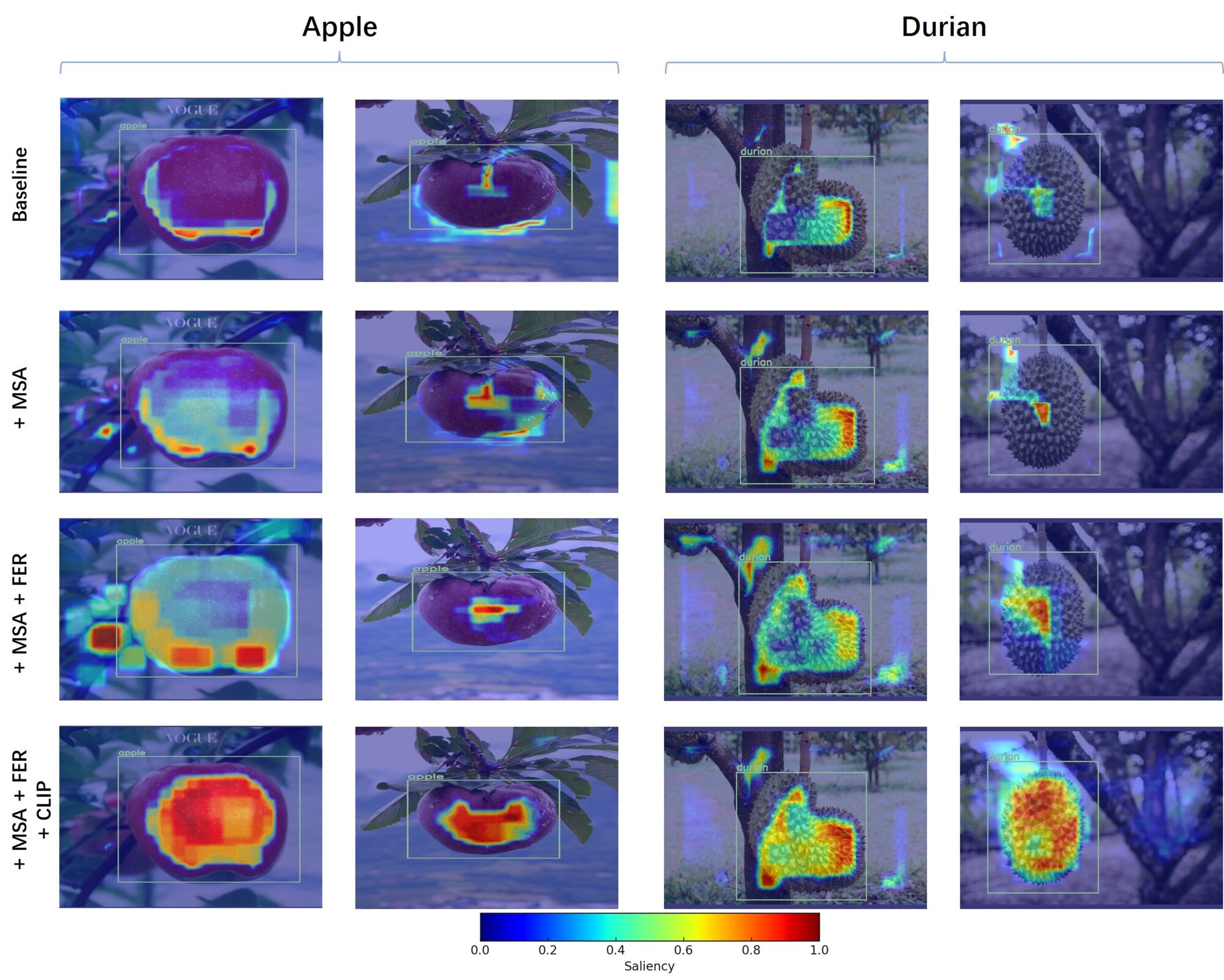

Supplement: Supplementary file 1 [file DataSheet1.zip › Fig22.jpg]

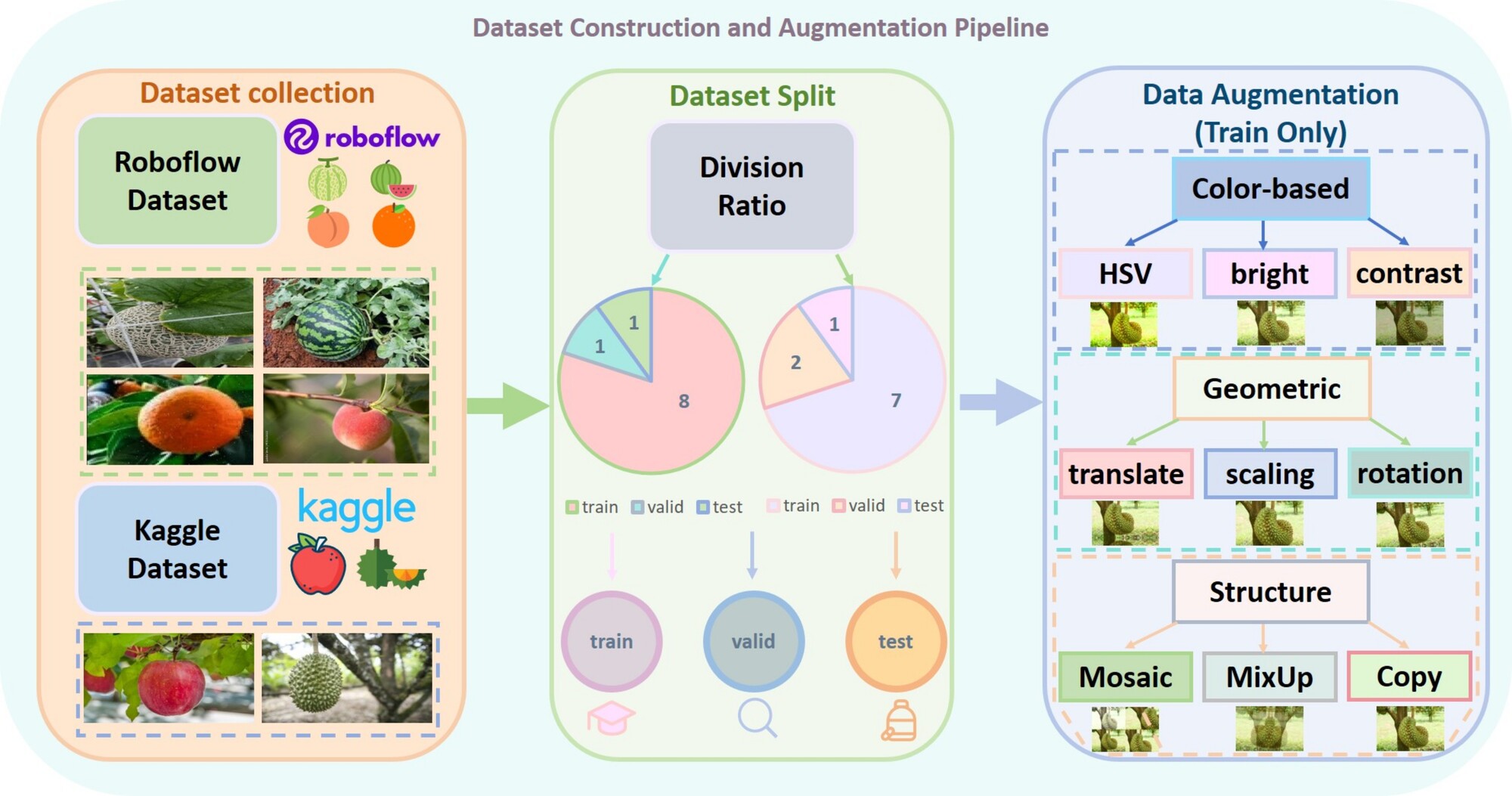

Supplement: Supplementary file 1 [file DataSheet1.zip › Fig23.jpg]

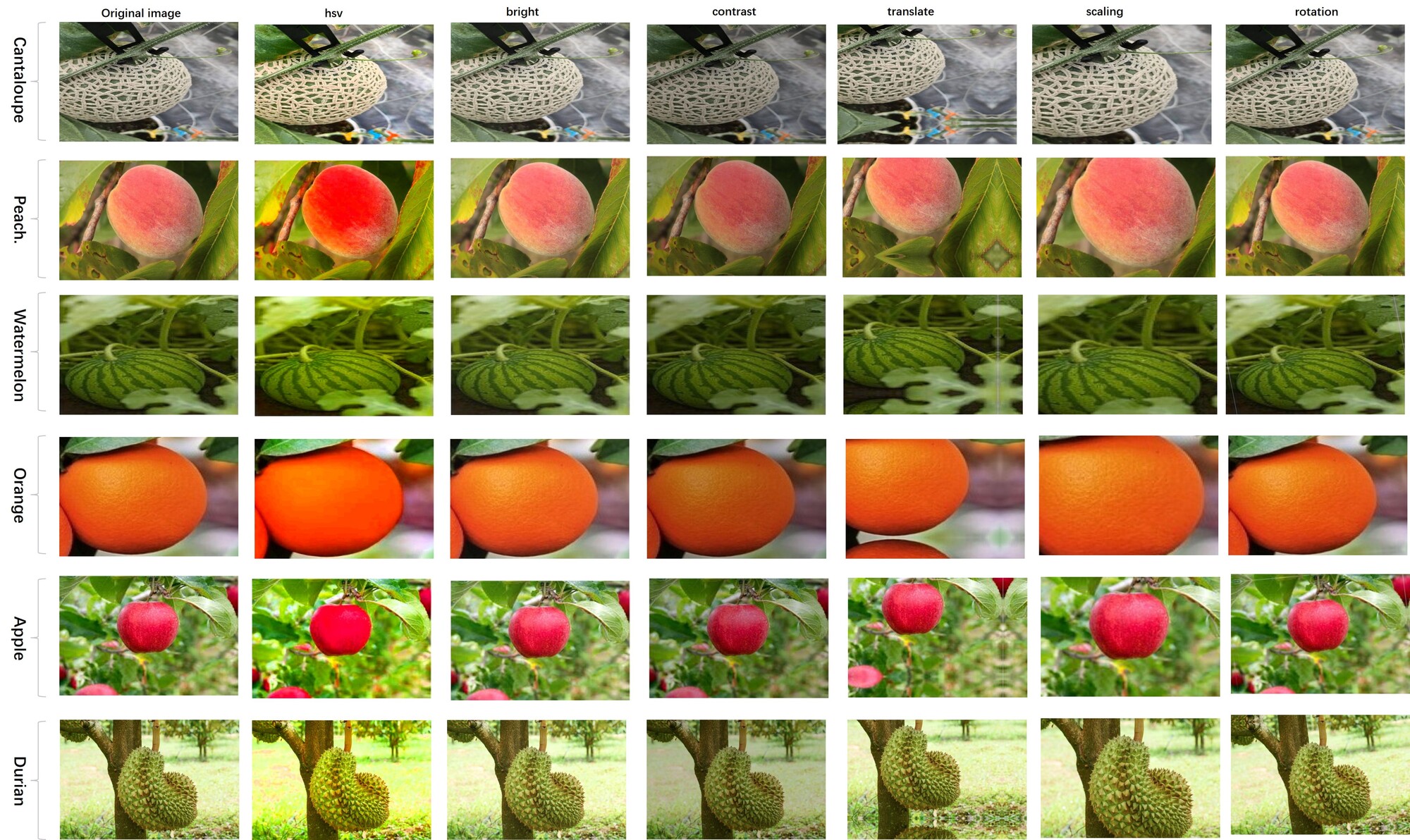

Supplement: Supplementary file 1 [file DataSheet1.zip › Fig24.jpg]

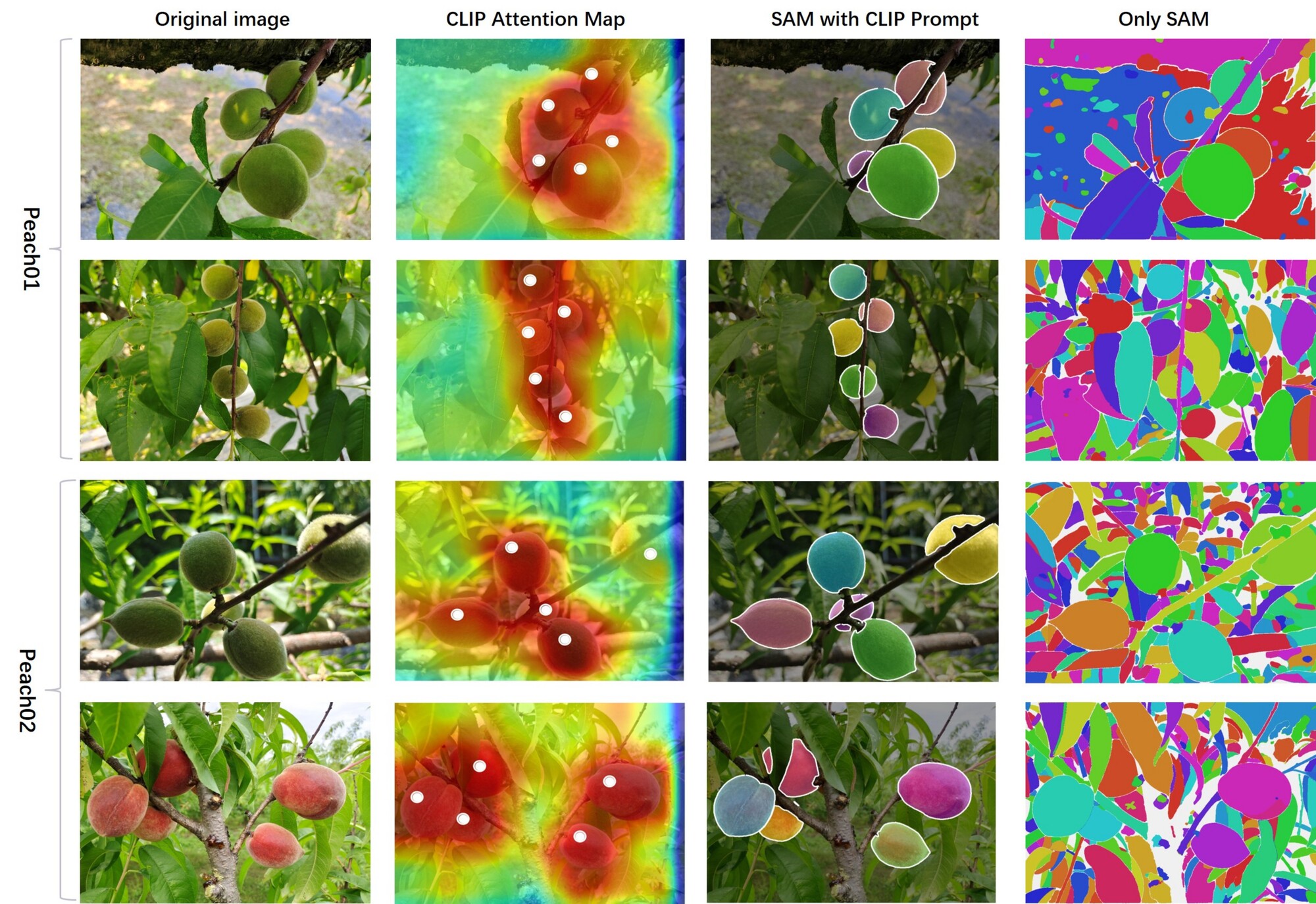

Supplement: Supplementary file 1 [file DataSheet1.zip › Fig25.jpg]

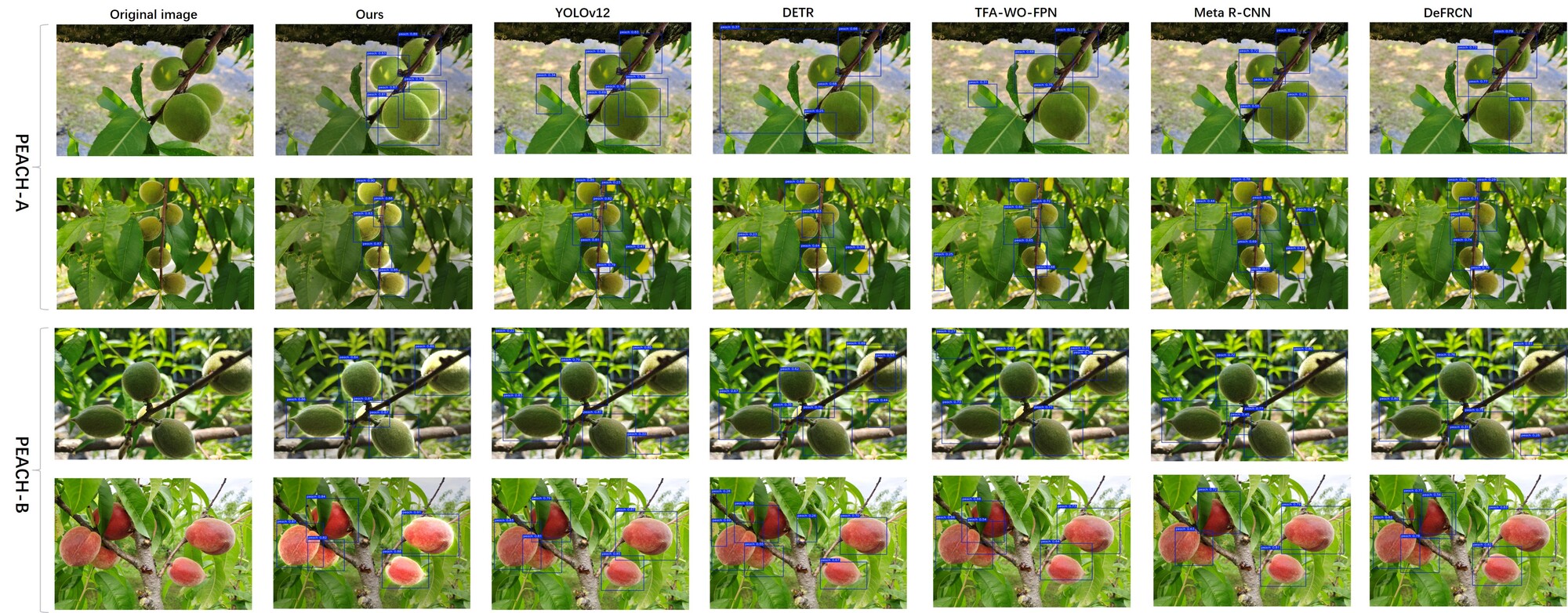

Supplement: Supplementary file 1 [file DataSheet1.zip › Fig26.jpg]

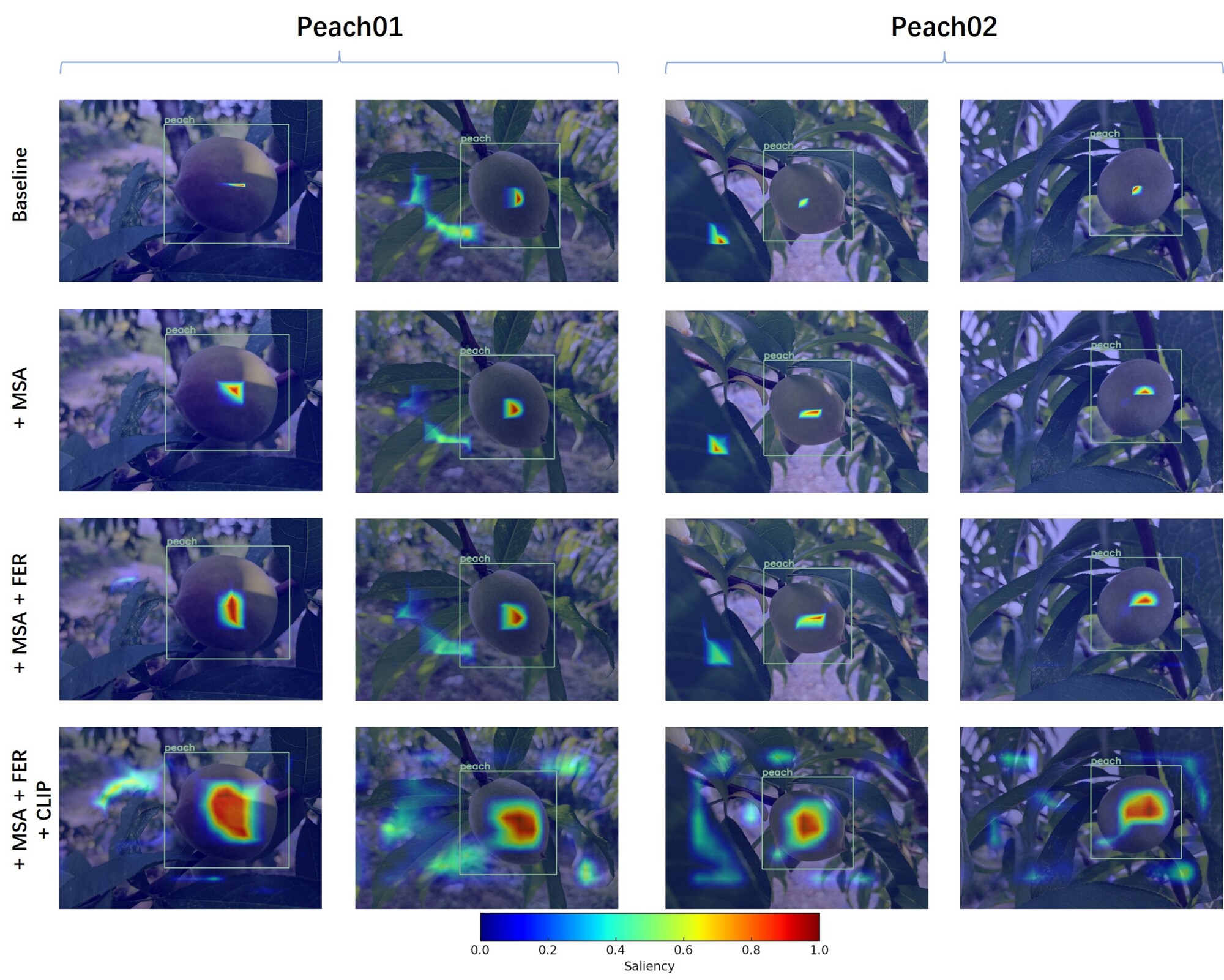

Supplement: Supplementary file 1 [file DataSheet1.zip › Fig27.jpg]

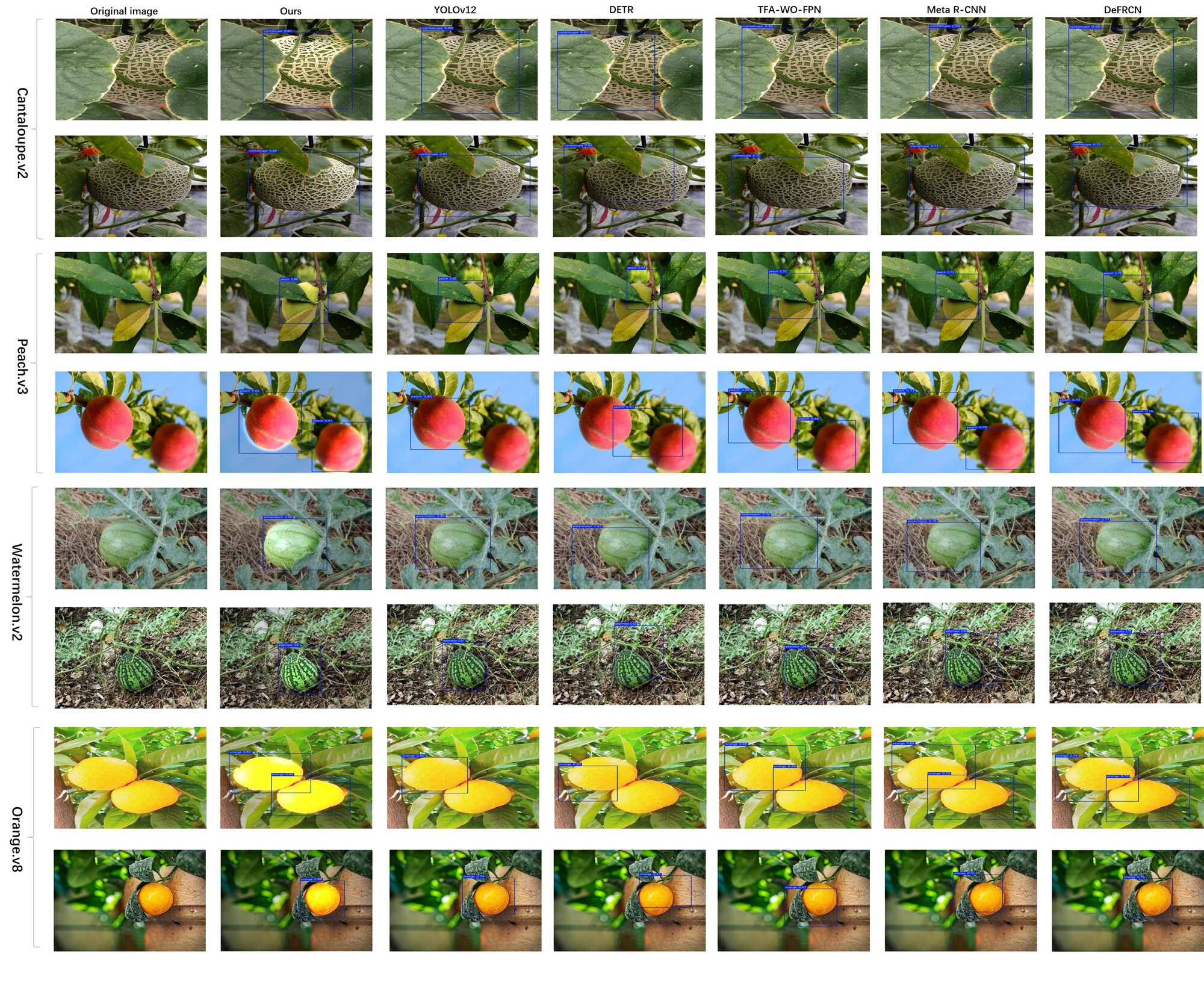

Supplement: Supplementary file 1 [file DataSheet1.zip › Fig28.jpg]

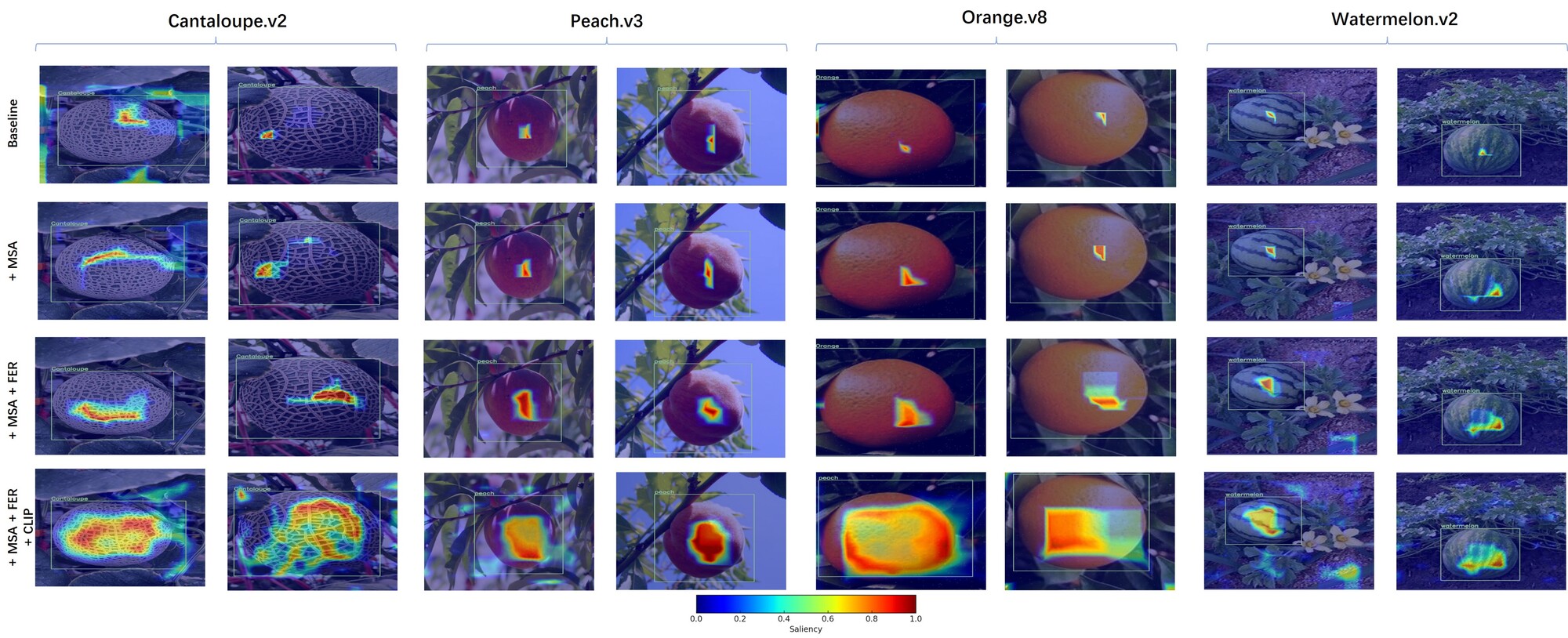

Supplement: Supplementary file 1 [file DataSheet1.zip › Fig29.jpg]

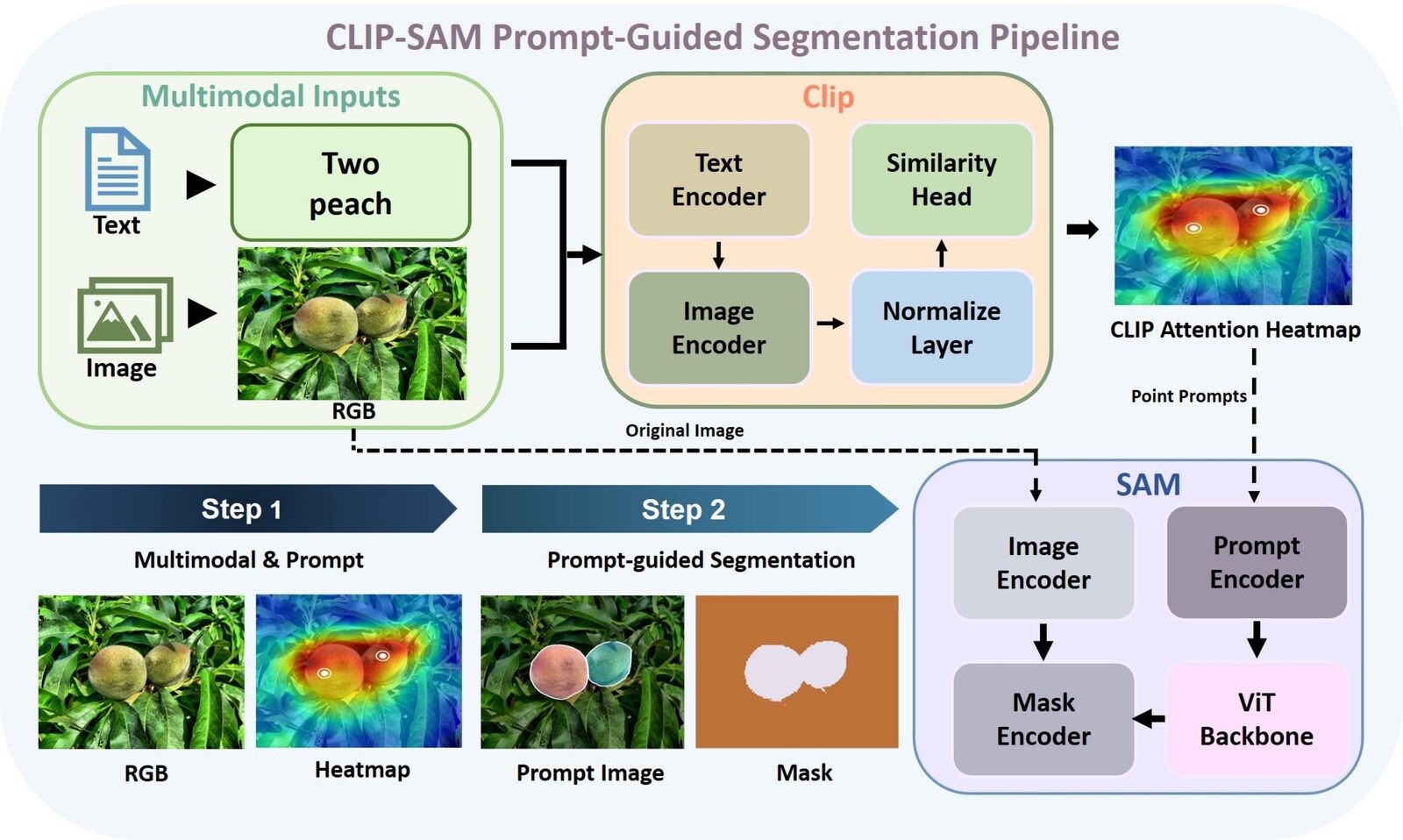

Supplement: Supplementary file 1 [file DataSheet1.zip › Fig3.jpg]

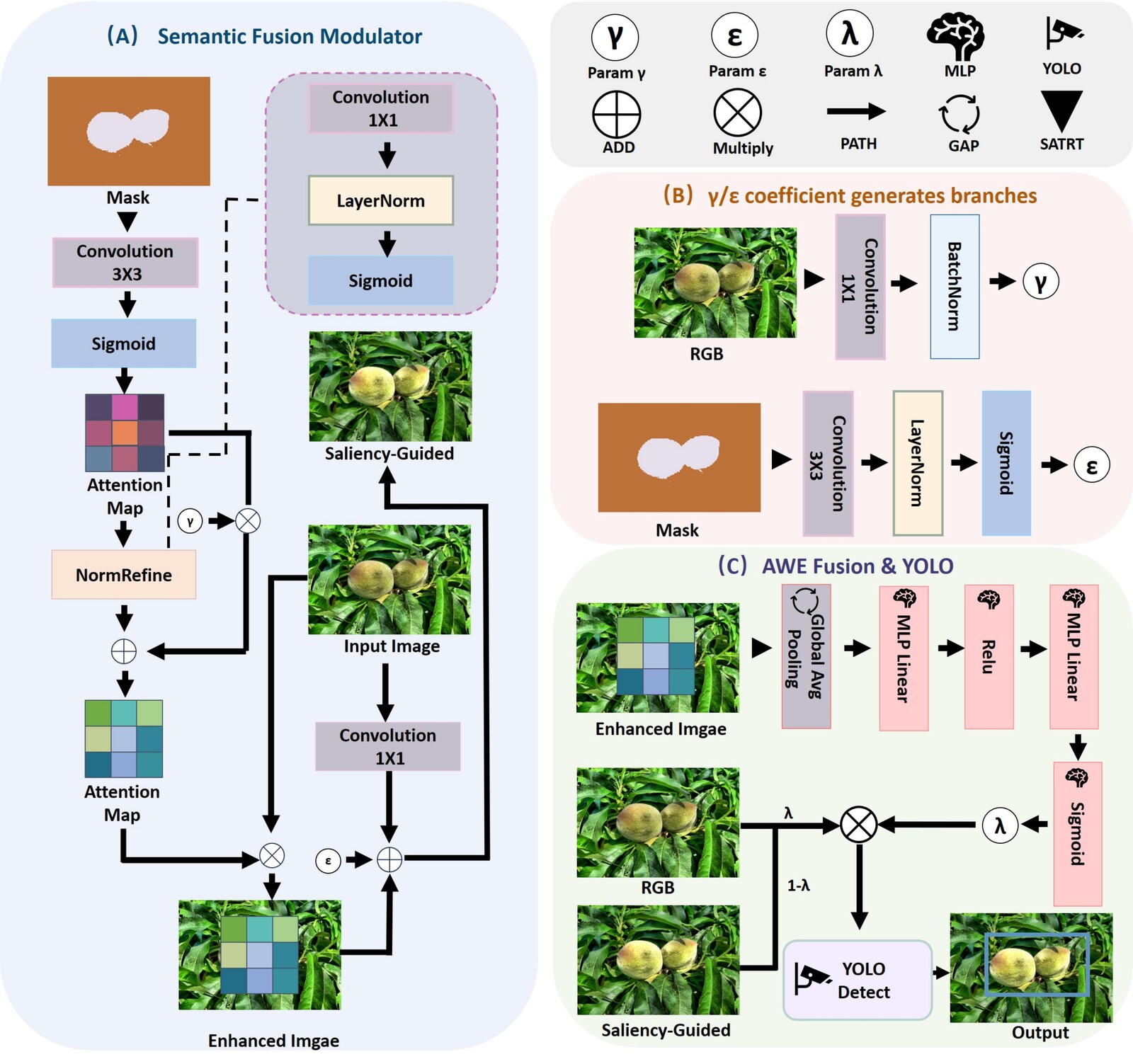

Supplement: Supplementary file 1 [file DataSheet1.zip › Fig4.jpg]

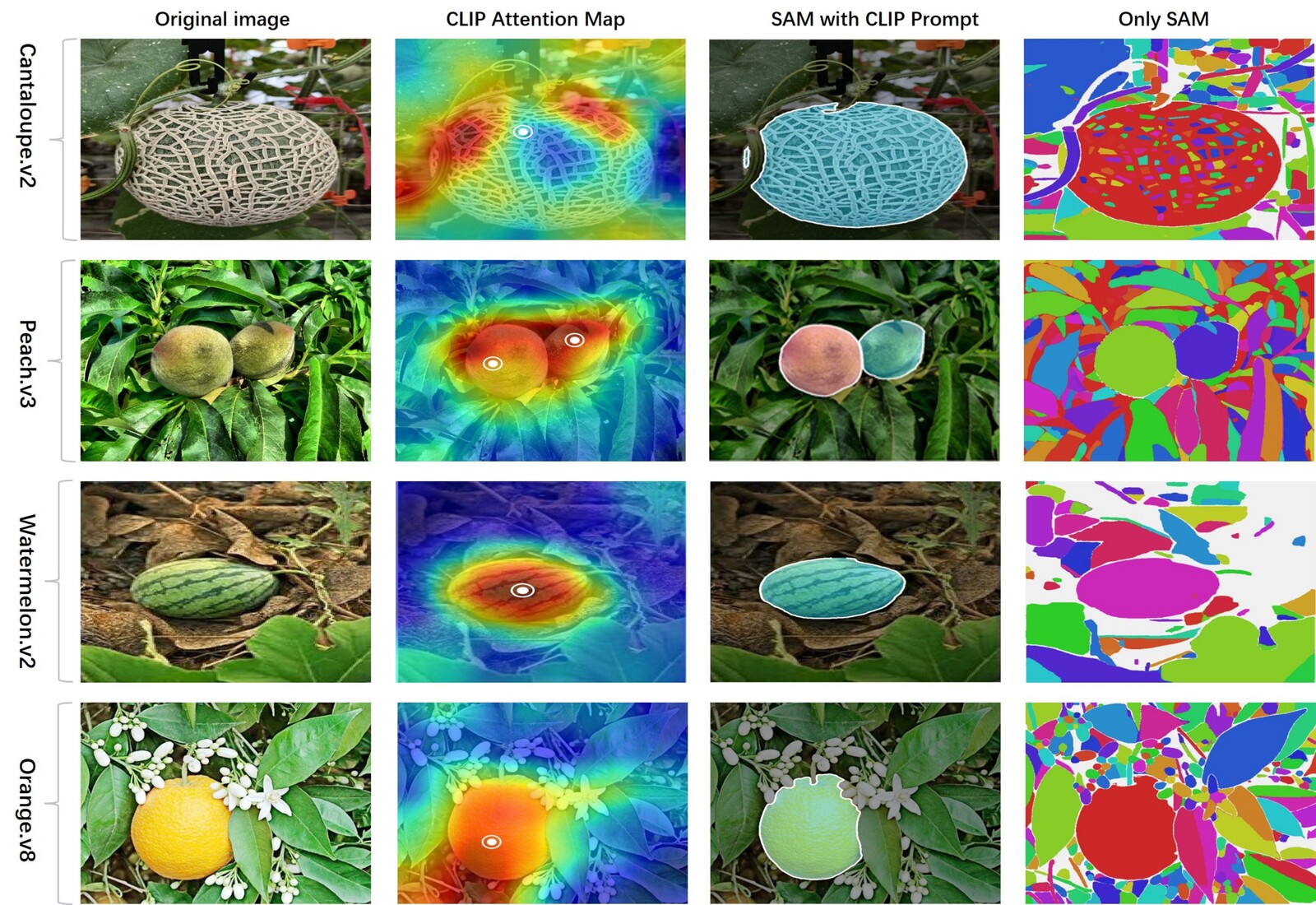

Supplement: Supplementary file 1 [file DataSheet1.zip › Fig5.jpg]

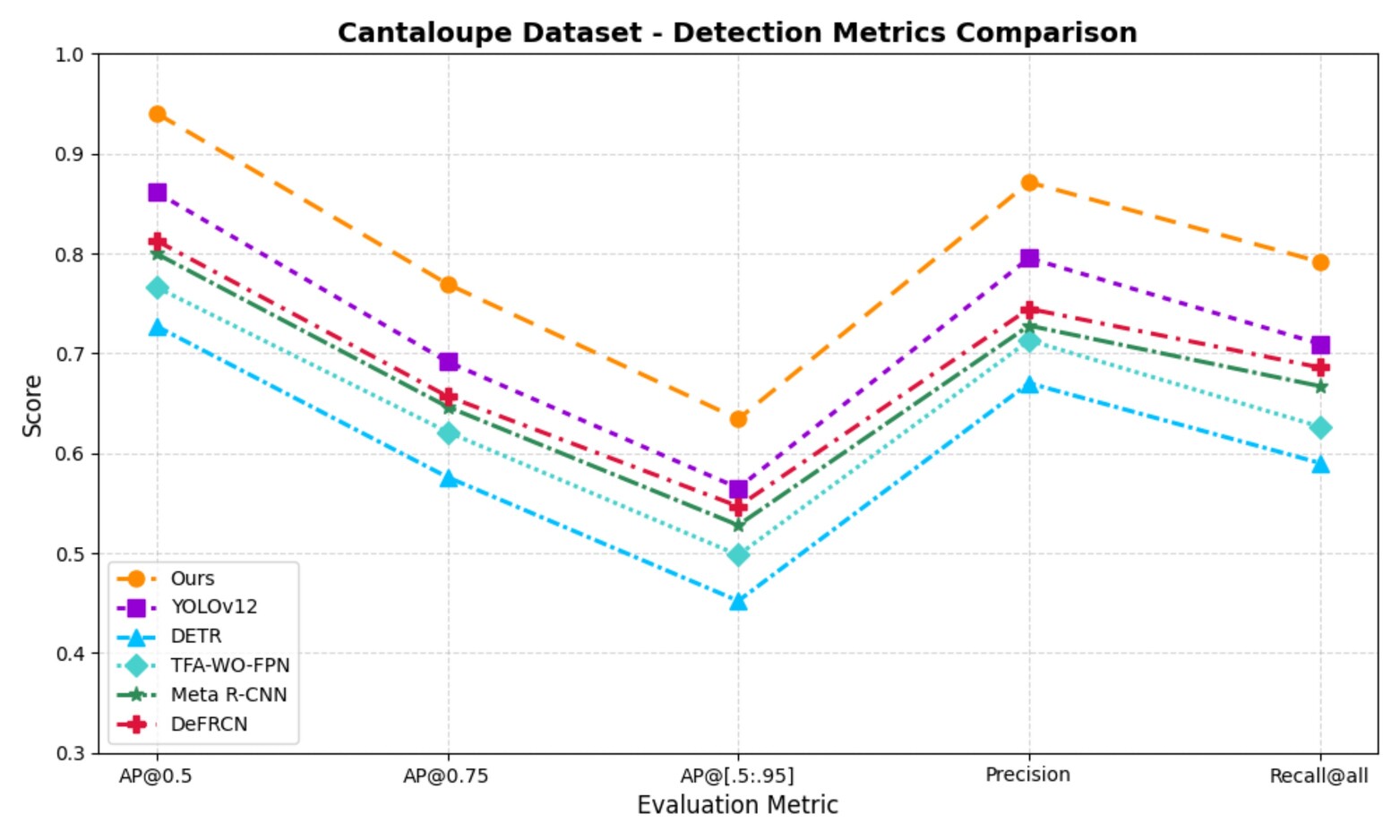

Supplement: Supplementary file 1 [file DataSheet1.zip › Fig6.jpg]

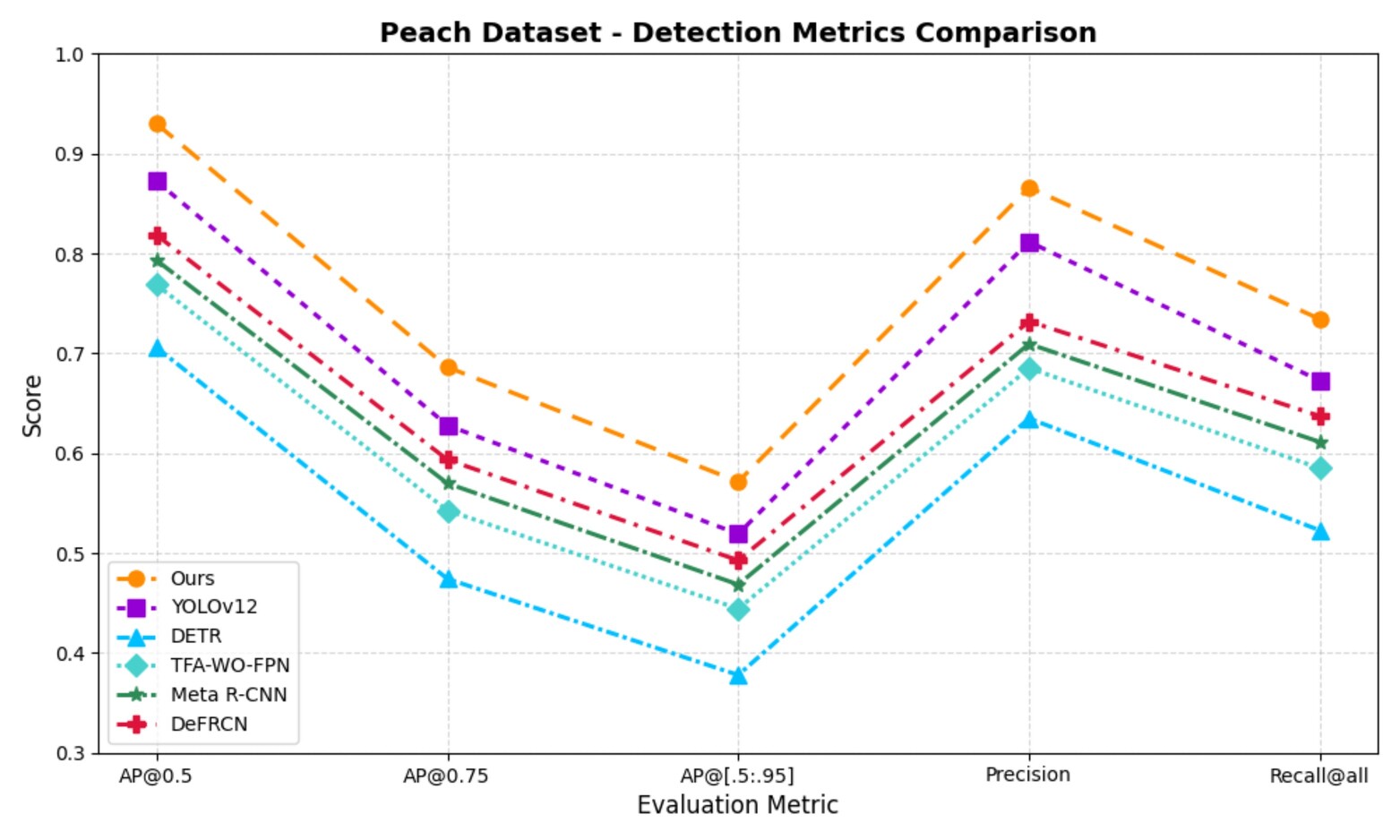

Supplement: Supplementary file 1 [file DataSheet1.zip › Fig7.jpg]

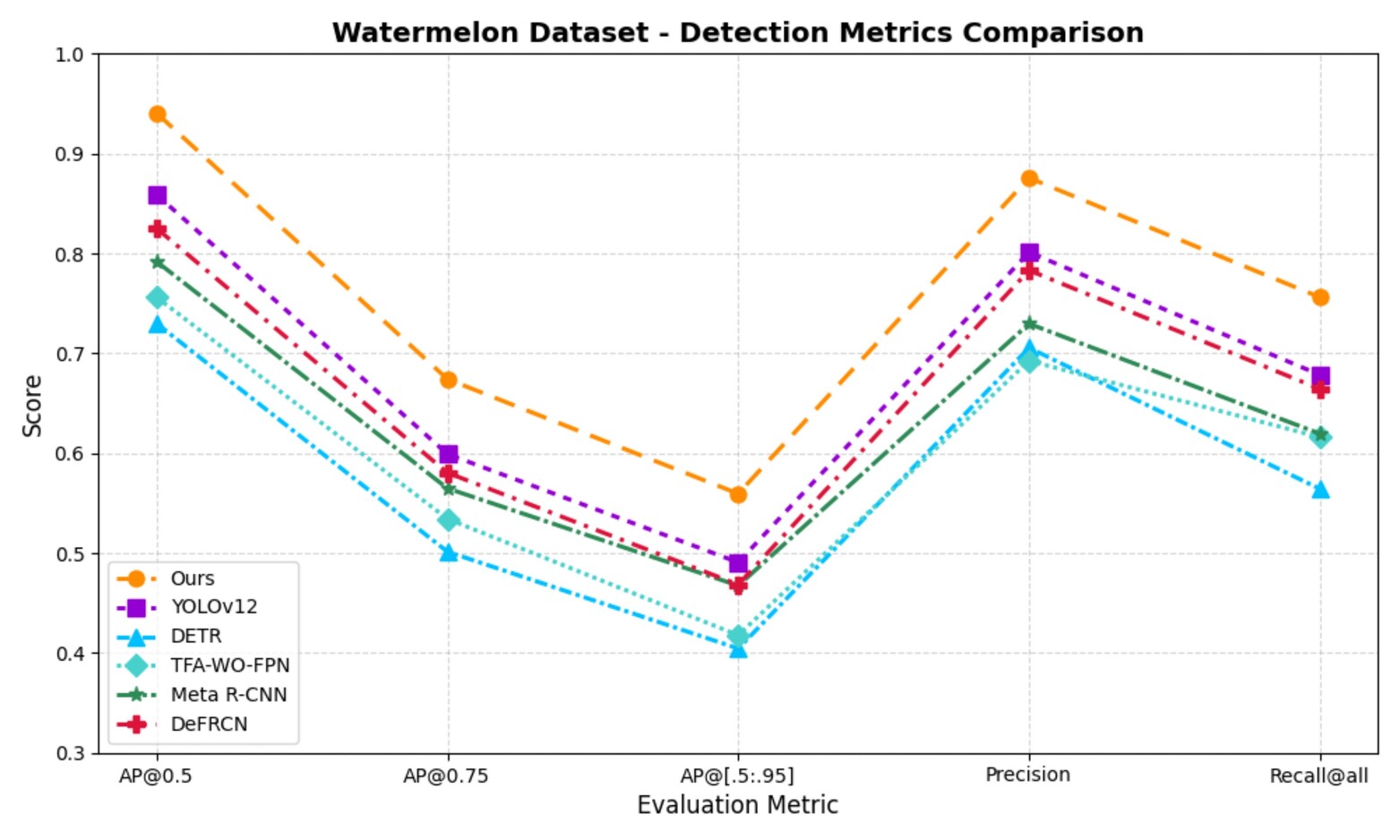

Supplement: Supplementary file 1 [file DataSheet1.zip › Fig8.jpg]

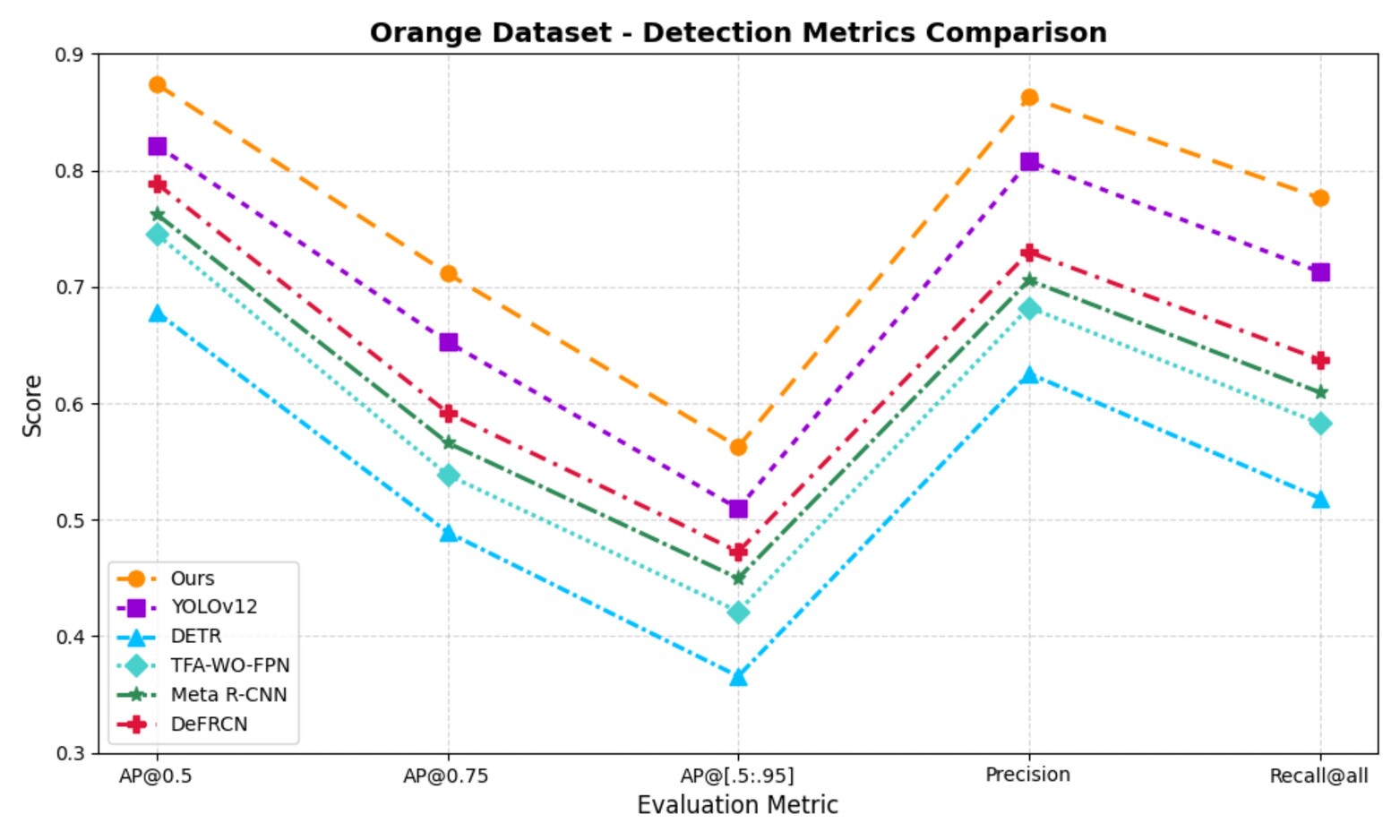

Supplement: Supplementary file 1 [file DataSheet1.zip › Fig9.jpg]
