# Supplementary material for: A lightweight tri-modal few-shot detection framework for fruit diversity recognition toward digital orchard archiving
Source: Front Plant Sci. 2025 Dec 1;16:1696622. doi: 10.3389/fpls.2025.1696622 (PMC12703783; doi:10.3389/fpls.2025.1696622)
Supplement: Supplementary file 2 [file DataSheet2.pdf]

frontiers

Appendix of "A lightweight tri-modal few-shot detection framework for fruit diversity recognition toward digital orchard archiving "

Huaqiang Xu [1†], Honghan Li [1†], and Ji Zhao [1*]

[1] *School of Computer Science and Software Engineering, University of Science and Technology Liaoning, Anshan, 114051, China.;*

Correspondence*:
Ji Zhao
zhaoji_ustl@outlook.com

APPENDIX

A.1 Extended Comparison with State-of-the-Art Segmentation Methods

This supplementary material provides the extended segmentation experiments that were newly added to support the quantitative evaluation of the CLIP+SAM framework. Since the primary detection dataset does not include pixel-level annotations, it is not feasible to train or directly evaluate segmentation algorithms within that context. To enable quantitative validation, we constructed an auxiliary fruit segmentation dataset consisting of 207 training images, 59 validation images, and 31 test images, all containing pixel-wise labels of apple instances captured under orchard conditions.

Several state-of-the-art fully supervised segmentation models were trained on this dataset, including U-Net, DeepLabV3, FPN, and PSPNet. All baselines were trained under identical experimental settings for fair comparison (AdamW optimizer, learning rate 5×10^{-5}, batch size 4, 50 epochs). Our CLIP+SAM pipeline, in contrast, is training-free: CLIP provides text-conditioned point prompts (e.g."an apple") that guide SAM to produce class-agnostic masks in a zero-shot manner without any task-specific fine-tuning.

The quantitative performance comparison is presented in Table 1, and the corresponding training dynamics and qualitative results are illustrated in Figures 1 and 2, respectively. Despite operating in a training-free setting, the proposed CLIP+SAM framework achieves consistently higher accuracy and clearer boundary localization than all four supervised baselines. These results demonstrate that the CLIP-guided semantic localization, coupled with SAM's contour-aware mask generation, provides robust and transferable structural priors even without explicit supervision.

A.2 Analysis of Dataset Split Ratios and Statistical Validation

To analyze the impact of different dataset split ratios on model performance, we have created Table 2, which presents a comparison between the 7:2:1 and 8:1:1 dataset splits across four datasets (Cantaloupe.v2, Orange.v8, Peach.v3, Watermelon.v2). The table shows the changes in AP@0.5, Precision, Recall, t-values, and p-values. The results indicate that while there are slight performance variations across datasets, the

Table 1. Quantitative comparison on the apple segmentation test set.

Method	Training	mIoU	mDice	mPA	FWIoU	BF
U-Net	✓	0.78	0.87	0.84	0.88	0.50
DeepLabV3	✓	0.77	0.86	0.84	0.87	0.37
FPN	✓	0.71	0.81	0.77	0.84	0.31
PSPNet	✓	0.74	0.84	0.80	0.86	0.29
**Ours (CLIP+SAM)**	✗	**0.92**	**0.96**	**0.94**	**0.93**	**0.90**

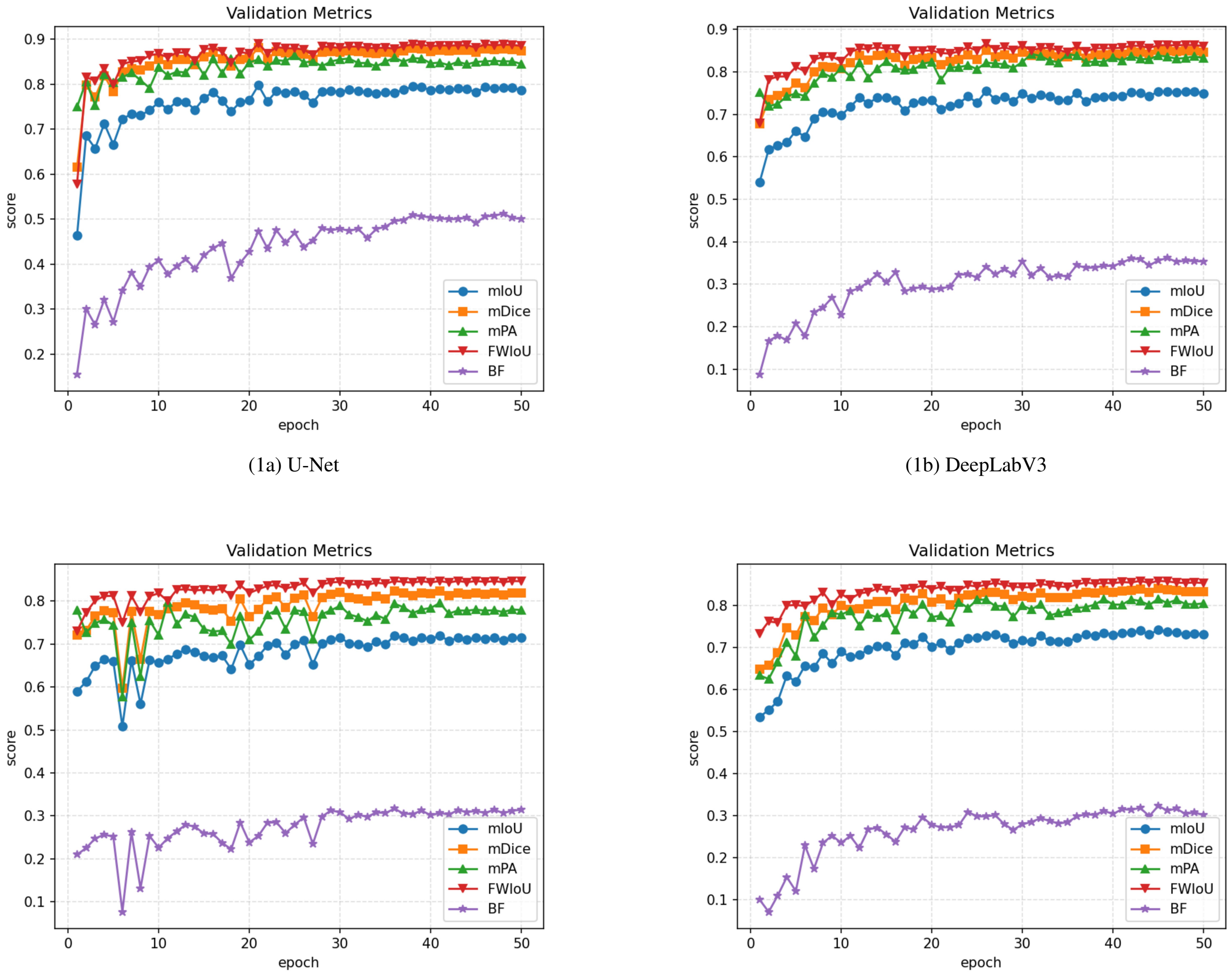

(1a) U-Net

(1b) DeepLabV3

(1c) FPN

(1d) PSPNet

Figure 1. Validation curves of four supervised segmentation baselines across training epochs. Each subfigure shows the evolution of five evaluation metrics (mIoU, mDice, mPA, FWIoU, BF) for U-Net, DeepLabV3, FPN, and PSPNet respectively.

t-test results demonstrate no significant difference (p-values greater than 0.40), suggesting that the model is robust to changes in the dataset split ratio.

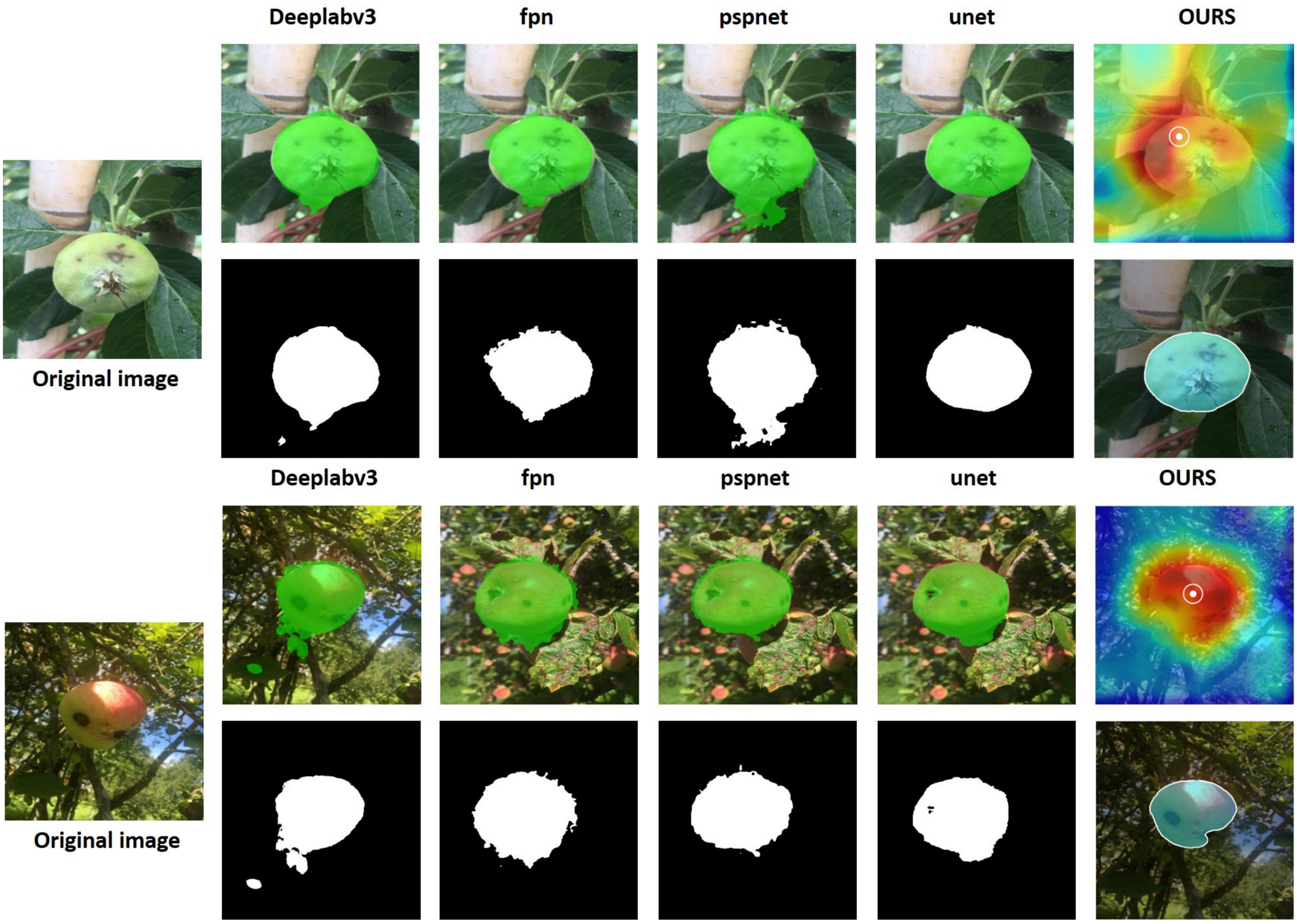

Figure 2. Qualitative comparison of segmentation results among five methods: four supervised baselines (DeepLabV3, FPN, PSPNet, and U-Net) and the proposed CLIP+SAM framework. Each group shows an original orchard image (left), followed by segmentation outputs from all models.

Table 2. Comparison between 7:2:1 and 8:1:1 dataset splits and corresponding statistical results.

Dataset	Direction	ΔAP@0.5	ΔPrecision	ΔRecall	*t*-value	*p*-value
Cantaloupe.v2	7:2:1 → 8:1:1	+0.012	-0.006	+0.008	0.86	0.41
Orange.v8	7:2:1 → 8:1:1	-0.008	+0.010	-0.005	0.68	0.51
Peach.v3	8:1:1 → 7:2:1	+0.007	-0.005	+0.009	0.73	0.47
Watermelon.v2	8:1:1 → 7:2:1	-0.010	+0.007	-0.008	0.81	0.45

A.3 Evaluation on Public Fruit Datasets

To further validate the generalization ability of the proposed framework beyond the self-built orchard dataset, we additionally evaluated it on public fruit datasets derived from the *Fruits & Vegetable Detection* dataset available on Kaggle. Among the multiple fruit categories included in the original dataset, two representative subsets—Apple and Durian—were selected for experimental evaluation. Both subsets contain visually complex instances characterized by high intra-class variance, natural occlusions, and illumination diversity, which provide a challenging benchmark for few-shot detection models.

Table 3 presents the quantitative comparison of our method against five representative few-shot object detection (FSOD) baselines, including YOLOv12, DeFRCN, Meta R-CNN, TFA-WO-FPN, and DETR.

Table 3. Results on public fruit subsets (Apple and Durian).

Model	AP@0.5	AP@0.75	AP@[.50:.95]	Precision	Recall
**Apple**					
Ours	**0.835**	**0.748**	**0.616**	**0.862**	**0.739**
YOLOv12	0.762	0.703	0.583	0.803	0.688
DeFRCN	0.732	0.676	0.559	0.782	0.661
Meta R-CNN	0.711	0.652	0.538	0.769	0.642
TFA-WO-FPN	0.698	0.639	0.523	0.757	0.628
DETR	0.628	0.575	0.476	0.702	0.568
**Durian**					
Model	AP@0.5	AP@0.75	AP@[.50:.95]	Precision	Recall
Ours	**0.928**	**0.769**	**0.629**	**0.879**	**0.834**
YOLOv12	0.865	0.715	0.579	0.835	0.794
DeFRCN	0.826	0.682	0.554	0.812	0.765
Meta R-CNN	0.797	0.661	0.537	0.798	0.738
TFA-WO-FPN	0.777	0.643	0.519	0.785	0.721
DETR	0.693	0.585	0.457	0.751	0.672

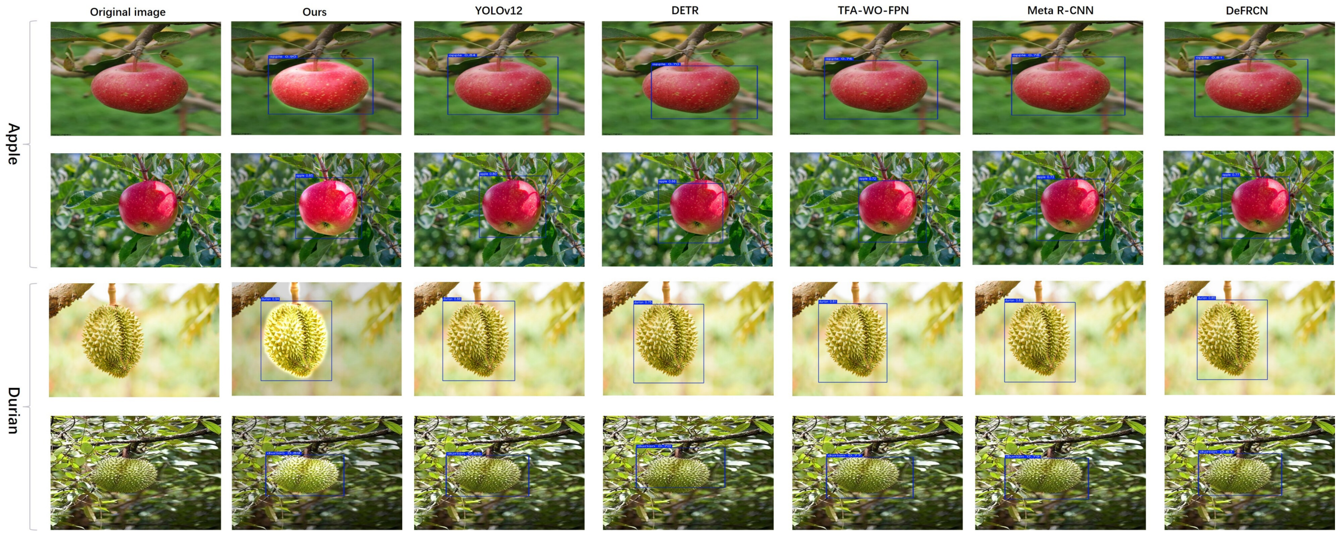

Figure 3. Comparative detection results on public fruit subsets (Apple and Durian) from the Kaggle Fruits & Vegetable Detection dataset.

Our approach achieves the highest scores across all evaluation metrics (AP@0.5, AP@0.75, AP@[.50:.95], Precision, and Recall) for both the Apple and Durian subsets.

As illustrated in Figure 3, our model produces tighter and more complete bounding boxes under challenging conditions such as partial occlusion, dense foliage, and complex illumination. In contrast, baseline models often show incomplete detections or false positives, particularly in cluttered orchard backgrounds. These results confirm that the proposed multimodal fusion strategy effectively generalizes to unseen public data, demonstrating strong robustness and cross-domain transferability.

To further interpret the contribution of each module, an ablation study was conducted on the same public subsets. Table 4 summarizes the performance evolution as key modules (MSA, FER, CLIP, and AWE) were progressively integrated into the YOLOv12 baseline. Consistent with our main experiments,

Table 4. Ablation on public fruit subsets (Apple and Durian).

**Apple subset**	AP@0.5	AP@0.75	AP@[.50:.95]	Precision	Recall@all
Baseline	0.762	0.703	0.583	0.803	0.688
+ MSA	0.769	0.709	0.589	0.811	0.692
+ MSA + FER	0.802	0.731	0.605	0.836	0.714
+ MSA + FER + CLIP	0.824	0.744	0.612	0.852	0.729
**+ MSA + FER + CLIP + AWE**	**0.835**	**0.748**	**0.616**	**0.862**	**0.739**
**Durian subset**	AP@0.5	AP@0.75	AP@[.50:.95]	Precision	Recall@all
Baseline	0.865	0.715	0.579	0.835	0.794
+ MSA	0.872	0.723	0.586	0.841	0.799
+ MSA + FER	0.905	0.749	0.606	0.861	0.818
+ MSA + FER + CLIP	0.918	0.761	0.621	0.873	0.828
**+ MSA + FER + CLIP + AWE**	**0.928**	**0.769**	**0.629**	**0.879**	**0.834**

the Mask-Saliency Adapter (MSA) yields a slight but stable improvement by enhancing local attention. Introducing the Feature Enhancement Recomposer (FER) provides a more substantial gain, confirming the benefits of residual-based feature modulation. The addition of CLIP-guided semantic prompts leads to further enhancement in both precision and recall, while the final AWE mechanism contributes minor yet consistent improvements by adaptively balancing semantic and original pathways. These quantitative results demonstrate that each module contributes synergistically to the overall performance improvement.

Corresponding Grad-CAM visualizations are shown in Figure 4, illustrating the evolution of attention focus across fusion stages. As modules are progressively added, the attention maps become increasingly concentrated on the target fruit regions, highlighting biologically meaningful cues such as shape boundaries and surface textures. This trend provides clear visual evidence that the proposed semantic-guided fusion framework improves not only detection accuracy but also interpretability and spatial consistency in real-world agricultural imagery.

A.4 Dataset Construction and Augmentation Pipeline

Figure 5 illustrates the overall dataset preparation workflow, including data collection from Roboflow and Kaggle, stratified dataset splitting, and hybrid augmentation operations applied only to the training set. The augmentation pipeline integrates color-based (HSV, brightness, contrast), geometric (translation, scaling, rotation), and structure-aware (Mosaic, MixUp, Copy-Paste) strategies to enhance data diversity and model robustness.

We have added a comprehensive Table 5 that clearly lists, for each fruit category: (i) the number of raw images collected, (ii) the exact counts after the final train/validation/test split, (iii) the augmentation multiplier applied during online training, and (iv) the effective training sample size. As shown in the table, our augmentation strategy adaptively applies stronger augmentation (multiplier 3.3–4.1×) to classes with fewer training samples, which helps mitigate class imbalance and improve model generalization.

We have included a comprehensive visualization showing 6 augmentation techniques (HSV color adjustment, brightness, contrast, translation, scaling, and rotation) applied to representative samples from all six fruit categories(see Figure 6). These visual pairs explicitly demonstrate how the original images are transformed during the training process, making the augmentation pipeline transparent and reproducible.

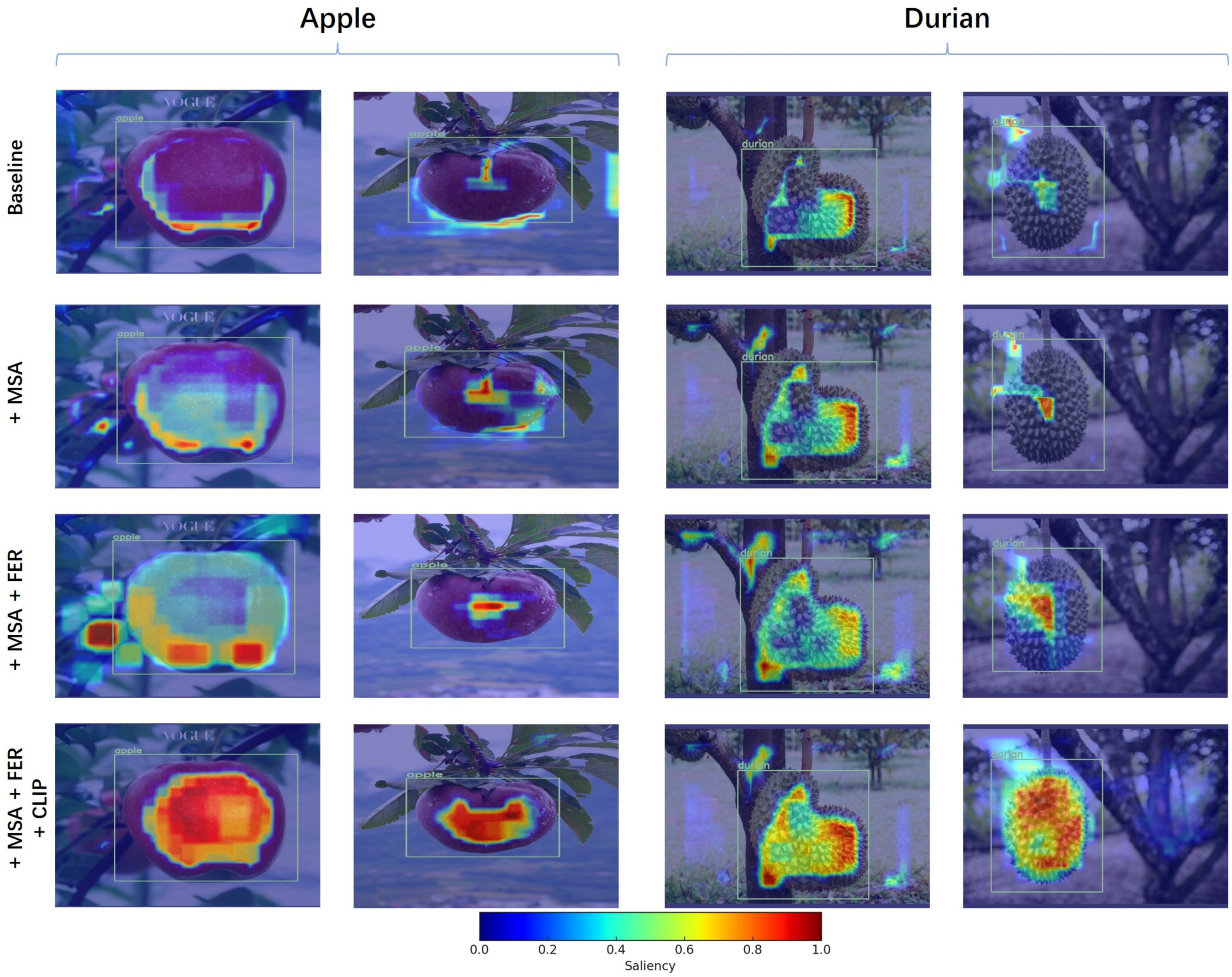

Figure 4. Grad-CAM visualization of attention evolution across ablation configurations on the public Apple and Durian subsets.

Table 5. Dataset Composition and Augmentation Statistics.

Category	Original	Train	Valid	Test	Augmentation	Effective Samples
Cantaloupe	220	152	45	23	3.6	547
Peach	209	165	30	14	3.3	545
Watermelon	172	140	21	11	3.8	532
Orange	165	120	29	16	4.1	492
Apple	213	149	42	22	3.5	522
Durian	195	156	19	20	3.4	530

A.5 Additional Experiments on Peach-A and Peach-B Datasets

To address the need for more challenging scenarios with severe occlusion and multiple samples per image, we conducted additional experiments on two newly introduced datasets: *Peach-A* and *Peach-B*. These datasets contain 217 and 205 images respectively, featuring more complex real-world orchard conditions. We organized both datasets with a 7:2:1 train-validation-test split. We maintained consistent experimental

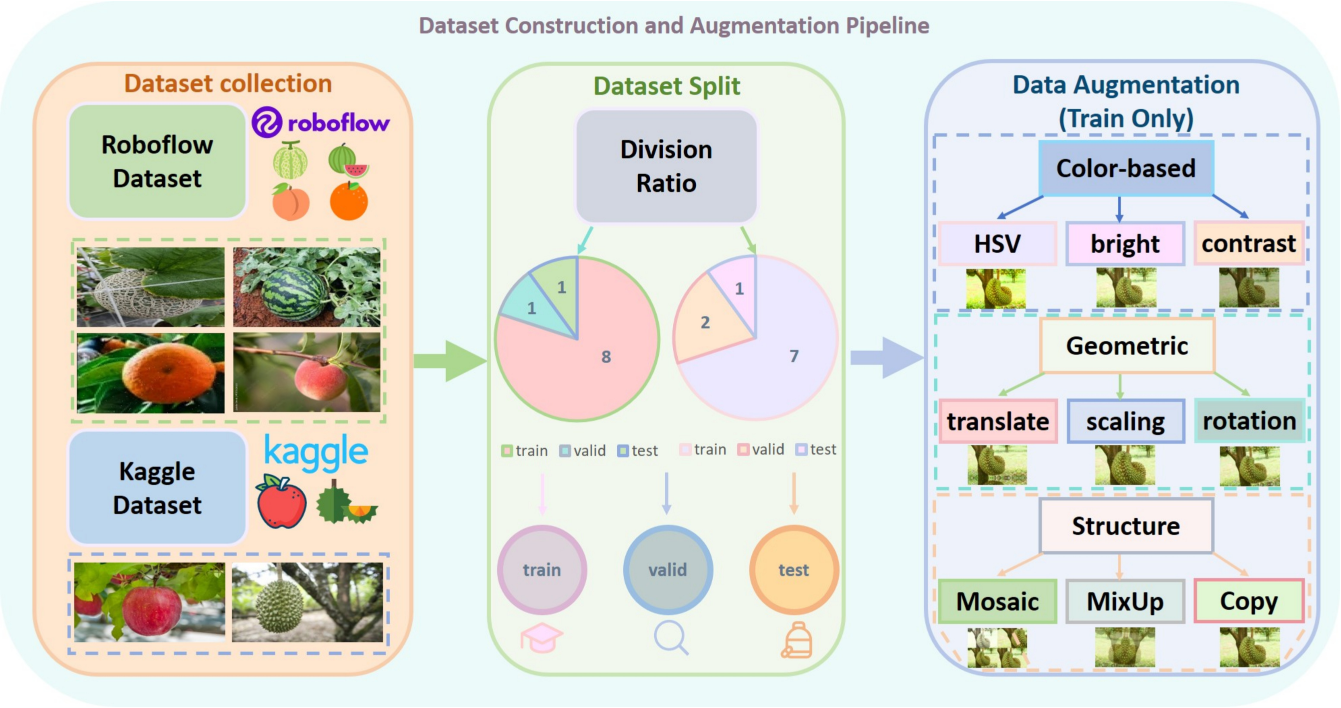

Figure 5. Dataset Construction and Augmentation Pipeline.

settings across both datasets to ensure fair comparison. Specifically, *Peach-A* includes 152 training images, 43 validation images, and 22 test images, while *Peach-B* contains 143 training images, 41 validation images, and 21 test images.

The Figure 7 presents a comparison of segmentation results between CLIP-guided SAM and standalone SAM on two peach datasets, *Peach01* and *Peach02*. The first column shows the original images, while the second column visualizes the CLIP attention map, highlighting regions with high attention values. The third column demonstrates the segmentation masks generated by SAM using CLIP-derived prompts, while the fourth column displays the results of standalone SAM without any guidance. The segmentation masks produced by the CLIP-guided SAM are clearly more focused and semantically aligned with the target fruit regions, compared to the over-segmented and often noisy results from the standalone SAM. This visualization highlights the effectiveness of CLIP in guiding SAM to produce more accurate and reliable segmentation outputs in the context of complex orchard scenes.

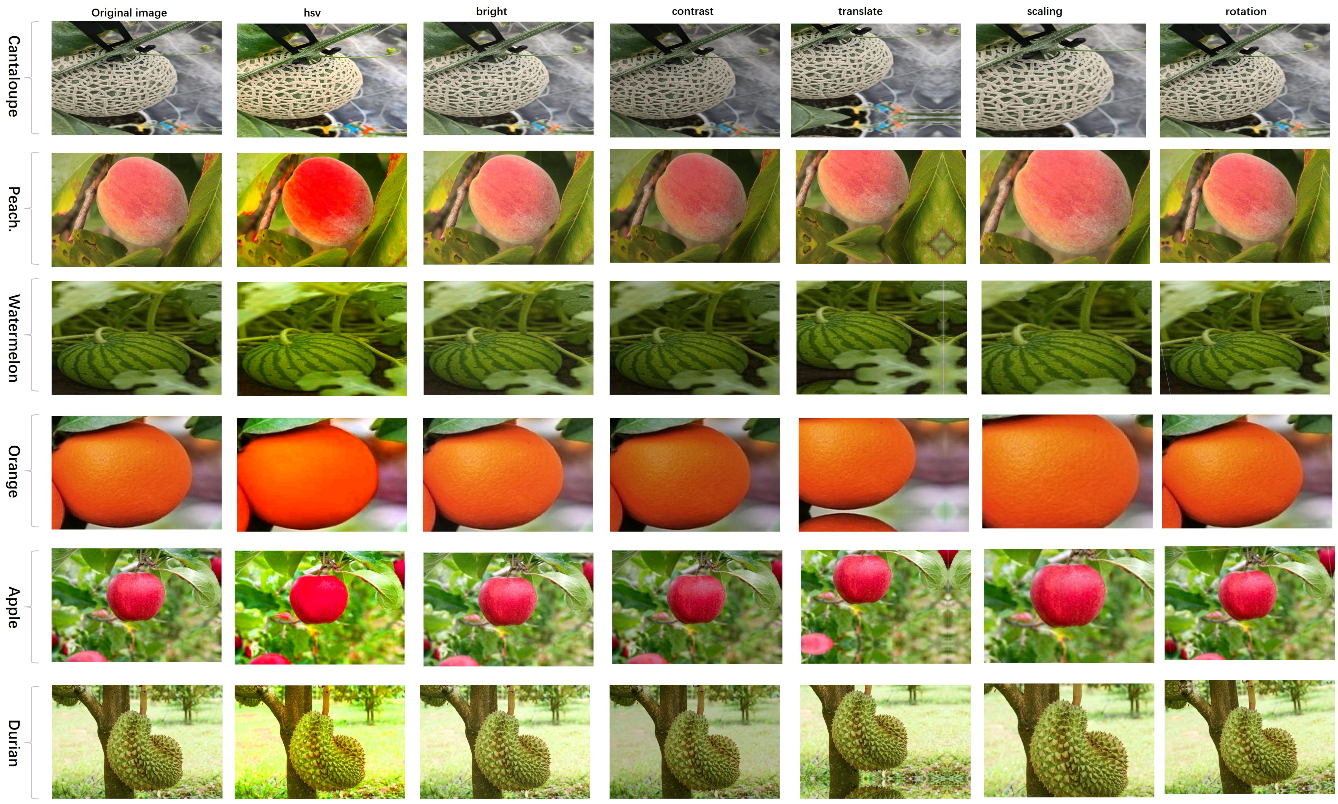

Figure 6. Data augmentation examples on different fruit datasets.

Table 6. Detection performance on two new peach datasets.

**Dataset PEACH-A**	AP@0.5	AP@0.75	AP@[.50:.95]	Precision	Recall@all
Ours	**0.8852**	**0.6421**	**0.5327**	**0.8524**	**0.7583**
YOLOv12	0.8175	0.5822	0.4812	0.8042	0.6944
DETR	0.7013	0.4724	0.3708	0.6301	0.5204
TFA-WO-FPN	0.7532	0.5317	0.4219	0.6816	0.5778
Meta R-CNN	0.7814	0.5565	0.4452	0.7039	0.6017
EFCN DeFRCN	0.8048	0.5736	0.4639	0.7271	0.6282
**Dataset PEACH-B**	AP@0.5	AP@0.75	AP@[.50:.95]	Precision	Recall@all
Ours	**0.8764**	**0.6353**	**0.5251**	**0.8475**	**0.7512**
YOLOv12	0.8283	0.5941	0.4760	0.7839	0.6863
DETR	0.6945	0.4658	0.3662	0.6278	0.5179
TFA-WO-FPN	0.7471	0.5240	0.4176	0.6775	0.5738
Meta R-CNN	0.7742	0.5496	0.4413	0.6991	0.5974
DeFRCN	0.7985	0.5671	0.4592	0.7228	0.6236

Bold values denote the highest score in each metric for each dataset.

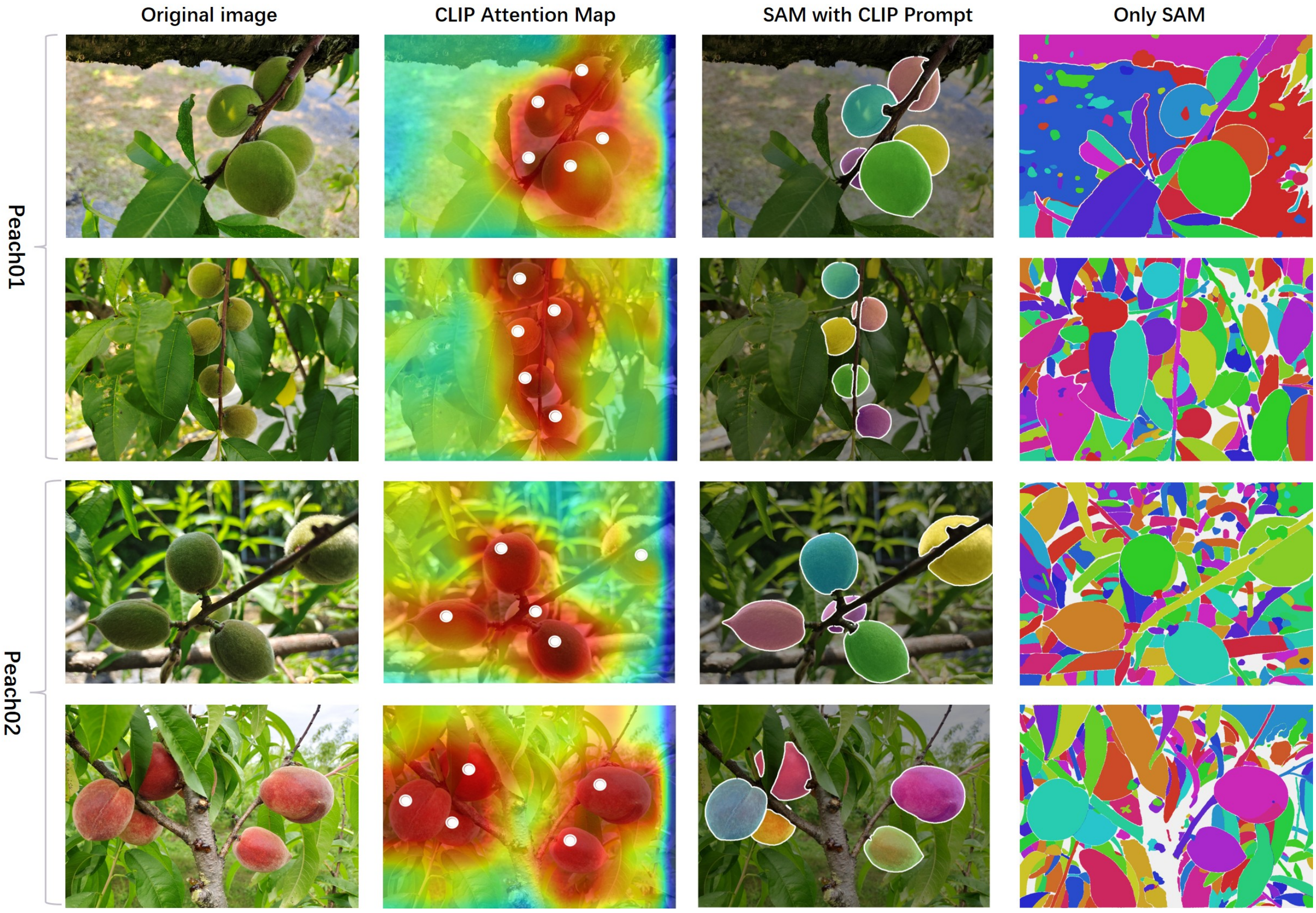

Figure 7. Segmentation comparison between CLIP-guided and standalone SAM on peach datasets.

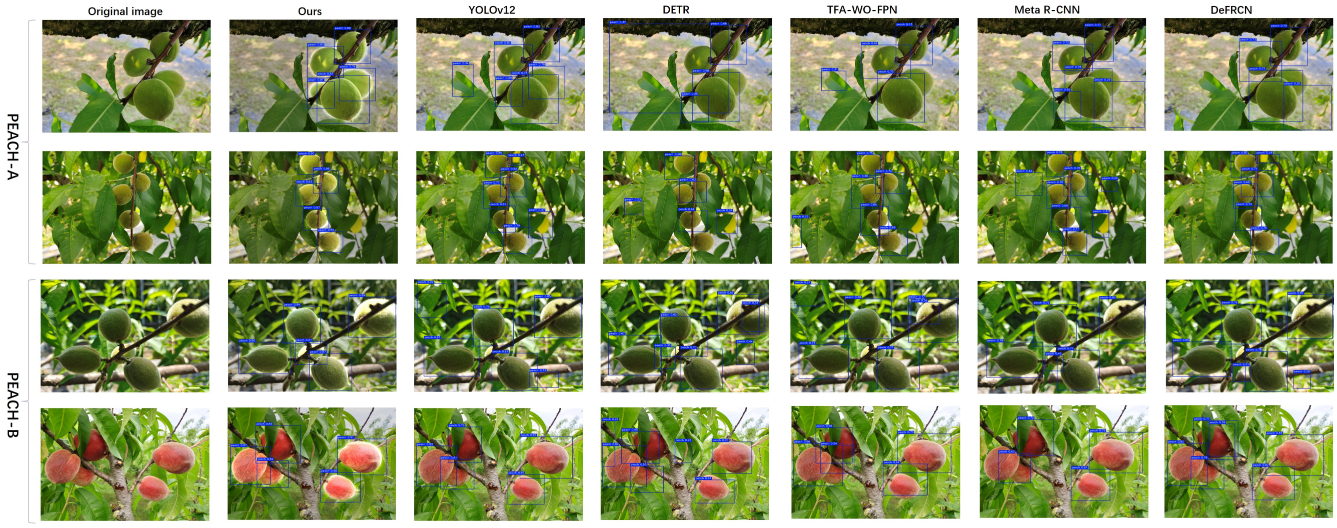

Figure 8. Detection visualization on PEACH-A and PEACH-B datasets.

The Table 6 and Figure 8 above present the detection performance and visual results of the proposed model on two new peach datasets: *Peach-A* and *Peach-B*. The table summarizes the detection accuracy of our method compared to several established baselines, including YOLOv12, DETR, TFA-WO-FPN, Meta R-CNN, and DeFRCN. Our approach outperforms the baselines across all evaluation metrics, achieving the

Table 7. Ablation study results on two new peach datasets.

**Dataset PEACH-A**	AP@0.5	AP@0.75	AP@[.50:.95]	Precision	Recall@all
Baseline	0.8175	0.5822	0.4812	0.8042	0.6944
+ MSA	0.8257	0.5896	0.4871	0.8053	0.7018
+ MSA + FER	0.8536	0.6153	0.5137	0.8301	0.7312
+ MSA + FER + CLIP	0.8798	0.6335	0.5305	0.8493	0.7544
+ MSA + FER + CLIP + AWE	**0.8852**	**0.6421**	**0.5327**	**0.8524**	**0.7583**
**Dataset PEACH-B**	AP@0.5	AP@0.75	AP@[.50:.95]	Precision	Recall@all
Baseline	0.8283	0.5941	0.4760	0.7839	0.6863
+ MSA	0.8342	0.5995	0.4824	0.7913	0.6948
+ MSA + FER	0.8563	0.6128	0.4983	0.8189	0.7237
+ MSA + FER + CLIP	0.8737	0.6335	0.5147	0.8406	0.7386
+ MSA + FER + CLIP + AWE	**0.8764**	**0.6353**	**0.5251**	**0.8475**	**0.7512**

Bold values denote the highest score in each metric for each dataset.

highest scores in terms of AP@0.5, AP@0.75, AP@[.50:.95], Precision, and Recall@all for both datasets. Specifically, on the *Peach-A* dataset, our method achieves an AP@0.5 of 0.8852, and on the *Peach-B* dataset, it achieves an AP@0.8764.

The Figure 8 provides a visual comparison of the detection results on both *Peach-A* and *Peach-B* datasets, illustrating the model's ability to accurately detect peach fruits even in challenging orchard scenes. These results confirm that our method handles occlusion, overlapping, and varying lighting conditions effectively, showing a marked improvement over other state-of-the-art methods. The high detection performance in real-world agricultural conditions further demonstrates the robustness and practicality of the proposed framework.

The Table 7 and Figure 9 above present the ablation study results and the corresponding saliency visualizations on the *Peach-A* and *Peach-B* datasets. The table summarizes the detection performance of the proposed model under different configurations, including the baseline YOLOv12 model, the addition of the Mask-Saliency Adapter (MSA), the Feature Enhancement Recomposer (FER), the CLIP-guided semantic prompts, and the final configuration with the Attention-Aware Weight Estimator (AWE). The results show consistent improvements across all evaluation metrics, with the final model achieving the best performance on both datasets. Specifically, on the *Peach-A* dataset, the AP@0.5 score improves from 0.8175 to 0.8852, and on the *Peach-B* dataset, it improves from 0.8283 to 0.8764, highlighting the effectiveness of each component.

The accompanying Figure 9 visually demonstrates the evolution of saliency maps across different fusion stages. The saliency maps, shown for both *Peach-A* and *Peach-B*, illustrate the progressive enhancement in attention localization as more components are added. The baseline model produces a weak and sparse saliency map, while the addition of MSA and FER improves the focus on the target object. The integration of CLIP further refines the spatial attention by providing category-specific guidance, which is clearly reflected in the final saliency map. These visualizations confirm that our approach successfully directs attention to semantically relevant regions, improving both detection and segmentation accuracy.

A.6 Training Strategy for 2:1:7 Data Split

To validate our model's core innovation of achieving superior performance with minimal training data, we designed a specialized two-stage training strategy for the 2:1:7 (20% training, 10% validation, 70%

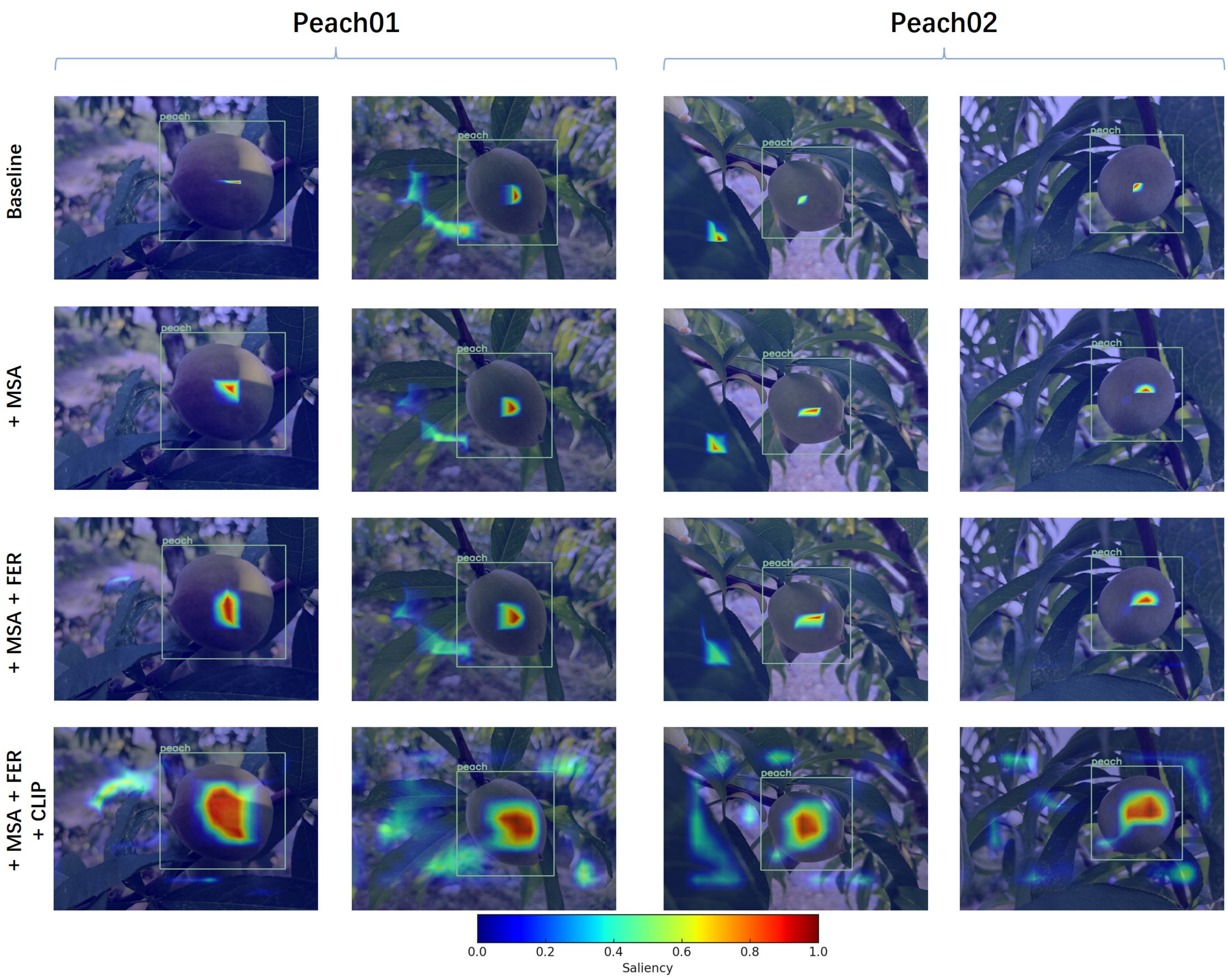

Figure 9. Attention evolution across fusion stages on two peach datasets.

test) data split scenario. This extreme small-sample setting poses significant challenges in terms of model overfitting and generalization.

In the first stage, we freeze the backbone network (first 10 layers) and only fine-tune the detection head and neck components. We employ the AdamW optimizer with an initial learning rate of 5×10^{-4}, cosine annealing schedule with 8-epoch warmup, and weight decay of 0.01 for regularization. To prevent overfitting on the limited training samples, we adopt a conservative data augmentation strategy including HSV adjustment ($h = 0.015, s = 0.6, v = 0.3$), geometric transforms (translation 0.05, scale 0.2, shear 2.0°), horizontal and vertical flipping (0.5 and 0.05 probability respectively), mosaic augmentation (0.3 probability, closed after epoch 7), mixup (0.1 probability), and minimal copy-paste (0.05 probability). This stage runs for 20 epochs, approximately one-third of the total training budget.After the detection head converges in Stage 1, we unfreeze the entire backbone and perform end-to-end fine-tuning with a reduced learning rate of 2×10^{-4} (40% of Stage 1) to prevent catastrophic forgetting. The learning rate schedule follows cosine annealing with a 5-epoch warmup, and weight decay is maintained at 0.01 for consistency. We further reduce the augmentation intensity to preserve learned features: HSV adjustment ($h = 0.01, s = 0.5, v = 0.25$), geometric transforms (translation 0.03, scale 0.15, shear 1.0°), mosaic

augmentation (0.2 probability, closed after epoch 27), reduced mixup (0.05 probability), and disabled copy-paste to avoid introducing noise. This stage continues for the remaining 80 epochs, allowing the model to adapt fully to the small-sample training set while maintaining generalization capability.

Additionally, to validate the effectiveness of the new training process, we conducted a T-test comparing the performance of the model with the new and old training setups on our original dataset. The results of this comparison show no significant differences, assuring the stability of the model's performance under both training setups(see Table 8).

Table 8. Comparison between new and old training methods and corresponding statistical results.

Dataset	Training Method	ΔAP@0.5	ΔPrecision	ΔRecall	t-value	p-value
Cantaloupe.v2	Old $\rightarrow$ New	+0.010	-0.007	+0.009	0.82	0.42
Orange.v8	Old $\rightarrow$ New	-0.003	+0.005	-0.002	0.68	0.50
Peach.v3	Old $\rightarrow$ New	+0.006	-0.004	+0.008	0.75	0.46
Watermelon.v2	Old $\rightarrow$ New	-0.007	+0.003	-0.006	0.79	0.44

We have conducted experiments using this adjusted 2:1:7 split, and the specific experimental results are presented in Table 9, Figure 10, Table 10, and Figure 11.

This Table 9 presents the detection performance of different methods across four fruit datasets: Cantaloupe. v2, Peach. v3, Watermelon. v2, and Orange.v8. For each dataset, we compare our method to several established baselines, including YOLOv12, DETR, TFA-WO-FPN, Meta R-CNN, and DeFRCN. The metrics include AP@0.5, AP@0.75, AP@[.50:. 95], Precision, and Recall@all, with bold values highlighting the best performance in each metric. As shown, our method consistently outperforms the baseline models in terms of both precision and recall, demonstrating the effectiveness of our approach in fruit detection tasks.

The Figure 10 provides detection visualizations for the 2:1:7 dataset split on the four fruit datasets (Cantaloupe.v2, Peach.v3, Watermelon.v2, and Orange.v8). It compares the performance of our method with the baseline YOLOv12, DETR, TFA-WO-FPN, Meta R-CNN, and DeFRCN on these datasets. The visual results clearly demonstrate that our method handles the challenging scenarios posed by occlusion, overlapping, and varying lighting conditions effectively, showcasing its superior detection capabilities compared to the other models.

This Table 10 presents the results of an ablation study on four fruit datasets—Cantaloupe. v2, Peach. v3, Watermelon. v2, and Orange.v8. The table compares the performance of our method with different configurations, including variations of Multi-Scale Attention (MSA), Feature Expansion Refinement (FER), CLIP integration, and Attention Weight Expansion (AWE). We observe that the performance improves consistently across multiple metrics (AP@0.5, AP@0.75, AP@[.5:. 95], Precision, Recall) as we incorporate additional components into the model. The bold values represent the best performance achieved for each dataset. Notably, the integration of CLIP and AWE leads to the highest scores, demonstrating the effectiveness of these additions in fruit detection.

The Figure 11 illustrates the evolution of attention maps across different fusion stages for the four datasets. It visualizes the impact of different model components—Baseline, MSA, MSA + FER, and MSA + FER + CLIP—on the attention distribution. As shown in the figure, incorporating MSA, FER, and CLIP significantly refines the attention maps, allowing the model to focus more accurately on the target objects.

Table 9. Detection performance of different methods on four fruit datasets.

**Dataset CANTALOUPE**	AP@0.5	AP@0.75	AP@[.50:.95]	Precision	Recall@all
Ours	**0.8847**	**0.7128**	**0.5894**	**0.8293**	**0.7451**
YOLOv12	0.8015	0.6342	0.5187	0.7486	0.6589
DETR	0.6742	0.5214	0.4095	0.6189	0.5417
TFA-WO-FPN	0.7128	0.5681	0.4536	0.6647	0.5794
Meta R-CNN	0.7453	0.5924	0.4827	0.6815	0.6183
DeFRCN	0.7596	0.6047	0.5015	0.6982	0.6371
**Dataset PEACH**	AP@0.5	AP@0.75	AP@[.50:.95]	Precision	Recall@all
Ours	**0.8731**	**0.6315**	**0.5268**	**0.8217**	**0.6896**
YOLOv12	0.8147	0.5753	0.4746	0.7641	0.6251
DETR	0.6523	0.4285	0.3401	0.5874	0.4782
TFA-WO-FPN	0.7139	0.4951	0.4017	0.6379	0.5392
Meta R-CNN	0.7384	0.5217	0.4259	0.6628	0.5647
DeFRCN	0.7627	0.5436	0.4501	0.6859	0.5918
**Dataset WATERMELON**	AP@0.5	AP@0.75	AP@[.50:.95]	Precision	Recall@all
Ours	**0.8792**	**0.6189**	**0.5147**	**0.8314**	**0.7092**
YOLOv12	0.7981	0.5436	0.4463	0.7528	0.6297
DETR	0.6784	0.4527	0.3651	0.6541	0.5174
TFA-WO-FPN	0.7025	0.4816	0.3784	0.6438	0.5683
Meta R-CNN	0.7361	0.5127	0.4241	0.6819	0.5741
DeFRCN	0.7693	0.5294	0.4287	0.7346	0.6158
**Dataset ORANGE**	AP@0.5	AP@0.75	AP@[.50:.95]	Precision	Recall@all
Ours	**0.8186**	**0.6547**	**0.5179**	**0.8174**	**0.7284**
YOLOv12	0.7628	0.5961	0.4653	0.7592	0.6641
DETR	0.6251	0.4386	0.3294	0.5782	0.4739
TFA-WO-FPN	0.6894	0.4857	0.3826	0.6341	0.5359
Meta R-CNN	0.7081	0.5142	0.4095	0.6583	0.5628
DeFRCN	0.7342	0.5417	0.4319	0.6827	0.5916

Bold values denote the highest score in each metric for each dataset.

This evolution demonstrates the increasing robustness of the model as additional features are integrated, enhancing its ability to detect fruits in challenging conditions, such as varying occlusions and lighting.

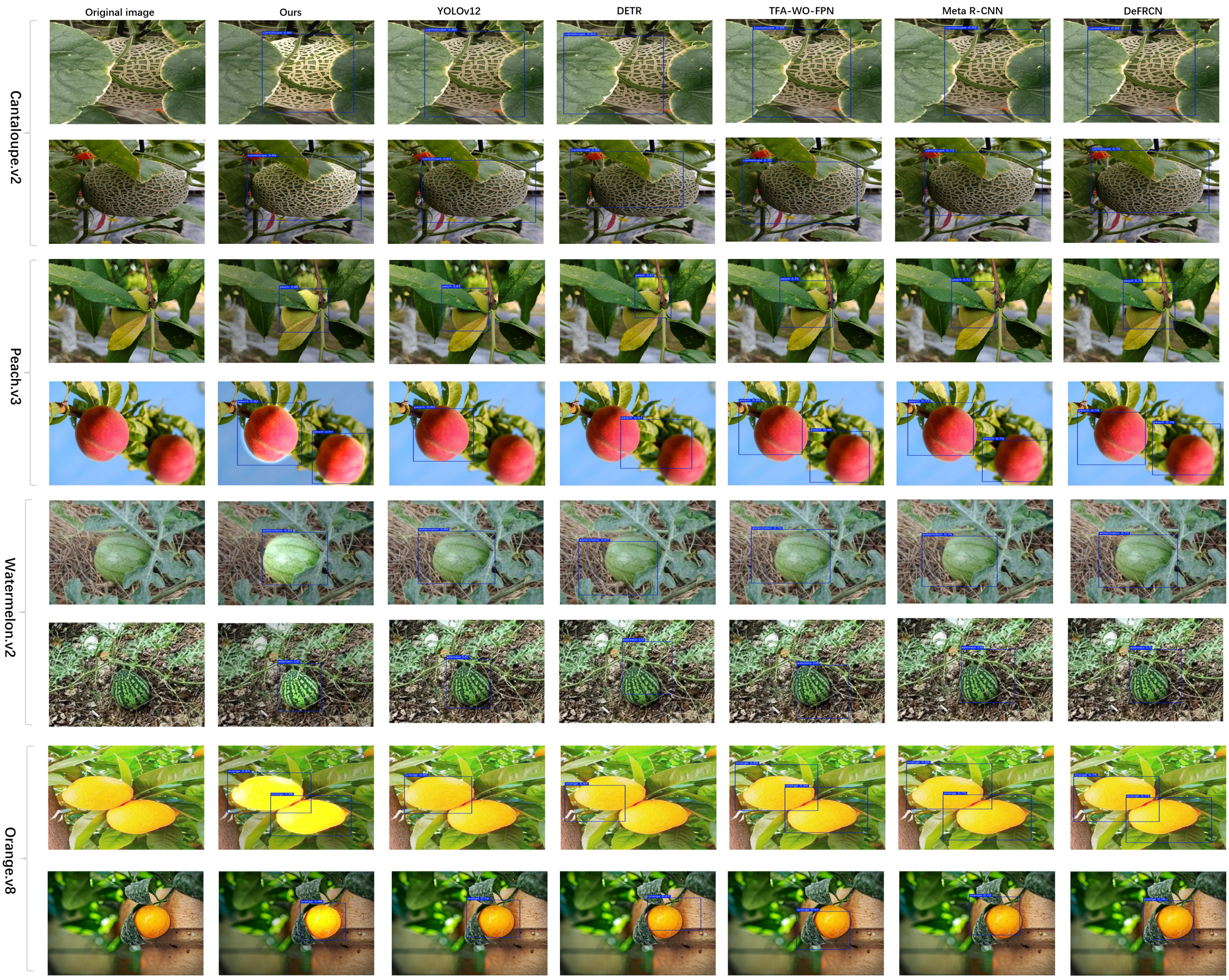

Figure 10. Detection visualization on 2:1:7 datasets.

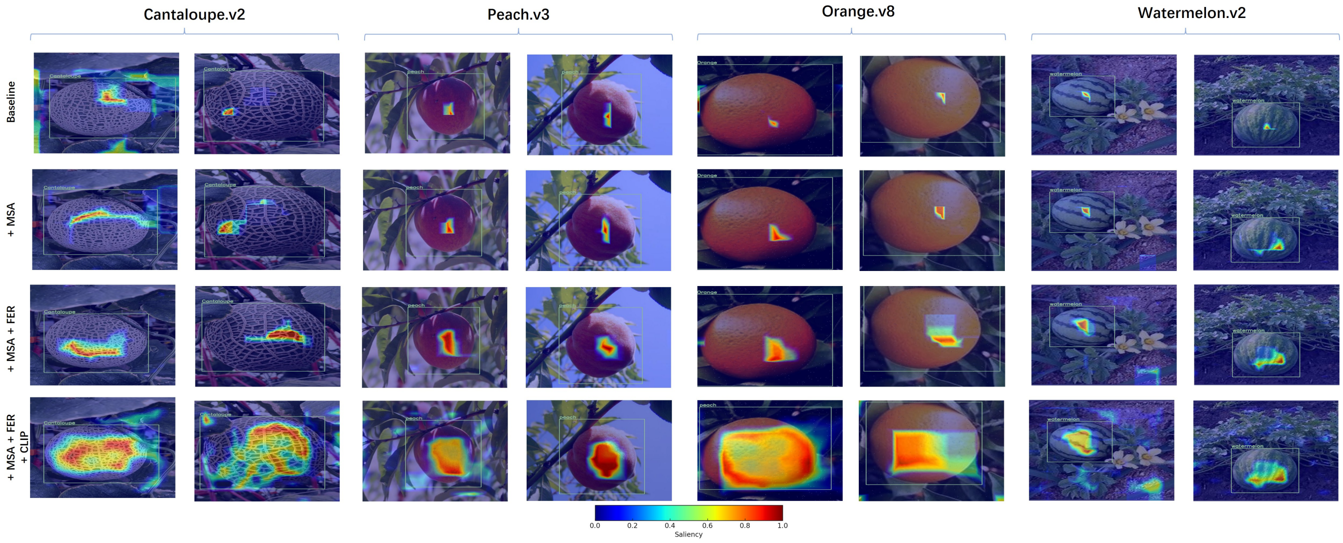

Figure 11. Attention evolution across fusion stages on 2:1:7 datasets.

Table 10. Ablation study on four fruit datasets.

**Dataset CANTALOUPE**	AP@0.5	AP@0.75	AP@[.5:.95]	Precision	Recall@all
Baseline	0.8015	0.6342	0.5187	0.7486	0.6589
+ MSA	0.8083	0.6425	0.5269	0.7581	0.6635
+ MSA + FER	0.8417	0.6751	0.5548	0.7869	0.7046
+ MSA + FER + CLIP	0.8738	0.7024	0.5802	0.8189	0.7354
+ MSA + FER + CLIP + AWE	**0.8847**	**0.7128**	**0.5894**	**0.8293**	**0.7451**
**Dataset PEACH**	AP@0.5	AP@0.75	AP@[.5:.95]	Precision	Recall@all
Baseline	0.8147	0.5753	0.4746	0.7641	0.6251
+ MSA	0.8219	0.5819	0.4815	0.7712	0.6311
+ MSA + FER	0.8534	0.6067	0.5011	0.7968	0.6594
+ MSA + FER + CLIP	0.8692	0.6237	0.5214	0.8140	0.6823
+ MSA + FER + CLIP + AWE	**0.8731**	**0.6315**	**0.5268**	**0.8217**	**0.6896**
**Dataset WATERMELON**	AP@0.5	AP@0.75	AP@[.5:.95]	Precision	Recall@all
Baseline	0.7981	0.5436	0.4463	0.7528	0.6297
+ MSA	0.8057	0.5511	0.4548	0.7604	0.6347
+ MSA + FER	0.8426	0.5842	0.4836	0.7983	0.6703
+ MSA + FER + CLIP	0.8721	0.6104	0.5073	0.8240	0.7008
+ MSA + FER + CLIP + AWE	**0.8792**	**0.6189**	**0.5147**	**0.8314**	**0.7092**
**Dataset ORANGE**	AP@0.5	AP@0.75	AP@[.5:.95]	Precision	Recall@all
Baseline	0.7628	0.5961	0.4653	0.7592	0.6641
+ MSA	0.7681	0.6019	0.4721	0.7656	0.6697
+ MSA + FER	0.7914	0.6254	0.4932	0.7889	0.6951
+ MSA + FER + CLIP	0.8107	0.6468	0.5104	0.8098	0.7203
+ MSA + FER + CLIP + AWE	**0.8186**	**0.6547**	**0.5179**	**0.8174**	**0.7284**

Bold values denote the highest score in each metric for each dataset.
